# Polymerizable Gd(iii) building blocks for the synthesis of high relaxivity macromolecular MRI contrast agents†

**DOI:** 10.1039/d0sc04750c

**Published:** 2021-02-01

**Authors:** Thomas R. Berki, Jonathan Martinelli, Lorenzo Tei, Helen Willcock, Stephen J. Butler

**Affiliations:** Department of Chemistry, Loughborough University Leicestershire LE11 3TU UK s.j.butler@lboro.ac.uk; Department of Materials, Loughborough University Leicestershire LE11 3TU UK h.willcock2@lboro.ac.uk; Department of Science and Technological Innovation, Università del Piemonte Orientale I15121 Alessandria Italy

## Abstract

A new synthetic strategy for the preparation of macromolecular MRI contrast agents (CAs) is reported. Four gadolinium(iii) complexes bearing either one or two polymerizable methacrylamide groups were synthesized, serving as monomers or crosslinkers for the preparation of water-soluble, polymeric CAs using Reversible Addition–Fragmentation Chain Transfer (RAFT) polymerization. Using this approach, macromolecular CAs were synthesized with different architectures, including linear, hyperbranched polymers and gels. The relaxivities of the polymeric CAs were determined by NMR relaxometry, revealing an up to 5-fold increase in relaxivity (60 MHz, 310 K) for the linear polymers compared with the clinically used CA, Gd-DOTA. Moreover, hyperbranched polymers obtained from Gd(iii) crosslinkers, displayed even higher relaxivities up to 22.8 mM^−1^ s^−1^, approximately 8 times higher than that of Gd-DOTA (60 MHz, 310 K). A detailed NMRD study revealed that the enhanced relaxivities of the hyperbranched polymers were obtained by limiting the local motion of the crosslinked Gd(iii) chelate. The versatility of RAFT polymerization of Gd(iii) monomers and crosslinkers opens the doors to more advanced polymeric CAs capable of multimodal, bioresponsive or targeting properties.

## Abstract 2

A new synthetic strategy for the preparation of efficient macromolecular MRI contrast agents is reported.

## Graphical abstract

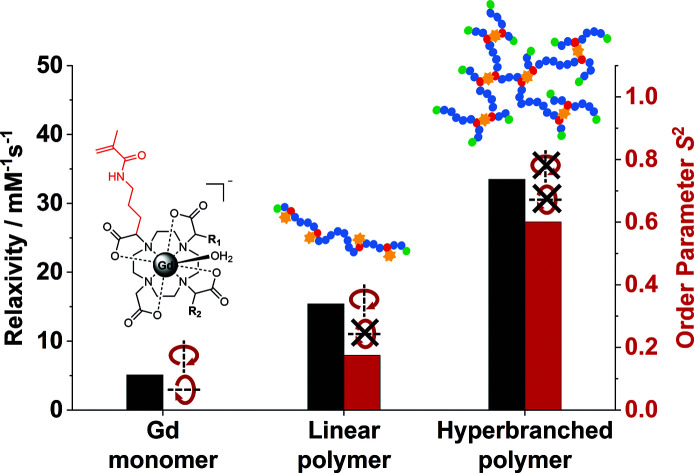

## Introduction

Magnetic Resonance Imaging (MRI) provides 2- and 3-dimensional anatomical information on tissues and organs in a non-invasive manner. MRI displays submillimeter spatial resolution, unlimited penetration depth, and excellent soft tissue contrast imaging, enhancing the diagnostic potential for neurological, cardiovascular and oncological imaging.^1^ However, MRI suffers from intrinsic low sensitivity, and image contrast can be enhanced by using contrast agents (CAs) to increase the relaxation rate of water protons. Most commercial CAs are based on discrete, low molecular weight gadolinium(iii) complexes, such as Gd-DOTA and Gd-DTPA.^2,3^ The ability of discrete Gd(iii) complexes to enhance image contrast is measured by their relaxivity (*r*_1_), which is determined by the number of water molecules coordinated to the metal (*q*), the water exchange lifetime (*τ*_M_) and the rotational correlation time (*τ*_R_). The majority of commercial CAs display relaxivities around 4–5 mM^−1^ s^−1^ (20 MHz, 298 K), far from the theoretical maximum value (*r*_1_ = 100 mM^−1^ s^−1^ for complexes where *q* = 1).^4^

An exciting prospect in MRI CA design is the development of macromolecular systems that possess significantly higher relaxivities. Numerous strategies have been pursued,^5–8^ including the conjugation of one or several Gd(iii) complexes to polymers,^9–12^ dendrimers,^13–18^ micelles^19–23^ or nanoparticles,^24–31^ or *via* the non-covalent association with biomolecules (*e.g.* serum albumin protein)^32,33^ or nano-assembled capsules.^34,35^ Some macromolecular systems have been shown to possess significantly higher relaxivities compared with commercial CAs (up to 40 mM^−1^ s^−1^), attributed predominantly to the slower tumbling of the macromolecules, and the incorporation of several Gd(iii) complexes within a single system.^4,6^

Despite these advances, the challenge still remains to develop high molecular weight CAs wherein the global motion of the macromolecule is effectively coupled to the motion of the paramagnetic centre.^34,36^ One way to approach this challenge involves positioning a single Gd(iii) chelate at the barycentre of the macromolecule (*e.g.* dendritic systems).^17,37^ The rotational correlation time of these high molecular weight Gd(iii) chelates is thus defined by the motion of the macromolecule. Polymeric CAs have been developed which incorporate multiple Ln(iii) chelates, linked *via* a single flexible arm to the polymeric scaffold. However, the use of flexible linkers permits local motion of the paramagnetic centre, limiting the degree of coupling with the more slowly tumbling polymer.^9–12,38^

A further challenge involves the development of new methods to control the macromolecular size, structure and shape, as this could lead to well-defined second spheres of hydration, whilst allowing fast-inner sphere water exchange. The majority of polymeric CAs are prepared by classical conjugation of appropriate ligands to a polymer bearing reactive pendant groups (*e.g.* maleimide group, ester activated monomers). Typically, a protected ligand is covalently attached to a synthesized polymer and the resulting conjugated ligand is deprotected, followed by complexation with Gd(iii).^9,10,39^ In related work, Sherry and co-workers have presented a strategy for polymeric PARACEST agents in which non-metal containing ligands are directly incorporated by the free radical polymerisation of acrylamide functionalised ligands, followed by complexation with Eu(iii).^38^ However, this approach has limitations, because it is difficult to quantify both the extent of ligand conjugation and the degree of lanthanide(iii) complexation. Furthermore, the removal of residual lanthanide(iii) ions can be challenging, but is crucial if such macromolecules are to be considered for biomedical or clinical applications.

**Fig. 1:**
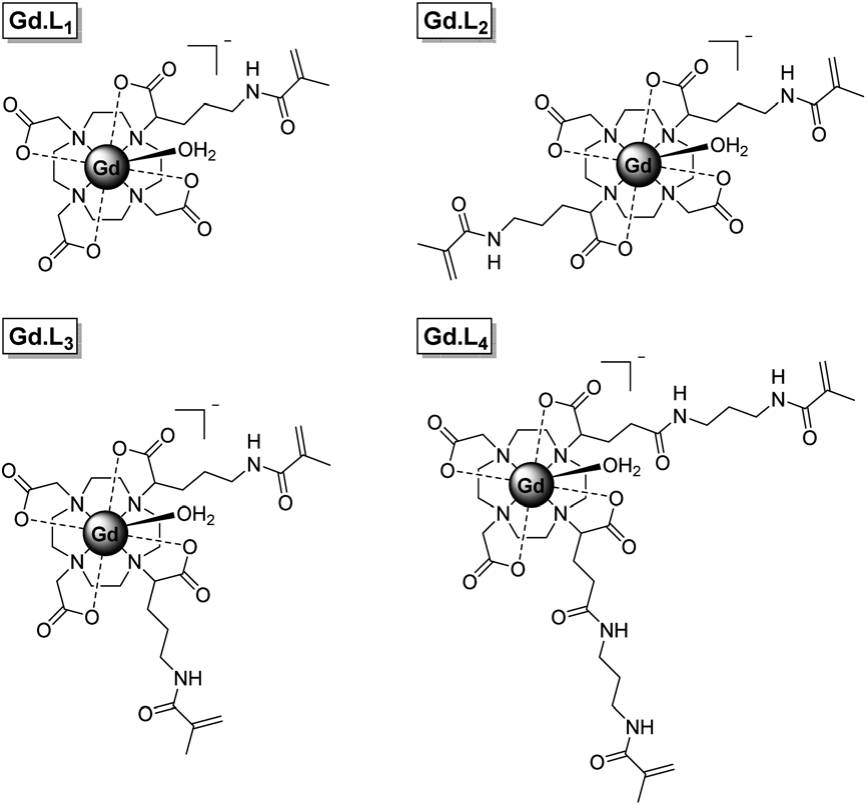
Structures of complexes **Gd·L1–4** developed in this study.

Herein we present a new synthetic approach to macromolecular MRI CAs, involving the single-step incorporation of kinetically stable, monomeric Gd(iii) complexes within well-defined macromolecular CAs. We have synthesized four DOTA-like Gd(iii) complexes (**Gd·L1–4**, Fig. 1), bearing one or two pendant methacrylamide arms, which serve as monomers and crosslinkers respectively, for the synthesis of linear and hyperbranched polymers using reversible addition fragmentation chain transfer (RAFT) polymerization. **Gd·L1** contains a single methacrylamide arm, capable of forming linear polymers, whereas complexes **Gd·L2–4** contain two methacrylamide arms, serving as crosslinkers to create hyperbranched polymers, in which the motion of the Gd(iii) centre is effectively coupled to the slowly tumbling macromolecule. Complex **Gd·L2** possesses two *trans*-related polymerizable arms, whereas for **Gd·L3** and **Gd·L4** the polymerizable arms are in *cis*-geometry, with **Gd·L3** possessing shorter arms. The impact of these structural and geometric modifications on the relaxivity and tumbling motion of the resulting polymers was evaluated. Each complex possesses a DOTA-like core, to confer maximal thermodynamic and kinetic stability.^3,40^ Moreover, **Gd·L1–4** possess a single coordinated water molecule (*q* = 1) and are negatively charged overall, thereby allowing relatively fast water exchange to overcome the limitations of some previously reported macromolecular CAs.^7,8^

## Results and discussion

### Synthesis of monomeric and crosslinker complexes **Gd·L1–4**

**Scheme 1:**
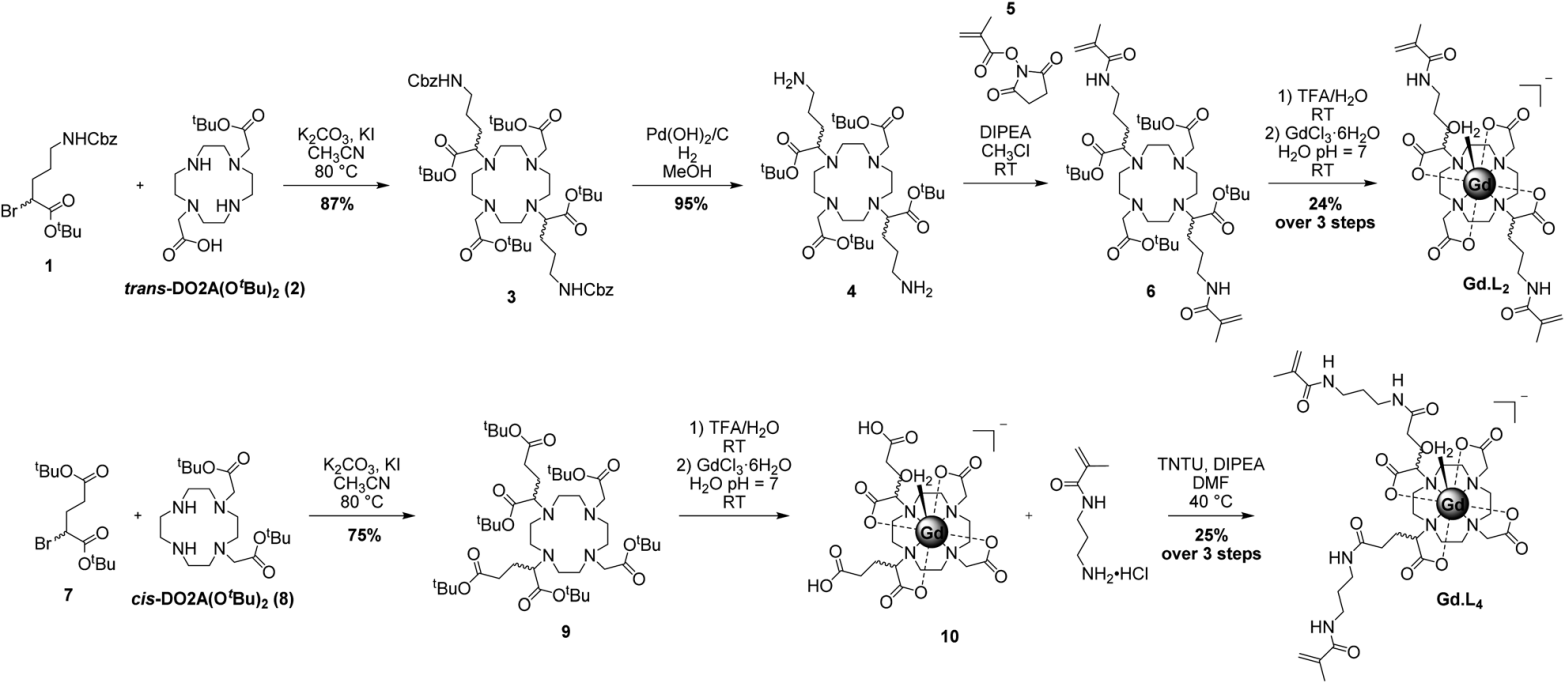
Representative synthesis of crosslinker complexes **Gd·L2** and **Gd·L4**.

The syntheses of the monomeric and crosslinker complexes **Gd·L1–4** were optimized to give sufficient quantities of each complex (approximately 500 mg) to allow optimization of the polymerization reactions. Representative syntheses of **Gd·L2** and **Gd·L4** are presented in Scheme 1 and full details of the synthesis of **Gd·L1–4** can be found in the ESI (Schemes S1–S4†). **Gd·L1–4** were synthesized from *tert*-butyl protected derivatives of 1,4,7,10-tetraazacyclododecane (cyclen), including DO3A(O^*t*^Bu)_3_ for **Gd·L1**, *trans*-DO2A(O^*t*^Bu)_2_ (**2**) for **Gd·L2**, and *cis*-DO2A(O^*t*^Bu)_2_ (**3**) for **Gd·L3–4**. Synthesis of the required *tert*-butyl protected cyclen derivatives was adapted from the literature and full details are provided in the ESI (Section 2).^41–43^

For complexes **Gd·L1–3**, mono or bis-alkylation of the macrocyclic free amines with α-bromoester **1**, prepared from diazotization and bromination of Cbz-protected l-ornithine (Scheme S2†), led to formation of the fully protected macrocyclic ligands (*e.g.***3** for **Gd·L2**, Scheme 1). The yields of the alkylations were significantly improved by the addition of potassium iodide to the reaction mixture (K_2_CO_3_/acetonitrile), allowing iodide/bromide exchange.^44^ It is possible that partial racemisation of the alkylating agent **1** or racemisation during the alkylation reaction occurred,^45^ leading to the formation of the protected ligands as a mixture of stereoisomers. The methacrylamide arms were introduced by Cbz deprotection of the ornithine sidechains to give the bis-amine (*e.g.* macrocycle **4**), followed by coupling with *N*-hydroxysuccinimide methacrylate ester **5**. Next, the *tert*-butyl esters were deprotected using trifluoroacetic acid, followed by the addition of GdCl_3_ in water at pH 7, to afford the water soluble Gd(iii) complexes **Gd·L1–3** after purification by preparative reverse-phase HPLC (Fig. S1–S3†). The Gd(iii) complexes of a given isomer of ligand **L1–3** will have further elements of chirality arising from the sign and torsion angles of the cyclen NCCN chelate rings, and the NCCO chelates defining the helicity of the pendant arms.^3,36^ As such, the Gd(iii) complexes will exist as a mixture of stereoisomers in solution, which may interconvert by either cyclen ring inversion or arm rotation. The separation of stereoisomers was not attempted in this work.

**Fig. 2:**
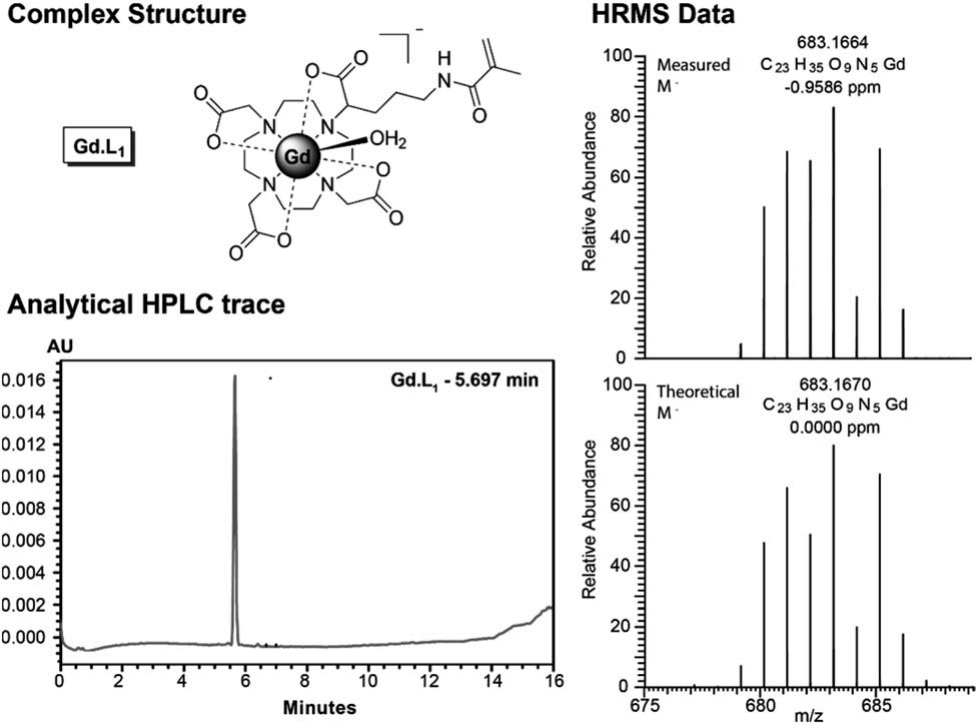
(Left) Analytical reverse-phase HPLC trace of **Gd·L1** (gradient 0–50% acetonitrile in 25 mM NH_4_HCO_3_ over 10 minutes); (Right) High resolution ESI mass spectrum of the molecular ion [**Gd·L1**]^−^, showing excellent agreement with the calculated isotopic distribution.

The synthesis of **Gd·L4** involved initial bis-alkylation of *cis*-DO_2_A(O^*t*^Bu)_2_ (**8**) with α-bromoester **7**, prepared from l-glutamic acid (Scheme S2†) to give protected ligand **9**. Again, it is possible that partial racemisation of α-bromoester **7** occurred, or racemisation during the alkylation reaction, resulting in a mixture of stereoisomers of protected ligand **9**. Subsequent deprotection of the *tert*-butyl esters of **9** using trifluoroacetic acid, followed by the addition of slight excess of GdCl_3_ in water at pH 7, gave the precursor Gd(iii) complex **10**. Finally, the methacrylamide groups were introduced *via* coupling the terminal carboxylic acids of **10** to *N*-(3-aminopropyl)-methacrylamide, using the coupling reagent TNTU (2-(5-norborene-2,3-dicarboximido)-1,1,3,3-tetramethyl-uronium tetrafluoroborate), to give the water soluble complex **Gd·L4** after purification by reverse-phase HPLC. Analysis of the purified complexes **Gd·L1–4** by analytical reverse-phase HPLC revealed a single peak in each case, and high-resolution mass spectral data confirmed formation of the desired complexes (Fig. 2 and S1–S4†). A major signal corresponding to the negatively charged molecular ion, [M]^−^, was observed in each case, and the isotopic distribution was in excellent agreement with the theoretical data.

### Polymerization of monomeric and crosslinker complexes **Gd·L1–4**

RAFT polymerization was utilized to generate copolymers incorporating the monomeric and crosslinker complexes **Gd·L1–4**. RAFT enables access to reproducible polymers with low dispersity and control over the chain length, molecular weight and polymer architecture.^46,47^ Additionally, RAFT can be used in a wide range of conditions, including different temperature, solvents, co-solvents, various additives, and with different monomers (*e.g.* acrylates, methacrylates, acrylamides, styrenes, vinyl esters and vinyl amides).^46,48,49^ Importantly, it has been shown that RAFT is suitable for the polymerization of charged monomers,^50^ hence we postulated that direct polymerization of the Gd(iii) complexes would be possible. Before attempting polymerization with complexes **Gd·L1–4**, we verified that RAFT polymerization of *N*-acryloylmorpholine (NAM) was possible in the presence of the negatively charged Gd(iii) complex, Gd-DOTA. NAM was chosen as the monomer because P(NAM) displays several desirable properties for MRI applications, including good bio-compatibility, very low toxicity, remarkable stealth properties, and prolonged blood residence time.^51,52^ Pleasingly, RAFT polymerization of NAM in the presence of Gd·DOTA was well controlled, with the homopolymer displaying low dispersity and close to target molecular weight (Table 1, entry 1).

**Table 1:** Monomer conversions and SEC data of linear P(NAM-*r*-**Gd·L1**) copolymers

Entry	*N* ^NAM^_0,Chain_ b	*N* ^**Gd·L1**^_0,Chain_ b	NAM^c^ conv., %	Expected *M*_n_^d^, g mol^−1^	*M*_n_ SEC, g mol^−1^	*Ð*	[Gd]^g^, μg L^−1^	*N* ^Gd^_Chain_ h
1^a^	100	0 + 4 Gd·DOTA	97.6	14 181	13 900^e^	1.08^e^	0	0
2	99.1	0.9	98.2	15 632	11 800^e^	1.18^e^	7308	0.37
3	98.1	1.9	96.3	15 894	10 900^e^	1.14^e^	14 680	1.2
4	97.2	2.8	97.5	16 619	10 200^e^	1.12^e^	16 680	1.37
5	96.2	3.8	96.2	16 946	7,600^f^	1.19^f^	18 260	1.59
6	95.5	4.5	98.3	17 863	9,100^f^	1.22^f^	19 560	0.53
7	91.4	8.6	98.1	20 532	12.100^f^	1.20^f^	57 840	2.98
8	83.7	16.7	N/D^i^	25 335	14 200^f^	1.25^f^	62 680	8.02

a RAFT polymerization in the presence of Gd-DOTA.

b Theoretical number of NAM and **Gd·L1** units per chain depending of the initial polymerization reaction composition.

c Conversion determined by ^1^H NMR spectroscopy.

d Expected *M*_n_ = ([NAM]_0_*M*_NAM_Conv_NAM_ + [**Gd·L1**]_0_*M***Gd·L1**Conv**Gd·L1**)/[CTA]_0_ + *M*_CTA_, with Conv**Gd·L1** = 1.

e Obtained by SEC analysis (CH_3_Cl/triethylamine 98 : 2 v/v, RID detectors).

f Obtained by SEC analysis (H_2_O/MeOH 80/20 v/v with 0.1 M NaNO_3_, RID detector).

g Gd(iii) concentration determined by ICP-MS based on mass spectral signal of ^157^Gd isotope.

h *N* ^Gd^Chain = number of Gd(iii) ions per polymer chain, estimated from ICP-MS data (ESI, Section 2, Table S2)_._

i Not determined due to the high content of **Gd·L1**.

**Scheme 2:**
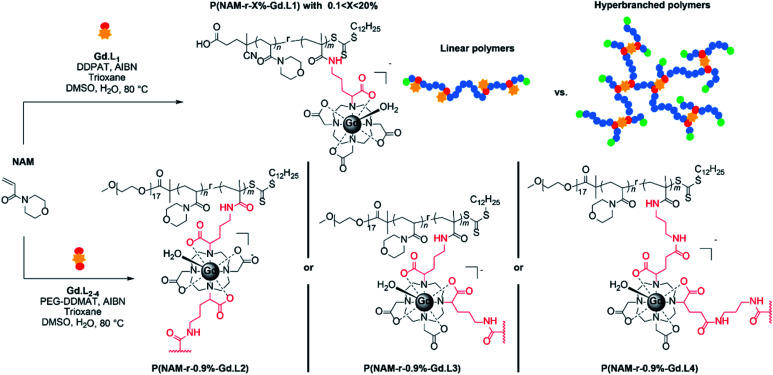
Synthesis of linear (DP 100 and *m* between 1 to 17) and crosslinked (DP 100 and *m* = 0.9) polymeric MRI CAs using NAM and **Gd·L1–4**_._

**Fig. 3:**
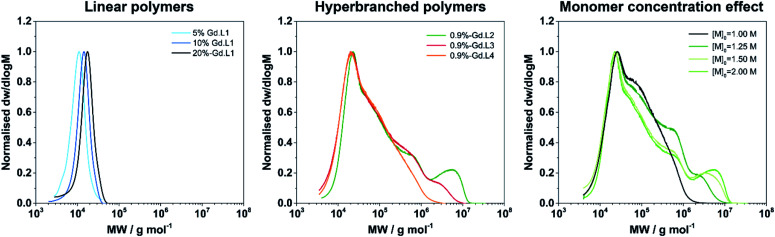
SEC molecular weight (MW) distribution of selected linear and hyperbranched copolymers demonstrating: (left) low *Ð* of linear copolymers and increasing MW as the ratio of **Gd·L1** : NAM increases; (centre) high MW shoulder and broad distribution of MW when a crosslinker is introduced; (right) increase in high MW content as initial concentration of monomer is increased.

Next, the synthesis of linear P(NAM-*r*-**Gd·L1**) copolymers was investigated using different molar proportions of **Gd·L1**, ranging from 1 to nearly 17 mol%. The polymerizations were conducted in a mixture of DMSO/water (80 : 20) at 80 °C (Scheme 2), and the polymers were purified by dialysis through a semi-permeable membrane against distilled water (15 MΩ cm^−1^). Successful synthesis of the target linear P(NAM-*r*-**Gd·L1**) copolymers was confirmed by SEC analysis (Fig. 3 and S7†): polymers with number average molecular weights (*M*_n_) between 7600 and 14 200 g mol^−1^ were formed with low dispersity (*Ð*) values, ranging from 1.08 to 1.25 (Table 1, entries 2–8). Due to the charged nature of the Gd(iii) complex and the difference between the PNAM and the standard used (PS), the polymers displayed lower molecular weights than the theoretical values. Standard ^1^H NMR analysis to confirm *M*_n_ for the polymers was not possible due to severe line broadening imposed by the paramagnetic Gd(iii) ion; however, polymerization of NAM in the presence of free Gd-DOTA resulted in polymers with the expected *M*_n_ by ^1^H NMR spectroscopy.

Analysis of the bulk magnetic susceptibility (BMS) shift of each sample and ICP-MS analysis of the copolymers after dialysis enabled an approximation of the number of Gd(iii) ions per polymer chain (Tables 1, S2 and S3†). As expected, the number of **Gd·L1** units incorporated into the polymer increased with increasing molar ratio of **Gd·L1**/NAM monomers used for the polymerization. In the highest case, eight **Gd·L1** units were incorporated per polymer chain (Table 1, entry 8).

Having demonstrated that linear copolymers can be prepared in a controlled manner using the monomers **Gd·L1** and NAM, we turned our attention to the crosslinker complexes **Gd·L2–4**. In order to prevent gelation from occurring and based on some of our previous work and that of Flynn *et al.*,^50,53^ the crosslinked polymers were obtained by fixing the concentration ratio of the crosslinker (**Gd·L2–4**) relative to the chain transfer agent (CTA), such that [**Gd·L2–4**]/[CTA] = 0.9. Initially, a range of water soluble hyperbranched polymers were synthesized by RAFT polymerization of NAM and the crosslinker **Gd·L2** at different initial concentrations (1–2 M) in DMSO, using poly(ethylene glycol) methyl ether 2-(dodecylthiocarbono-thioylthio)-2-methyl-propionate as the CTA (Table 2, entries 1–4). For each reaction, SEC analysis of the molecular weight distribution indicated the formation of hyperbranched polymers, evident from the high molecular weight shoulder (Fig. 3, centre). The broad molecular weight distribution was reflected in the high dispersities, *Ð,* ranging between 4.2 and 19.9 (Table 2), consistent with crosslinking of the growing polymer chains during RAFT polymerization. Further evidence for crosslinking of **Gd·L2** was given by analysis of the BMS shift and ICP-MS analysis (Tables 2 and S3†), which confirmed an increasing number of crosslinked polymer chains at higher initial monomer concentration. For example, when [NAM]_0_ was 1.00 M, the average number of crosslinker **Gd·L2** units was estimated to be 7.0 per hyperbranched polymer chain, whereas this increased to approximately 34 units per chain when [NAM]_0_ was increased to 2.00 M. A higher initial monomer concentration led to higher molecular weight and dispersity, with values up to *M*_n_ = 744 000 g mol^−1^ and *Ð* = 19.9 when [NAM]_0_ = 2.00 M. However, polymerization conducted at higher initial monomer concentration, such that [**Gd·L2**]/[CTA] > 1, consistently led to the formation of gels (Table 2, entry 5). This is in accordance with the work of Perrier and co-workers, who showed that EGDMA/CTA ratios of over 1 led to gelation in a RAFT system.^54^

**Table 2:** Conditions, monomer conversions and SEC data of hyperbranched copolymers P(NAM-*r*-**Gd·L2–4**), prepared by RAFT polymerization of NAM and **Gd·L2–4**

Entry	Complex	**Gd·L2–4** equiv.	[NAM]_0_ , mol L^−1^	Expected *M*_n_^a^, g mol^−1^	NAM^b^ conv., %	*M*_n_ SEC^c^, g mol^−1^	*M*_w_ SEC^c^, g mol^−1^	*Ð* c	[Gd]^d^, μg L^−1^	*N* ^Gd^_Chain_ d
1	**Gd·L2**	0.9	1.00	15 817	99.1	33 200	138 600	4.2	7128	7.0
2	**Gd·L2**	0.9	1.25	15 817	99.7	38 900	378 600	9.7	4920	21.9
3	**Gd·L2**	0.9	1.50	15 817	98.8	30 800	592 700	19.2	6276	30.5
4	**Gd·L2**	0.9	2.00	15 817	99.6	37 400	744 700	19.9	6008	33.6
5	**Gd·L2**	>1.0	2.00	15 884	>95	Gel	Gel	Gel	N/D	N/D
6	**Gd·L3**	0.9	2.00	15 817	100	26 600	302 800	11.4	N/D	N/D
7	**Gd·L4**	0.9	2.00	16 945	99.6	24 800	119 900	4.8	N/D	N/D

a Expected *M*_n_ = (DP × *n*/100 × *M*_NAM_) + (DP × *m*/100 × *M***Gd·LX**) + *M*_CTA_, with Conv**Gd·L** = 1.

b Determined by ^1^H NMR spectroscopy.

c Determined by SEC (DMF with 5 mM NH_4_BF_4_, RID/UV/LS detector).

d Estimated number of Gd(iii) ions per polymer chain, determined by ICP-MS (ESI, Table S3).

The optimal conditions found for the synthesis of the hyperbranched P(NAM-*r*-**Gd·L2**) polymers were [NAM]_0_ = 2.00 M, [**Gd·L2**]/[CTA] = 0.9 (entry 4). These parameters were applied to the synthesis of hyperbranched polymers using crosslinkers **Gd·L3** and **Gd·L4**, bearing *cis*-related polymerizable arms (entries 6 and 7). Hyperbranched polymers P(NAM-*r*-**Gd·L3**) and P(NAM-*r*-**Gd·L4**) were successfully formed. Notably, they displayed lower molecular weights and dispersities compared with those obtained using **Gd·L2** under similar conditions ([NAM]_0_ = 2.00 M, [**Gd**]/[CTA] = 0.9), suggesting that these polymerizations could be achieved at higher initial monomer concentration (>2.00 M).

**Fig. 4:**
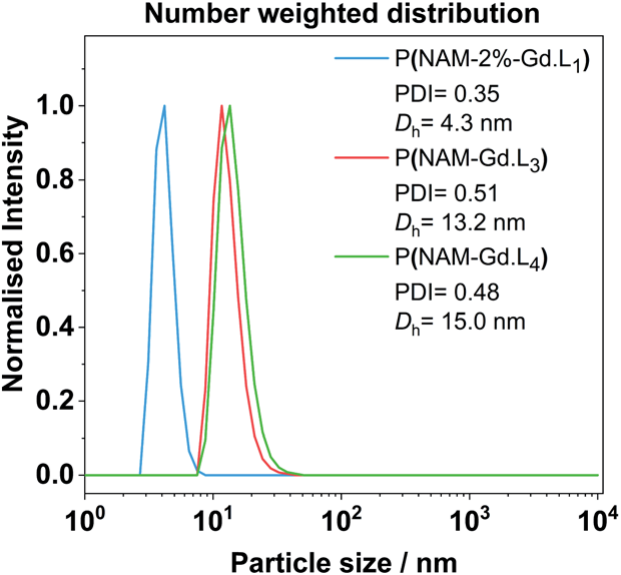
Representative examples of DLS number weighted particle size distribution of linear and hyperbranched polymeric CAs obtained in deionized water at 25 °C after filtration with a syringe filter (220 nm cut-off). Associated correlograms and detailed DLS results are presented in the ESI (Fig. S8 and Table S4†).

The average hydrodynamic diameter *D*_h_ and dispersity of representative examples of linear and hyperbranched polymeric CAs were estimated by diffraction light scattering (DLS) measurements. From the number weighted particle size distribution, the linear polymer P(NAM-2%-**Gd·L1**) has a *D*_h_ of 4.3 ± 0.9 nm. Crosslinked systems with **Gd·L3** or **Gd·L4** display *D*_h_ of 13.2 ± 4.6 nm and 15.1 ± 4.3 nm, respectively (Fig. 4). This indicates that the polymers exist primarily as unimers in solution. The sizes of the hyperbranched polymers are comparable to previously reported hydrophilic and charged hyperbranched polymers (*D*_h_ between 10 to 20 nm for MW between 100 to 500 kDa).^50^ The Gd(iii) monomers are charged and therefore hydrophilic, and as expected, do not direct the assembly of these polymers into higher order structures.

**Fig. 5:**
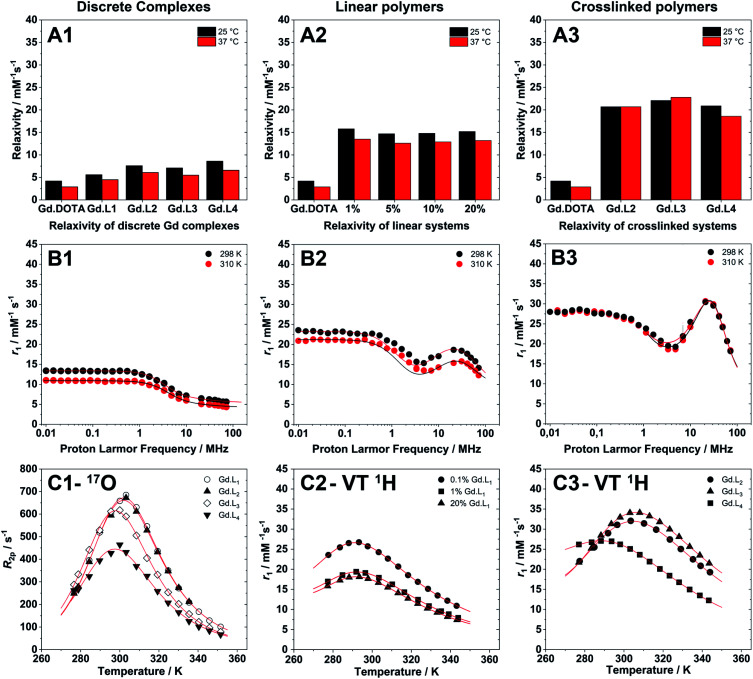
(A1–A3) Relaxivity measurements of discrete complexes, linear and crosslinked polymers, synthesized from **Gd·L1–4**, at 298 K and 310 K, pH 7.4 and 60 MHz (Stelar FFC relaxometer). (B1–B3) Representative NMRD profiles of (B1) a discrete complex (**Gd·L1**), (B2) a linear polymer (P(NAM-1%-**Gd·L1**)), and (B3) a crosslinked system (P(NAM-**Gd·L2**)). (C1) Temperature dependence transversal relaxation from ^17^O NMR, and (C2 and C3) VT relaxivity profiles (VT ^1^H) used to accurately estimate water residence time for macromolecular systems at a fixed magnetic field of 20 MHz.

### ^1^H and ^17^O NMR relaxometric studies

The millimolar water proton longitudinal relaxation rates (*r*_1_ = (*R*_1,obs_ − *R*_1,dia_)/[Gd^3+^]) of a Gd(iii)-chelate, both in monomeric or polymeric forms, depends on the magnetic field strength, temperature and on several important structural and dynamic molecular parameters that describe the magnetic coupling between the water protons and the paramagnetic ion. As shown in Table 3, the *r*_1_ values for the discrete complexes **Gd·L1–4** were found to be in the range 4.5–6.6 mM^−1^ s^−1^ at 60 MHz (310 K, pH 7.4), each higher than that measured for Gd-DOTA under the same experimental conditions (2.9 mM^−1^ s^−1^).^55^ This increase in relaxivity is consistent with the slightly higher molecular weights of **Gd·L1–4** relative to Gd-DOTA, and hence the increase in the rotational correlation time, *τ*_R_.^56^

**Table 3:** Relaxivity and best fitting parameters obtained for the fitting of discrete complexes NMRD profiles at 298 K and ^17^O NMR data (11.75 T)^a^

Parameters	**Gd·L1**	**Gd·L2**	**Gd·L3**	**Gd·L4**
^298^ *r*_1_^20MHz^/mM^−1^ s^−1^	6.5 ± 0.1	8.4 ± 0.1	7.6 ± 0.1	9.1 ± 0.1
^310^ *r*_1_^20MHz^/mM^−1^ s^−1^	5.1 ± 0.1	6.6 ± 0.1	5.9 ± 0.1	7.1 ± 0.1
^298^ *r*_1_^60MHz^/mM^−1^ s^−1^	5.6 ± 0.1	7.6 ± 0.1	7.1 ± 0.1	8.6 ± 0.1
^310^ *r*_1_^60MHz^/mM^−1^ s^−1^	4.5 ± 0.1	6.1 ± 0.1	5.5 ± 0.1	6.6 ± 0.1
*Δ* ^2^/10^18^ s^−2^	9.4 ± 0.1	8.1 ± 0.1	8.2 ± 0.1	7.0 ± 0.1
^298^ *τ*_V_/ps	32.5 ± 0.3	48.8 ± 0.4	29.6 ± 0.4	35.1 ± 0.4
^298^ *τ*_R_/ps	106 ± 1	161 ± 1	142 ± 1	187 ± 1
^298^ *τ*_M_/ns	154 ± 2	166 ± 2	125 ± 1	119 ± 3
Δ*H*^#^_m_/kJ mol^−1^^b^	43.8 ± 0.4	43.1 ± 0.4	43.5 ± 0.3	39 ± 1

a To fit ^1^H NMRD data at 298 K, the following parameters were fixed in the fitting procedure: *q* = 1, *r*_Gd-H_ = 3.0 Å, *a*_Gd-H_ = 4 Å, ^298^*D*_Gd-H_ = 2.25 × 10^−5^ cm^2^ s^−1^.

b From the fitting of the ^17^O NMR data, with the fixed value *E*_V_ = 1 kJ mol^−1^ and *E*_R_ = 20 kJ mol^−1^.

Upon copolymerization of **Gd·L1** with NAM by RAFT, the resulting linear polymers P(NAM-*r*-**Gd·L1**) possessed significantly higher relaxivities (Table 4) in the range 12.6–13.5 mM^−1^ s^−1^ at 60 MHz, and 14.3–15.4 mM^−1^ s^−1^ at 20 MHz (310 K, pH 7.4), up to *ca.* 5 times higher than Gd-DOTA. These relaxivity values are similar to those obtained previously by Davis, Boyer and coworkers,^10,57^ for both discrete core crosslinked star polymers and hyperbranched polymers, each containing Gd(iii) chelates attached *via* a single pendant arm.

**Table 4:** Relaxivity and best fitting parameters obtained for linear and hyperbranched polymer NMRD profiles at 298 K^a^

Parameters	Linear polymers P(NAM-*r-X*% **Gd·L1**)				Hyperbranched polymers P(NAM-*r*-0.9% **Gd·L2–4**)		
	1%	5%	9%	17%	**Gd·L2**	**Gd·L3**	**Gd·L4**
^298^ *r*_1_^20MHz^/mM^−1^ s^−1^	18.6 ± 0.2	17.0 ± 0.2	17.1 ± 0.2	17.6 ± 0.2	30.4 ± 0.4	32.7 ± 0.5	26.0 ± 0.4
^310^ *r*_1_^20MHz^/mM^−1^ s^−1^	15.4 ± 0.2	14.3 ± 0.1	14.4 ± 0.2	15.0 ± 0.1	30.7 ± 0.4	33.5 ± 0.5	23.0 ± 0.4
^298^ *r*_1_^60MHz^/mM^−1^ s^−1^	15.8 ± 0.2	14.8 ± 0.1	14.8 ± 0.1	15.2 ± 0.1	20.7 ± 0.3	22.1 ± 0.2	20.9 ± 0.2
^310^ *r*_1_^60MHz^/mM^−1^ s^−1^	13.5 ± 0.2	12.6 ± 0.1	12.9 ± 0.1	13.2 ± 0.1	20.7 ± 0.3	22.8 ± 0.2	18.6 ± 0.2
*Δ* ^2^/10^18^ s^−2^	4.37 ± 0.06	5.94 ± 0.07	5.61 ± 0.04	5.31 ± 0.05	6.2 ± 0.2	6.3 ± 0.4	5.7 ± 0.3
^298^ *τ*_V_/ps	42.1 ± 0.6	38.1 ± 0.5	38.2 ± 0.3	41.8 ± 0.4	16.1 ± 1	10.4 ± 0.5	12.6 ± 0.3
^298^ *τ*_RL_/ps	342 ± 6	369 ± 5	313 ± 3	346 ± 4	501 ± 45	418 ± 99	447 ± 34
^298^ *τ*_RG_/ps	2680 ± 90	2870 ± 110	2420 ± 50	2500 ± 60	6366 ± 486	6668 ± 812	3810 ± 256
*S* ^2^	0.175	0.118	0.163	0.159	0.500	0.601	0.403
^298^ *τ*_M_/ns^b^	330 ± 5	330 ± 5	330 ± 3	330 ± 4	326 ± 8	308 ± 12	315 ± 9

a To fit the ^1^H NMRD data at 298 K, the following parameters were fixed: *q* = 1, *r*_Gd-H_ = 3.0 Å, *a*_Gd-H_ = 4 Å, ^298^*D*_Gd-H_ = 2.25 × 10^−5^ cm^2^ s^−1^.

b Estimated from VT NMR relaxivity profiles (Fig. 5) at fixed magnetic field (20 MHz).

**Fig. 6:**
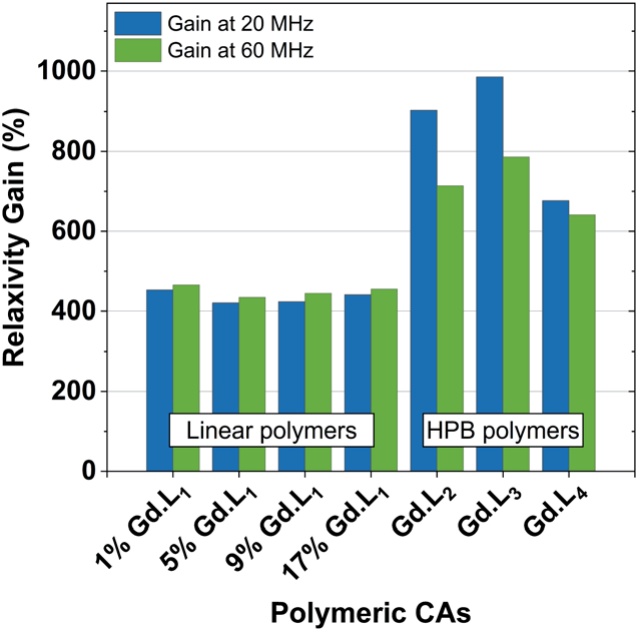
Relaxivity gains observed for our linear or hyperbranched polymers, relative to Gd·DOTA (3.4 and 2.9 mM^−1^ s^−1^ at 20 MHz and 60 MHz, respectively), determined at 310 K at 20 MHz and 60 MHz.

The hyperbranched polymers P(NAM-*r*-**Gd·L2–4**), obtained using crosslinkers **Gd·L2–4**,possessed even higher relaxivities in the range 18.6–22.8 mM^−1^ s^−1^ at 60 MHz, and 23.0–33.5 mM^−1^ s^−1^ at 20 MHz (310 K, pH 7.4) (Table 4). The enhancements in relaxivity of the polymeric CAs relative to the reference agent Gd-DOTA are shown in Fig. 6. Notably, we observe a substantial 9 to 10-fold increase in relaxivity for the crosslinked polymers based on **Gd·L2** and **Gd·L3**, relative to Gd-DOTA at 20 MHz, and a 2-fold increase relative to the linear polymers. Such high gains in relaxivity can be ascribed to the role of **Gd·L2–3** crosslinkers, which reduce the rate of tumbling of the Gd(iii) chelate in the resulting hyperbranched polymers. This limited rotational flexibility leads to much higher relaxivity.^4^ It is also clear from Fig. 6 that the gains in relaxivity for the linear polymers are essentially the same at 20 and 60 MHz, whereas for the crosslinked polymers based on **Gd·L2** and **Gd·L3**, the relaxivity gains are greater when measured at 20 MHz. In contrast, for the crosslinked polymer based on **Gd·L4** the relaxivity enhancement at 20 MHz is less substantial. This can be explained by the faster local tumbling motion of the paramagnetic centre of **Gd·L4**, due to the longer and more flexible crosslinking arms.

Nuclear Magnetic Resonance Dispersion (NMRD) profiles, *i.e.* the variation of relaxivity (*r*_1_) as a function of the applied magnetic field strength, were measured at 298 and 310 K and at pH 7.4 in the proton Larmor frequency range 0.01–70 MHz (0.000234–1.64 T). Representative examples of the NMRD profiles of the monomeric complexes, and of the linear and crosslinked polymers are presented in Fig. 5. The monomeric complexes **Gd·L1–4** displayed profiles typical for fast tumbling, low molecular weight complexes, each characterized by a steady decrease in their relaxivity at low magnetic field (<1 MHz), a drop in relaxivity between 1 MHz to 10 MHz, followed by a second plateau in the high magnetic field region (>10 MHz), governed by the rotational correlation time, *τ*_R_. In the high field region, the relaxivity is similar for the four monomers **Gd·L1–4** as expected, since their similar molecular weight, size and charge results in similar rotational dynamics. Raw data and fitted NMRD profiles are reported in the ESI (Tables S5–S8 and Fig. S9†).

The NMRD profiles of **Gd·L1–4** were fitted according to the established theory of paramagnetic relaxation expressed by the Solomon–Bloembergen–Morgan (SBM) and Freed's equations for the inner- (IS) and outer-sphere (OS) proton relaxation mechanisms, respectively (see ESI, eqn (S1)–(S11)†).^58–60^ Certain parameters were fixed to reasonable values according to previously reported examples:^25,56,61^ a hydration number of *q* = 1 was assumed, and the distance between the Gd(iii) ion and a bound water molecule proton, *r*_GdH_, was set to 3.0 Å, based on crystallographic data for Gd-DOTA. The closest approach of the bulk water molecules, *a*_GdH_, was set to 4.0 Å, and the diffusion coefficient of a water proton away from the Gd(iii) centre was assumed equal to *D*_GdH_ = 2.25 × 10^−5^ cm^2^ s^−1^ at 298 K, or 3.1 × 10^−5^ cm^2^ s^−1^ at 310 K.

To provide better estimates of the rotational correlation times of **Gd·L1–4**, by fitting of the NMRD profiles, the water residence lifetimes (*τ*_M_ = 1/*k*_ex_) were determined by ^17^O NMR relaxometry. Thus, the temperature dependence of the transverse relaxation rate (*R*_2_) and chemical shifts (Δ*ω*_r_) were determined at 11.75 T at neutral pH using relatively concentrated solutions of **Gd·L1–4** (Fig. 5 and S10, Table S9–S12†) and the profiles were fitted according to the Swift–Connick theory for ^17^O relaxation.^62,63^ For complexes **Gd·L1–4** the *τ*_M_ values were similar (*τ*_M_ = 119–166 ns) and in line with the values determined for similar Gd-DOTAGA (DOTAGA = 2-(4,7,10-tris-carboxymethyl-1,4,7,10-tetraazacyclododecan-1-yl)pentanoic acid) derivatives under the same conditions.^25^ However, it appears that the complexes **Gd·L3** and **Gd·L4**, bearing *cis*-methacrylamide arms, exhibit slightly faster water exchange.

The NMRD profiles obtained for the linear and crosslinked polymers revealed a significant increase in relaxivity over the entire proton Larmor frequency range (Fig. 5), compared with monomeric complexes **Gd·L1–4** (raw data and fitted NMRD profiles are reported in Tables S13–S19 and Fig. S11 and S12†). The profile shapes were also distinctly different, indicating successful incorporation of the monomeric complexes into higher molecular weight macromolecules. The profiles of the linear copolymers P(NAM-*r*-**Gd·L1**) displayed a broad relaxivity peak in the Larmor frequency range of 10–60 MHz, whereas in the case of the hyperbranched (crosslinked) polymers, the relaxivity peak was sharper in the 20–50 MHz region, indicating slower tumbling of the crosslinked polymers compared with the linear polymers.

To obtain more accurate fitting of the NMRD profiles of the polymeric systems, *τ*_M_ values were estimated by fitting of the variable temperature ^1^H NMR profiles at 20 MHz for the linear polymers containing between 1–17 mol% **Gd·L1** theoretically, and for all hyperbranched polymers (Table S22–S24†). In fact, the relatively low concentration of Gd(iii) in the polymeric systems prevented the acquisition of ^17^O NMR data, which typically require Gd(iii) concentrations in excess of 5 mM. The water exchange rate of the coordinated water molecule of the polymerized Gd(iii) complexes were determined to be two times slower than the corresponding monomeric complexes (*τ*_M_ values between 300–330 ns). Slower water exchange kinetics has been observed previously for other macromolecular Gd(iii) systems,^25,27^ and in the current study this may be tentatively ascribed to weak non-covalent interactions between the Gd(iii) chelate and polymer backbone. Alternatively, a reduced rate of water diffusion through the polymer could also contribute to the slower water exchange rate.^10,64^ For linear polymers with 5 and 9 mol% of **Gd·L1**, the water residence lifetime was assumed to be 330 ns, since these polymers have similar molecular weights, dimensions and relaxivities to those containing 1 and 17 mol% **Gd·L1**.

The NMRD profiles for the linear and crosslinked polymers were fitted based on SBM theory and modified with the Lipari–Szabo approach for the description of the rotational dynamics of Gd(iii) chelates covalently linked to macromolecules (see equations in Section 5).^65,66^ In particular, the contributions of the fast local tumbling motion of the Gd-chelate (*τ*_RL_) were separated from the slower global tumbling of the macromolecule (*τ*_RG_). *τ*_RL_ and *τ*_RG_ are associated with the order parameter, *S*^2^, which describes the level of interconnectivity between the local and the global motions (*i.e.*, if *S*^2^ = 0 the motions are independent, if *S*^2^ = 1 the motions are fully linked).

The NMRD profiles of the linear polymers were fitted over the entire range of magnetic fields investigated (0.01 to 70 MHz, Fig. 5-B2 and S11†), whereas for the hyperbranched systems, the profiles were better fitted using only the high field data, *i.e.* above 1 MHz (Fig. 5-B3 and S12†),‡‡NMRD profiles for the hyperbranched systems were also fitted over the entire Larmor frequency range (0.01–100 MHz) and are provided in the ESI (Fig. S13 and Table S21). The resulting NMRD parameters are very similar to those obtained from the high field fitting, presented in Fig. 5. as commonly performed for large macromolecular MRI CAs.^1^

Compared with the discrete complexes **Gd·L1–4**, the linear polymers displayed slower local (*τ*_RL_ greater than 2-fold longer) and slow global (*τ*_RG_ greater than 20-fold longer) reorientation correlation times (Table 4). This can be ascribed to the incorporation of **Gd·L1** into polymer chains *via* the pendant arm of the macrocyclic ligand. The order parameter, *S*^2^, obtained for the linear polymers ranged between 0.12 and 0.18, which is reasonable for macromolecules containing Gd-chelates conjugated *via* a single flexible linker, which allows relatively fast local tumbling. The hyperbranched polymers containing **Gd·L2–4** showed a large increase in relaxivity (18.6–22.8 mM^−1^ s^−1^ at 60 MHz), primarily attributed to the slower tumbling of the crosslinked Gd(iii) complexes. In fact, both *τ*_RL_ and *τ*_RG_ increased relative to the linear polymers (Table 4), and the order parameter *S*^2^, was also much higher than for the linear polymers (*S*^2^ ≈ 0.60 for P(NAM) containing 0.9% **Gd·L3**), consistent with a more restricted motion of the Gd(iii) chelate within the crosslinked systems.

Further inspection of the relaxivity data revealed that for the crosslinked polymers prepared from **Gd·L2** and **Gd·L3**, there is very little difference in relaxivity at 298 K and 310 K (Fig. 5-A3). This cannot be explained by differences in water exchange rate, since this parameter is very similar for the different polymers synthesised (Table 4). Rather, this can be attributed to more effective coupling of the local and global tumbling motion (*S*^2^ up to 0.60) in the crosslinked systems, hence we do not lose any relaxivity gains at higher temperature. In the case of the crosslinked polymer synthesised from **Gd·L4**, the local and global motion is less effectively coupled (*S*^2^ = 0.40), and consequently the relaxivity at 310 K is approximately 11% lower than at 298 K. A similar decrease in relaxivity is observed for the linear polymers at 310 K, where the motion of the Gd(iii) centre is less efficiently coupled to the more slowly tumbling polymer (*S*^2^ = 0.12–0.18).

### Comparison with previous macromolecular CAs

A comparison of the relaxivities and NMRD parameters obtained in this work with previously reported linear and crosslinked systems (acquired at the same magnetic field, temperature and pH) is given in Table 5. Our linear polymers display similar or slightly higher relaxivities (12.6–13.5 mM^−1^ s^−1^ at 60 MHz) and similar NMRD parameters to comparable macromolecular or nanoscale systems (11–13 mM^−1^ s^−1^), involving Gd(iii) complexes attached *via* single flexible linker.^25,27^

**Table 5:** Comparison of NMRD parameters obtained in this work with previously reported linear and crosslinked systems^a^

	Comparison with linear systems			Comparison with hyperbranched systems		
	This work	Ref. 25	Ref. 27	This work	Ref. 25	Ref. 67
Description	Linear copolymer formed using monomer **Gd·L1**	Self-assembled structures from an amphiphilic Gd complex	Fibril-shaped nanoparticles from block copolymers	Hyperbranched polymer made from crosslinker **Gd·L3**	Self-assembled structures made from a rigid amphiphilic Gd complex	Hyperbranched (dendrimer like) amino-functionalized polyglycerol
Name	P(NAM-*r*-**Gd·L1**)	Gd-DOTAGAC_12_	FMN	P(NAM-*r*-**Gd·L3**)	Gd-DOTA(GAC_12_)_2_	HB-PG-Gd(DOTA-*p*Bn)
^298^ *r*_1_/mM^−1^ s^−1^	15.8	≈ 13–14.5	—	22.1	≈ 27–29	≈ 25
^310^ *r*/mM^−1^ s^−1^	13.5	≈ 11–11.5	≈ 13	22.8	≈ 23–25	—
*Δ* ^2^/10^18^ s^−2^	4.37 ± 0.06	4.9	7	6.3 ± 0.4	5.2	—
^298^ *τ*_V_/ps	42.1 ± 0.6	11	53	10.4 ± 0.5	13	—
^298^ *τ*_RL_/ps	342 ± 6	210	150	418 ± 99	820	530
^298^ *τ*_RG_/ps	2680 ± 90	2900	2800	6668 ± 812	4700	4000
*S* ^2^	0.175	0.14	0.25	0.601	0.7	0.36
^298^ *τ*_M_/ns^b^	330 ± 5	220	350	308 ± 12	297	152

a *r*_1_ values are given at 60 MHz. Relaxivities of literature examples were estimated from NMRD profiles. For ref. 25, a range of relaxivities is given, accounting for differences observed depending on the type of assembly formed (*e.g.* micelles or liposomes).

b Water residence times (*τ*_M_) were estimated from VT NMR relaxivity profiles at fixed magnetic field (20 or 40 MHz).

The hyperbranched polymers containing **Gd·L2–4** showed a large increase in relaxivity (18.6–22.8 mM^−1^ s^−1^ at 60 MHz). Interestingly, the values of the rotational correlation times and of *S*^2^ are comparable to those reported for micellar aggregates obtained by self-assembly of a Gd·DOTAGA_2_ complex bearing two C_12_ aliphatic chains in *cis*-position,^25^ as seen in Table 5. Also in that example, the restricted local motion of the Gd(iii) complex was responsible for a strong relaxivity enhancement with respect to analogous micelles embedding a Gd(iii) complex bearing only one aliphatic chain. Our hyperbranched polymers also displayed similar relaxivities and NMRD parameters to those obtained for hyperbranched dendrimers, conjugated with Gd·DOTA-*p*Bn *via* a single arm (*r*_1_ ≈ 25 mM^−1^ s^−1^, 298 K).^67^

Comparing our hyperbranched polymers at 60 MHz and 310 K (Table 4), the system prepared from crosslinker **Gd·L3**, bearing the shortest pendant arms in a *cis* orientation, displayed a higher relaxivity (22.8 mM^−1^ s^−1^), than systems prepared from the *trans*-oriented crosslinker **Gd·L2** (20.7 mM^−1^ s^−1^) or the *cis*-oriented crosslinker **Gd·L4** with longer arms (18.6 mM^−1^ s^−1^). This indicates that the combination of shorter crosslinker arms in a *cis*-geometry is most ideal for limiting the local motion of the Gd(iii) complex within the hyperbranched macromolecules.

Of the very few reported macromolecular CAs wherein the Gd(iii) complex behaves as a crosslinker,^68–70^ our system is the only example which takes advantages of the crosslinking to efficiently reduce the local rotational tumbling. This leads to higher relaxivity than those previously reported (*e.g.* crosslinked acrylamide nanogels^68^ bearing Gd-DOTA or DTPA like ligands show *r*_1_ = 9.7–17.6 mM^−1^ s^−1^, 60 MHz, 310 K). To the best of our knowledge, only one other crosslinked system displays a slightly higher relaxivity (24.1 mM^−1^ s^−1^ at 60 MHz, 310 K),^69^ which we propose is due to the slower global tumbling of the nanoparticles (average diameter of 65 nm), and faster water exchange (since all the Gd(iii) complexes are on the outside of the nanoparticle), despite exhibiting faster local tumbling.

## Conclusions

We have developed a strategy for the efficient synthesis of high molecular weight macromolecular contrast agents, *via* RAFT polymerization of kinetically stable Gd(iii) monomers and crosslinkers **Gd·L1–4**, each based on a DOTA-like core bearing one or two pendant methacrylamide arms.

Copolymerization of **Gd·L1**, bearing a single methacrylamide arm, with NAM led to the formation of linear polymers with higher relaxivities (12.6–13.5 mM^−1^ s^−1^ at 60 MHz) and slower tumbling compared with the discrete monomeric complexes **Gd·L1–4**. Moreover, hyperbranched polymers prepared *via* the incorporation of crosslinked Gd(iii) chelates **Gd·L2–4** displayed significantly higher relaxivities and slower tumbling compared with the linear polymers. Analysis of the NMRD profiles revealed that the higher relaxivities of the hyperbranched polymers is due to the restricted motion of the crosslinked Gd(iii) chelates, which approximately doubles the local and global reorientation correlation times relative to the linear polymers containing **Gd·L1**. Crucially, the global motion of the hyperbranched polymers was more effectively coupled to the motion of the paramagnetic centre. This is apparent from an increase in the order parameter, *S*^2^, relative to the linear polymers, thus showing that the rotational flexibility was significantly reduced for polymers containing crosslinkers **Gd·L2–4**.

Hyperbranched polymers prepared from **Gd·L3** displayed the highest relaxivity (22.8 mM^−1^ s^−1^ at 60 MHz), suggesting that the combination of shorter polymerizable arms in a *cis*-orientation is optimal for limiting the local motion of the Gd(iii) complex within a hyperbranched polymer. In comparison, hyperbranched polymers prepared from **Gd·L4**, bearing more flexible arms in a *cis*-geometry, or from **Gd·L2** bearing two *trans*-related polymerizable arms, showed lower relaxivities (18.6–20.7 mM^−1^ s^−1^ at 60 MHz). The results obtained herein will guide the design of second generation Gd(iii) monomers possessing three or four polymerizable arms, in order to access macromolecules with even higher relaxivities.

Our synthetic approach to macromolecular CAs combines the simplicity of a single polymerization step (with no post-polymerization modification) and scalability. The monomeric complexes **Gd·L1–4** serve as building blocks for the construction of more complex polymeric MRI CAs possessing responsive or theragnostic properties.^71^ Further, polymeric CAs capable of *in vivo* targeting may be addressed through the copolymerization of **Gd·L1–4** with monomers containing water-soluble sugar moieties or small peptide sequences, which modulate the second sphere of hydration. Work in this regard is ongoing in our laboratories.

## Experimental

### Synthesis of **Gd·L2** and **Gd·L4**

#### *tert*-Butyl (*S*)-5-(((benzyloxy)carbonyl)amino)-2-bromopentanoate (**1**)

A solution of sodium nitrite (2.59 g, 37.6 mmol, 2.00 equiv.) in water (5 mL) was added dropwise over two hours to a cold (−10 °C) stirred solution of (*S*)-2-amino-5-(((benzyloxy)carbonyl)-amino)pentanoic acid (5.00 g, 18.8 mmol, 1.00 equiv.), potassium bromide (8.27 g, 69.6 mmol, 3.70 equiv.) bromohydric acid (9.40 mL, 47.0 mmol, 2.50 equiv., 48% w/w in water) in water (100 mL). The resulting yellow solution was stirred at room temperature overnight. The reaction mixture was then extracted with diethyl ether (3 × 100 mL) and the combined organic phases were washed with brine (100 mL), dried over MgSO_4_, filtered and concentrated under reduced pressure to give a viscous yellow liquid. The crude product was dissolved in *tert*-butyl acetate (60.0 mL) and an aqueous solution of HClO_4_ (70% w/w in H_2_O, 70 μL, 0.81 mmol, 0.05 equiv.) was added and the reaction mixture was stirred at room temperature for 18 hours. Diethyl ether (100 mL) and water (50 mL) were added to the reaction mixture. The organic phase was isolated and washed with a saturated aq. solution of Na_2_CO_3_ until neutral pH is obtained. The organic layer was then dried over MgSO_4_, filtered and concentrated under reduced pressure. The residue was purified by column chromatography (silica gel, Pet. ether/EtOAc, 2 : 1 v/v) to give compound **1** as a colourless oil (3.32 g, 8.60 mmol, 46% over 2 steps). IR (*ν*_max_/cm^−1^, neat): 3341, 2940, 1705, 1528, 1250, 1141, 741, 694. *R*_f_ (Pet. ether/EtOAc v/v 70 : 30): 0.42. ^1^H NMR (400 MHz, CDCl_3_) *δ* (ppm): 7.49–7.28 (m, 5H, CH aromatic), 5.08 (s, 2H, CH_2_Ph), 4.12 (t, ^3^*J* = 7.2 Hz, 1H, CH), 3.22 (app. q, ^3^*J* = 6.7 Hz, 2H, NHC*H*_2_), 2.15–1.80 (m, 2H, C*H*_2_CHBr), 1.75–1.50 (m, 2H, CH_2_C*H*_2_CH_2_), 1.46 (s, 9H, CH_3_). N–H signals are not observed. ^13^C NMR (101 MHz, CDCl_3_) *δ* (ppm): 168.8 (*C*O_2_^*t*^Bu), 156.5 (NH*C*O_2_CH_2_), 136.6 (C^IV^ aromatic), 128.7, 128.3 (CH aromatic), 82.7 (*C*(CH_3_)_3_), 66.9 (CH_2_Ph), 47.2 (CH), 40.3 (NHCH_2_), 32.1 (*C*H_2_CHBr), 27.9 (*C*H_3_ and CH_2_*C*H_2_CH_2_). HRMS (ESI^+^, *m*/*z*) calculated for M = C_17_H_24_^79^BrNO_4_, [M + Na]^+^: 408.0781, found 408.0779.

#### Di-*tert*-butyl-2,2′-(4,10-bis(2-(*tert*-butoxy)-2-oxoethyl)-1,4,7,10-tetraazacyclododecane-1,7-diyl)bis(5-(((benzyloxy)-carbonyl)ami-no)pentanoate) (**3**)

*trans*-DO2A(O^*t*^Bu)_2_ (ref. 43) (**2**) (800 mg, 2.00 mmol, 1.00 equiv.), K_2_CO_3_ (1.66 g, 12.0 mmol, 6.00 equiv.), α-bromoester **1** (2.01 g, 5.20 mmol, 2.60 equiv.), and potassium iodide (663 mg, 4.00 mmol, 2.00 equiv.) were dissolved in anhydrous CH_3_CN (10.0 mL), and the reaction mixture was stirred at 80 °C overnight. Potassium salts were removed by centrifugation, and the supernatant was concentrated under reduced pressure to obtain a yellow oil. The crude material was then purified by column chromatography (silica gel, pure CH_2_Cl_2_ to CH_2_Cl_2_/CH_3_OH, 92 : 8 v/v, with an increment of 2%) to yield compound **3** (1.76 mg, 1.74 mmol, 87%) as a yellow solid. IR (*ν*_max_/cm^−1^, neat): 3254, 2975, 2933, 2837, 1711, 1517, 1227, 1154, 1113. *R*_f_ (CH_2_Cl_2_/CH_3_OH, v/v 90 : 10): 0.32. ^1^H NMR (400 MHz, CDCl_3_) *δ* (ppm): 7.40–7.26 (m, 10H, CH aromatic), 5.06 (s, 4H, CH_2_Ph), 3.42 (d, ^2^*J* = 17.1 Hz, 2H, CO_2_CH_2_N), 3.32 (m, 2H, CH), 3.19 (m, 4H, NHC*H*_2_CH_2_), 3.07 (app. t, *J*_app_ = 13.3 Hz, 2H, CH_2_ cyclen), 2.94 (app. t, *J*_app_ = 13.3 Hz, 2H, CH_2_ cyclen), 2.76 (d, ^2^*J* = 17.1 Hz, 2H, CO_2_CH_2_N), 2.65–2.25 (m, 8H, 4 × CH_2_ cyclen), 2.20 (app. d, *J*_app_ = 13.3 Hz, 2H, CH_2_ cyclen), 2.08 (app.d, *J*_app_ = 13.3 Hz, 2H, CH_2_ cyclen), 1.86 (m, 2H, CH_2_C*H*_2_CH_2_), 1.69 (m, 2H, CH_2_C*H*Br), 1.53–1.65 (m, 4H, overlap CH_2_C*H*Br + CH_2_C*H*_2_CH_2_), 1.53–1.30 (m, 36H, CH_3_). N–H signals are not observed. ^13^C NMR (101 MHz, CDCl_3_) *δ* (ppm): 174.9, 172.9 (*C*O_2_^*t*^Bu), 156.6 (PhCH_2_*C*O_2_), 136.8 (C^VI^ aromatic), 128.5, 128.0 (CH aromatic), 82.1, 81.9 (*C*(CH_3_)_3_), 66.4 (CH_2_Ph), 61.0 (*C*H), 56.0 (N*C*H_2_CO_2_), 52.7, 48.8, 47.3, 44.6 (CH_2_ cyclen), 40.8, 40.7 (NH*C*H_2_CH_2_), 29.8 (NHCH_2_*C*H_2_), 28.3, 28.2, 27.9 (CH_3_), 22.0 (CH*C*H_2_). HRMS (ESI^+^, *m*/*z*) calculated for M = C_54_H_86_N_6_O_12_, [M + Na]^+^: 1033.6196, found 1033.6189.

#### Di-*tert*-butyl-2,2′-(4,10-bis(2-(*tert*-butoxy)-2-oxoethyl)-1,-4,7,10-tetraazacyclododecane-1,7-diyl)bis(5-aminopentanoate) (**4**)

Compound **3** (50.0 mg, 0.49 mmol, 1.00 equiv.) and Pd(OH)_2_ (10.0 mg, 74 μmol, 0.15 equiv.) were dissolved in CH_3_OH (1.50 mL) at room temperature. The reaction mixture was purged with N_2_ and the inert atmosphere was replaced with H_2_. The reaction mixture was stirred overnight at room temperature and the catalyst was removed by filtration through a pad of Celite® and rinsed with CH_3_OH. The resulting solution was filtered through a syringe filter (220 nm cut-off) and the filtrate was concentrated under reduced pressure to give compound **4** (35.0 mg, 0.47 mmol, 95%) as a yellow oil. IR (*ν*_max_/cm^−1^, neat): 3368, 2977, 2932, 2844, 1718, 1577, 1455, 1367, 1227, 1155. ^1^H NMR (400 MHz, CDCl_3_) *δ* (ppm): 3.41 (d, ^2^*J* = 17.3 Hz, 2H, CO_2_CH_2_N), 3.37–3.33 (m, 2H, CH), 3.22 (app. t, *J*_app_ = 13.4 Hz, 2H, CH_2_ cyclen), 2.92 (app. t, *J*_app_ = 13.0 Hz, 2H, CH_2_ cyclen), 2.75 (d, ^2^*J* = 17.3 Hz, 2H, CO_2_CH_2_N), 2.67 (m, 4H, C*H*_2_NH_2_), 2.57–2.31 (m, 8H, 4 × CH_2_ cyclen), 2.26 (app. d, *J*_app_ = 13.6 Hz, 2H, CH_2_ cyclen), 2.09 (app. d, *J*_app_ = 14.0 Hz, 2H, CH_2_ cyclen), 1.90–1.53 (m, 8H, CHC*H*_2_C*H*_2_), 1.50–1.35 (m, 36H, CH_3_). N–H signals are not observed. ^13^C NMR (101 MHz, CDCl_3_) *δ* (ppm): 174.8, 174.0, 173.2, 172.7 (*C*O_2_C(CH_3_)_3_), 83.0, 82.3, 81.8, 81.7 (*C*(CH_3_)_3_), 61.0 (CH), 56.3, 55.9, 55.8 (N*C*H_2_CO_2_), 52.6, 48.7, 47.1, 46.9, 44.5 (*C*H_2_ cyclen), 41.7 (NH_2_CH_2_), 32.9 (CH_2_*C*H_2_CH_2_), 28.1, 27.7, 27.6 (CH_3_), 22.1 (CH*C*H_2_). HRMS (ESI^+^, *m*/*z*) calculated for M = C_38_H_74_N_6_O_8_, [M + H]^+^: 743.5641, found 743.5648.

#### Gadolinium(iii)(2,2′-(4,10-bis(carboxylatomethyl)-1,4,7,10-tetra-azacyclododecane-1,7-diyl)bis(5-methacrylamdop-entanoate)) (**Gd·L2**)

##### Part A – synthesis of di-*tert*-butyl-2,2′-(4,10-bis(2-(*tert*-butoxy)-2-oxoethyl)-1,4,7,10-tetraazacyclododecane-1,7-diyl)bis(5-methacryl-amidopentanoate) (**6**)

Bis-amine **4** (540 mg, 0.73 mmol, 1.00 equiv.) was dissolved in DMF (2.00 mL) at room temperature. DIPEA (506 μL, 2.91 mmol, 4.00 equiv.) and **5** (NHS-methacrylate) (280 mg, 1.53 mmol, 2.10 equiv.) were added. The reaction mixture was stirred at 30 °C overnight under an atmosphere of N_2_. The solvent was evaporated, and the residue was dissolved with CH_2_Cl_2_ (40.0 mL). The organic phase was washed with a mixture of water (20.0 mL) and a saturated solution of NH_4_Cl (2.00 mL), dried over MgSO_4_, filtered and concentrated. Compound **6** was not stable on silica gel and therefore the product was used directly in the next step with no further purification. IR (*ν*_max_/cm^−1^, neat): 3257, 2976, 2929, 2852, 1716, 1656, 1615, 1533, 1227, 1151. HRMS (ESI^+^, *m*/*z*) calculated for M = C_54_H_86_N_6_O_12_, [M + Na]^+^: 901.5985, found 901.5971.

##### Part B – synthesis of 2,2′-(4,10-bis(carboxymethyl)-1,4,7,10-tetraazacyclododecane-1,7-diyl)bis-(5-methacrylamidopentanoic acid) (**6b**)

Ligand **6** (600 mg, 0.68 mmol, 1.00 equiv.) was dissolved in TFA (10.0 mL) and H_2_O (1.00 mL) and was the mixture was stirred for 3 days at room temperature until complete deprotection (LC-MS monitoring). The reaction mixture was concentrated under reduced pressure and residual TFA was removed by successive co-evaporations with CH_2_Cl_2_ (5 × 20 mL). The resulting residue was dissolved in deionised water, centrifuged twice, and the supernatant filtered through a syringe filter (cut-off of 220 nm). The filtrate was lyophilized to afford **6b** (354 mg) as a white solid, which was used in the next step with no further purification. HRMS (ESI^+^, *m*/*z*) calculated for M = C_30_H_50_N_6_O_10_, [M + Na]^+^: 655.3631, found 655.3680.

##### Part C

Deprotected ligand **6b** (354 mg, 0.54 mmol, 1.00 equiv.) and gadolinium(iii)chloride hexahydrate (221 mg, 0.60 mmol, 1.10 equiv.) were stirred in deionized water (10.0 mL) for 24 hours at room temperature. Over the course of the reaction, the pH was adjusted to 7 by addition of aq. HCl or aq. NaOH. The reaction mixture was lyophilized and the resulting solid was redissolved in deionized water, centrifuged twice, and the supernatant was filtered through a syringe filter (220 nm cut-off). The crude product was purified by preparative HPLC (gradient 0–100% acetonitrile in 25 mM NH_4_HCO_3_ over 10 minutes) to give **Gd·L2** (103 mg, 128 μmol, 24% over 3 steps) as a white powder. IR (*ν*_max_/cm^−1^, neat): 3286, 2980, 2866, 1592, 1389. HRMS (ESI^−^, *m*/*z*) calculated for M = C_30_H_46_^158^GdN_6_O_10_, [M]^−^: 808.2511, found 808.2543. HPLC [gradient 0–100% acetonitrile in 25 mM NH_4_HCO_3_ over 10 minutes]: *t*_R_ = 5.591 minutes.

#### Di-*tert*-butyl(*S*)-2-bromopentanedioate (**7**)

##### Part A – (*S*)-2-bromopentanedioic acid

(*S*)-2-bromopentanedioic acid was synthesized according to literature.^25^l-Glutamic acid (9.21 g, 62.6 mmol, 1.00 equiv.) and KBr (22.4 g, 188 mmol, 3.00 equiv.) were dissolved in aq. HBr (1.00 M, 143 mL, 143 mmol, 2.30 equiv.) at room temperature. The reaction mixture was stirred for 5 minutes and cooled to −10 °C. A solution of NaNO_2_ (10.8 g, 157 mmol, 2.50 equiv.) in water (15 mL) was added dropwise over 2 hours. After the addition, the reaction mixture was stirred at room temperature overnight. H_2_SO_4_ (4.00 mL) was added slowly and the resulting aqueous solution was extracted with Et_2_O (3 × 100 mL). The combined organic layers were washed with brine (2 × 75 mL), dried over MgSO_4_, filtered and concentrated under reduced pressure. The crude (*S*)-2-bromopentanedioic acid (4.00 g) was used directly in the next step without further purification.

##### Part B

Aqueous HClO_4_ (70% w/w, 125 μL, 1.42 mmol, 0.07 equiv.) was added to a solution of (*S*)-2-bromopentanedioic acid (4.00 g, 19.0 mmol, 1.00 equiv.) in *tert*-butyl acetate (130 mL). The reaction mixture was stirred at room temperature overnight. Diethyl ether (100 mL) and water (50 mL) were added to the reaction mixture, the layers were separated, and the organic phase was washed with a saturated aq. Na_2_CO_3_ solution to reach pH 7. The organic layer was then dried over MgSO_4_, filtered and concentrated under reduced pressure. The crude material was purified by column chromatography (silica gel, Pet.ether/EtOAc, 90 : 10 v/v) to give compound **7** (3.32 g, 10.3 mmol, 17% over 2 steps) as a clear oil. IR (*ν*_max_/cm^−1^, neat): 2978, 1726, 1367, 1255, 1136, 843, 755. *R*_f_ (Pet.ether/EtOAc, v/v 90 : 10): 0.34. ^1^H NMR (400 MHz, CDCl_3_) *δ* (ppm): 4.24 (dd, ^3^*J* = 8.4, 5.8 Hz, 1H, C*H*), 2.46–2.36 (m, 2H, C*H*_2_CO_2_), 2.27–2.16 (m, 2H, C*H*_2_CHBr), 1.49 (s, 9H, CHBrCO_2_^*t*^Bu), 1.46 (s, 9H, CH_2_CO_2_^*t*^Bu). ^13^C NMR (101 MHz, CDCl_3_) *δ* (ppm): 171.5 (CHBr*C*O_2_), 168.6 (*C*O_2_CH_2_), 82.6 (CH_2_CO_2_*C*(CH_3_)_3_), 80.9 (BrCHCO_2_*C*(CH_3_)_3_), 47.0 (CHBr), 32.9 (*C*H_2_CHBr), 30.0 (*C*H_2_CO_2_^*t*^Bu), 28.2 (BrCHCO_2_^*t*^Bu), 27.8 (CH_2_CO_2_C(*C*H_3_)_3_). HRMS (ESI^+^, *m*/*z*): calculated for M = C_13_H_23_^79^BrO_4_, [M + H]^+^: 345.0672, found 345.0671. Spectral data were in accordance with that reported in the literature.^25^

#### Tetra-*tert*-butyl-2,2′-(7,10-bis(2-(*tert*-butoxy)-2-oxoethy-l)-1,4,7,10-tetraazacyclododecane-1,4-diyl)diglutarate (**9**)

*cis*-DO_2_A(O^*t*^Bu)_2_ (**8**) (500 mg, 1.25 mmol, 1.00 equiv.) was synthesized from a modified literature procedure,^41^ and was added to K_2_CO_3_ (1.04 g, 7.49 mmol, 6.00 equiv.), arm **7** (1.13 g, 3.50 mmol, 2.80 equiv.) and potassium iodide (414 mg, 2.50 mmol, 2.00 equiv.) in anhydrous CH_3_CN (10 mL) and the reaction mixture was stirred at 80 °C overnight. Potassium salts were removed by centrifugation, and the supernatant was concentrated under reduced pressure to obtain an oil. The crude residue was purified by column chromatography (silica gel, neat CH_2_Cl_2_ to CH_2_Cl_2_/CH_3_OH 90 : 10 v/v, with an increment of 2%) to give compound **7** (823 mg, 0.93 mmol, 75%) as a yellow amorphous solid. IR (*ν*_max_/cm^−1^, neat): 3322, 2974, 2932, 2832, 1718, 1366, 1227, 1147. ^1^H NMR (400 MHz, CDCl_3_) *δ* (ppm): 3.41 (app. d, ^3^*J* = 10.0 Hz, 2H, 2 × CH), 3.34 (d, ^2^*J* = 17.3 Hz, 2H, NCH_2_CO_2_), 3.25–2.85 (m, 4H, 2 × CH_2_ cyclen), 2.79 (d, ^2^*J* = 17.3 Hz, 2H, NC*H*_2_CO_2_), 2.60–2.00 (m, 12H, 6 × CH_2_ cyclen), 2.00–1.80 (m, 4H, CO_2_C*H*_2_CH_2_CH), 1.65–1.50 (m, 4H, CO_2_CH_2_C*H*_2_CH), 1.50–1.35 (m, 54H, 18 × CH_3_). ^13^C NMR (101 MHz, CDCl_3_) *δ* (ppm): 174.9, 172.9, 172.8, 172.4, 172.3, 172.0 (*C*O_2_^*t*^Bu), 82.4, 82.3, 81.9, 81.8, 80.6, 80.5 (*C*(CH_3_)_3_), 60.2, 60.1 (CH), 55.8, 55.7, 55.0 (N*C*H_2_CO_2_^*t*^Bu), 52.6, 48.6(2), 48.5(7), 47.4, 47.3, 44.7, 44.5 (C*H*_2_ cyclen), 33.7 (CO_2_CH_2_*C*H_2_CH), 31.8 (CO_2_CH_2_*C*H_2_CH), 28.3, 28.2, 28.1, 28.0, 27.9(3), 27.8(6) (CH_3_). HRMS (ESI^+^, *m*/*z*) calculated for M = C_46_H_84_N_4_O_12_, [M + H]^+^: 885.6159, found 885.6158.

#### Gadolinium(iii)(2,2′-(7,10-bis(carboxylatomethyl)-1,4,7,10-tetraazacyclododecane-1,4-diyl)bis(5-((3-methacrylamidopropyl)-amino)-5-oxopentanoate)) (**10**)

##### Part A – synthesis of 2,2′-(7,10-bis(carboxymethyl)-1,4,7,10-tetraazacyclododecane-1,4-diyl)-diglutaric acid (**9**)

Ligand **8** (750 mg, 0.85 mmol, 1.00 equiv.) was stirred in TFA (10.0 mL) and H_2_O (1.00 mL) at room temperature for 3 days until complete deprotection (LC-MS analysis). The mixture was concentrated under reduced pressure and residual TFA was removed by successive co-evaporation with CH_2_Cl_2_. The resulting residue was dissolved in deionised water, centrifuged and filtered through a syringe filter (cut-off 220 nm). The filtrate was lyophilised to afford a white solid. The crude product **9** was used directly in the next step without further purification. IR (*ν*_max_/cm^−1^, neat): 3084, 2933, 2556, 1945, 1708, 1662, 1390, 1177, 1130. HRMS (ESI^+^, *m*/*z*): calculated for M = C_22_H_36_N_4_O_12_, [M + H]^+^: 549.2402, found 549.2402.

##### Part B

Deprotected ligand **9** (400 mg, 0.73 mmol, 1.00 equiv.) and gadolinium(iii)chloride hexahydrate (454 mg, 0.88 mmol, 1.20 equiv.) were stirred in water (10.0 mL) at room temperature for 24 hours. Over the course of the reaction, the pH was adjusted to 7 by addition of aq. HCl or aq. NaOH. The reaction mixture was lyophilised and the resulting solid was dissolved in deionised water, centrifuged twice and filtered through a syringe filter (220 nm cut-off). The supernatant was then lyophilised to afford the Gd complex **10** as a white amorphous solid. The crude product was directly engaged in the next reaction step without further purification. IR (*ν*_max_/cm^−1^, neat): 3400, 2980, 2838, 1684, 1600, 1396, 1205, 1128. HRMS (ESI^−^, *m*/*z*): calculated for M = C_22_H_32_^158^GdN_4_O_12_, [M]^−^: 702.1258, found 702.1246.

#### Gadolinium(iii) (2,2′-(7,10-bis(carboxylatomethyl)-1,4,7,10-tetraazacyclododecane-1,4-diyl)bis(5-((3-methacrylam-idopropyl)amino)-5-oxopentanoate)) (**Gd·L4**)

Gd(iii) complex **10** (200 mg, 0.29 mmol, 1.00 equiv.) and 2-(5-norborene-2,3-dicarboximido)-1,1,3,3-tetramethyluronium tetrafluoroborate or TNTU (219 mg, 0.60 mmol, 2.10 equiv.) were dissolved in DMF (5.00 mL) at room temperature. The solution was stirred for 30 minutes at 40 °C and DIPEA (199 μL, 1.14 mmol, 4.00 equiv.) was added, followed by *N*-(3-aminopropyl)methacrylamide hydrochloride (107 mg, 0.60 mmol, 2.10 equiv.). The reaction mixture was stirred for 1 hour at 40 °C and then at room temperature overnight. The desired Gd(iii) complex was precipitated in cold diethylether and collected by filtration. The solid was washed with cold Et_2_O and dried under vacuum. The crude residue was dissolved in water and purified by reverse phase preparative HPLC (gradient 0–100% acetonitrile in 25 mM NH_4_HCO_3_ over 10 minutes) to afford complex **Gd·L2** (68.2 mg, 71.8 μmol, 25% over 3 steps) as a white amorphous solid. IR (*ν*_max_/cm^−1^, neat): 3280, 3076, 2928, 2870, 1596, 1538, 1436, 1381, 1085. HRMS (ESI^−^, *m*/*z*): calculated for M = C_36_H_56_^158^GdN_8_O_12_, [M]^−^: 950.3259, found 950.3257. Analytical HPLC [gradient 0–100% acetonitrile in 25 mM NH_4_HCO_3_ over 10 minutes]: *t*_R_ = 5.533 minutes.

### Synthesis of linear and hyperbranched polymeric CAs

#### Representative example of the synthesis of linear P(NAM-*r*-**Gd·L1**) with a DP = 100 and molar ratio NAM : **Gd·L1** : CTA equal to 95 : 5 : 1

4-Acryloylmorpholine (67.1 mg, 60.0 μL, 0.5 mmol, 95.0 equiv.), **Gd·L1** (17.1 mg, 25.0 μmol, 5 equiv.), trioxane (3.8 mg, 41.7 μmol, 8.3 equiv.), cyano-4-[(dodecylsulfanyl-thiocarbonyl)-sulfanyl]pentanoic acid (2.0 mg, 5.0 μmol, 1.00 equiv.) and AIBN (0.2 mg, 1.0 μmol, 0.20 equiv.) were stirred in DMSO/H_2_O (80 : 20 v/v, 440 μL), the overall reaction volume being 0.5 mL, in a Schlenk tube equipped with a stirring bar. The reaction mixture was stirred at room temperature for 5 minutes and degassed with N_2_ through 3 successive freeze–pump–thaw cycles. The reaction mixture was then stirred at 80 °C overnight (15 h). The resulting polymer was purified by extensive dialysis against water (6 × 4 hours) and lyophilization to give pure polymer as an amorphous white solid. IR (*ν*_max_/cm^−1^, neat): 3433, 2963, 2856, 1626, 1439, 1233, 1111. All P(NAM-*r*-**Gd·L1**) copolymers displayed similar IR spectra and showed only small differences (±30 cm^−1^) in their stretches *ν*_max_ (ESI Section 2). ^1^H NMR (400 MHz, CDCl_3_) *δ* (ppm): 4.25–3.10 (8H, NC*H*_2_C*H*_2_O), 2.30–2.9 (1H, CH backbone), 2.00–1.00 (2H, CH_2_ backbone). All P(NAM-*r*-**Gd·L1**) copolymers displayed similar NMR spectra with identical peaks numbers and peak shifts. The only noticeable difference is an increase in peak broadness with an increase of the **Gd·L1** percentage in copolymer formulation (ESI Section 2). ^13^C NMR (101 MHz, CDCl_3_) *δ* (ppm): 172.9 (CONH), 66.9, 66.4 (CH_2_O), 46.1, 42.3 (CH_2_N), 35.5 (CH backbone), 34.8 (CH_2_ backbone). All linear P(NAM-*r*-**Gd·L1**) copolymers displayed similar ^13^C NMR spectra, with identical numbers of resonances and similar chemical shifts.

#### Representative example of the synthesis of an hyperbranched polymer with a crosslinker Gd complexes: synthesis of P(NAM-*r*-**Gd·L2**) with [M]_0_ = 2.00 M

4-Acryloylmorpholine (141.2 mg or 126 μL, 1.0 mmol, 99.1 equiv.), crosslinker **Gd·L2** (141.2 mg, 9.0 μmol, 0.9 equiv.), trioxane (7.5 mg, 83.0 μmol, 8.3 equiv.), poly(ethylene glycol)methyl ether 2-(dodecylthiocarbonothioylthio)-2-methylpropionate as CTA (10.6 mg, 9.6 μmol, 1.00 equiv.) and AIBN (0.3 mg, 1.9 μmol, 0.20 equiv.) were stirred in DMSO/H_2_O (80 : 20 v/v, 0.5 mL overall) in a Schlenk tube equipped with a stirring bar. The reaction mixture was stirred at room temperature for 5 minutes and degassed with N_2_ through 3 successive freeze–pump–thaw cycles. The reaction mixture was then stirred at 80 °C overnight (15 h). The resulting polymer was purified by extensive dialysis against water (6 × 4 hours) to give, after lyophilization, pure polymer as an amorphous white (to slightly yellow) solid. IR (*ν*_max_/cm^−1^, neat): 3433, 2963, 2856, 1630, 1436, 1231, 1111, 1029. All hyperbranched P(NAM-*r*-**Gd·L2–4**) polymers displayed similar IR spectra and showed only small differences (±30 cm^−1^) in their stretches *ν*_max_. ^1^H NMR (400 MHz, CDCl_3_) *δ* (ppm): 4.25–3.10 (8H, NC*H*_2_C*H*_2_O), 2.30–2.9 (1H, CH backbone), 2.00–1.00 (2H, CH_2_ backbone). All hyperbranched P(NAM-*r*-**Gd·L2–4**) copolymers displayed similar NMR spectra with identical peaks numbers and peak shifts. ^13^C NMR (101 MHz, CDCl_3_) *δ* (ppm): 172.9 (CONH), 66.9, 66.4 (CH_2_O), 46.1, 42.3 (CH_2_N), 35.5 (CH backbone), 34.8 (CH_2_ backbone). All linear P(NAM-*r*-**Gd·L2–4**) copolymers displayed similar ^13^C NMR spectra, with identical numbers of resonances and similar chemical shifts.

### Dynamic light scattering (DLS)

DLS measurements were performed with a Malvern Zetasizer Nano ZS using Zetasizer software (version 7.12). The Zetasizer system uses a Diode-pumped solid-state laser operating at a wavelength of 532 nm and an avalanche photodiode (APD) detector. The scattered light was detected at an angle of 175°. The temperature was stabilized to ±0.1 °C of the set temperature (25 °C). All aqueous polymer solutions were filtered prior to measurement, using a nylon syringe filter with 220 nm cut-off.

### Relaxometry measurements

#### NMRD

The observed water protons longitudinal relaxation rate constant (*R*^obs^_1_) values were measured as a function of the magnetic field strength in non-deuterated aqueous solutions on a Fast Field-Cycling Stelar SmarTracer relaxometer over a continuum of magnetic field strengths from 0.00024 to 0.25 T (corresponding to 0.01–10 MHz proton Larmor frequencies) at 25 and 37 °C by using the standard inversion recovery pulse sequence with 4 scans for each acquired data point. The relaxometer operates under computer control with an absolute uncertainty in 1/*T*_1_ of ±1%. To complete the data set, 6 ESI data† points were obtained by measurements at higher magnetic fields (precisely 20, 30, 40, 50, 60 and 70 MHz) on a Stelar relaxometer with a Spinmaster console connected to a Bruker WP-80 magnet (80 MHz/2 T) adapted to variable-field measurements. The temperature was set and controlled with a Stelar VTC-91 airflow heater and measured by a substitution technique using a copper-constantan thermocouple (error ± 0.1 °C). The exact concentration of Gd(iii) was determined by measurement of bulk magnetic susceptibility shifts of a *t*BuOH signal,^72^ or by inductively coupled plasma mass spectrometry. The variable temperature ^1^H NMR profiles were obtained by measuring the relaxation rate at different temperature from 5 to 75 °C (12 to 16 acquisition points) at a fixed magnetic field intensity (20 MHz or 30 MHz) using an inversion recovery method with a 90° pulse.

#### ^17^O NMR measurements

Variable-temperature ^17^O NMR measurements were recorded on a 500 MHz Bruker Avance III spectrometer (11.75 T) equipped with a 5 mm probe and standard temperature was regulated by air or nitrogen flow controlled by a Bruker BVT 3200 control unit. The samples were analyzed at 278 K and from 280 to 350 K with a 5 K increment (16 measurements). Concentrated aqueous solutions of complexes (10–20 mM) at physiological pH (7.4) and containing 2.0% of the ^17^O isotope (Cambridge Isotope) were used. The observed transverse relaxation rates (1/*T*_2_) were measured from the peak width at half-height. The fitting parameters were Δ^2^, *τ*_v_, the *τ*_M_ value at 298 K, its enthalpy of activation Δ*H*_M_, and the scalar Gd-^17^O_w_ coupling constant *A*/*ħ.*

## Conflicts of interest

There are no conflicts to declare.

## Supplementary Material

SC-012-D0SC04750C-s001
