# Supplementary material for: Polymerizable Gd(iii) building blocks for the synthesis of high relaxivity macromolecular MRI contrast agents
Source: Chem Sci. 2021 Feb 1;12(11):3999–4013. doi: 10.1039/d0sc04750c (PMC8179470; doi:10.1039/d0sc04750c)
Supplement: SC-012-D0SC04750C-s001 [file SC-012-D0SC04750C-s001.pdf]

Electronic Supplementary Material (ESI) for Chemical Science.
This journal is © The Royal Society of Chemistry 2021

Supporting Information

Polymerizable Gd(III) Buildings Blocks for the Synthesis of High Relaxivity Macromolecular MRI Contrast Agents.

Thomas R. Berki,[ab] Jonathan Martinelli,[c], Lorenzo Tei,[c] Helen Willcock*[a] and Stephen J. Butler*[b].

[a]Department of Chemistry, Loughborough University, Leicestershire, LE11 3TU, UK.

[b]Department of Materials, Loughborough University, Leicestershire, LE11 3TU, UK.

[c]Department of Science and Technological Innovation, Università del Piemonte Orientale, I15121, Alessandria, Italy.

Table of Contents

1 Instrumentation 3

2 Gd Complexes Synthesis and Characterisation 5

2.1 Synthesis of Cyclen derivatives: DO_3A, *cis*-DO_2A and *trans*-DO_2A. . . 5

2.2 Synthesis of α-Bromoester Arms Derived from Amino Acids 9

2.3 Synthesis of $Gd.L_{1-3}$ 12

2.4 Synthesis of $Gd.L_4$ 27

2.5 High Performace Liquid Chromatography 30

3 Characterisation of Linear and Hyperbranched polymeric CAs 31

4 Relaxometry Study Data 37

4.1 Acquisition of NMRD Profiles 37

4.2 Acquisition of Variable Temperature relaxometry profiles 37

4.3 NMRD Profiles Discrete Complexes 38

4.4 Fitted NMRD Profiles of $Gd.L_{1-4}$ 42

4.5 ^{17}O NMR Raw Data Gd Complexes 42

4.6 NMRD Raw Data and Fitted NMRD Profiles of Linear P(NAM-r-$Gd.L_1$) polymers 48

4.7 NMRD Raw Data and Fitted NMRD Profiles of Hyperbranched P(NAM-r-$Gd.L_{2-4}$) Polymers 53

4.8 Fitted NMRD profiles and NMRD Parameters obtained for the Partial Fitting (1-100 MHz) of Hyperbranched Systems NMRD Data Sets . . . 56

4.9 Fitted NMRD profiles and NMRD parameters obtained for the fitting of Hyperbranched Systems over the all magnetic field range 0.01-100 MHz NMRD Data Sets 59

4.10 VT Profiles of Linear Polymeric Systems 61

4.11 VT Profiles of Hyperbranched Polymeric Systems 62

4.12 Discussion over VT Profiles 63

5 Theoretical Models to Explain Paramagnetic Relaxation Enhancement 65

5.1 Discrete Complex NMRD Profiles - the SBM Theory 65

5.2 Macromolecules NMRD Profiles - the Lipari-Szabo Model 67

References **67**

1 Instrumentation

NMR. ^1H and ^{13}C nuclear magnetic resonance (NMR) spectra were recorded in commercially available deuterated solvents on a JEOL ECS-400 spectrometer (^1H at 399.782 MHz, ^{13}C at 100.525 MHz), a Bruker Advance Ultra-Shield 400 spectrometer (^1H at 400.134 MHz, ^{13}C at 100.624 MHz) and a JEOL ECZ-500R spectrometer (^1H at 500.160 MHz, ^{13}C at 125.765 MHz) at 293 K. Chemical shifts are expressed as δ in parts per million (ppm) and are referenced to the residual protio-solvent resonances ($CDCl_3$, ^1H: δ = 7.26 ppm; ^{13}C: δ = 77.16 ppm; D_2O, ^1H: δ = 4.79 ppm; CD_3OD, ^1H: δ = 3.31 ppm, ^{13}C: δ = 49.0 ppm). ^1H coupling constants (J) are reported in hertz (Hz). Assignment of signals in ^1H and ^{13}C spectra were resolved using ^1H-^1H correlated spectroscopy (COSY), distortionless enhancement by polarization transfer (DEPT), heteronuclear multiple quantum correlation (HMQC), heteronuclear multiple bond correlation (HMBC) and total correlation spectroscopy (TOCSY) experiments when appropriate.

IR. Infrared spectra were recorded using neat compounds on a Thermo Scientific Fourier Transform Infrared Spectrometer Nicolet iS5 equipped with a iD7 ATR module irradiating between 4000 and 600 cm^{-1}. Only relevant absorbances are reported. The data were recorded using OMNIC software.

Chromotography. Column chromatography was performed using flash silica gel 60 (particle size 40–63 microns) purchased from Apollo Scientific. Thin layer chromatography (TLC) was performed on aluminium sheet silica gel plates with 0.2 mm thick silica gel 60 F254 using the stated mobile phase. Preparative RP-HPLC was performed using a Waters 2489 UV/Visible detector performed at 254 nm, a Waters 1525 Binary HPLC pump controlled by the Waters Breeze 2 HPLC system software. Separation was achieved using a semi-preparative XBridge C18 (5 μm OBD 19 × 100 mm) column at a flow rate maintained at 17 mL/min. A solvent system composed of either water (0.05% formic acid) / acetonitrile (0.05% formic acid) or water (25 mM NH_4HCO_3) / acetonitrile was used over the stated linear gradient (usually 0 to 100% organic solvent over 10 minutes). Analytical RP-HPLC was performed using a XBridge C18 (5 μm 4.6 × 100 mm) column at a flow rate maintained at 2.0 mL/min using the stated gradient and solvents.

Mass spectrometry. Liquid Chromatography Electrospray mass spectra were recorded on a Shimadzu LC-2020 system and controlled using LabSolutions, version 5.86 SP1 software. The system operates in positive or negative ion mode, with acetonitrile as the carrier solvent. The flow rate was maintained at 0.7 mL/min over a gradient of 5 to 95% acetonitrile (0.03% formic acid) in water (0.03% formic acid) for 10 minutes. High resolution mass spectra were recorded using a Thermofisher Q-Exactive Orbitrap mass spectrometer.

Size exclusion chromatography. SEC analyses were performed on different systems. On an Agilent technologies 1260 Infinity system, comprising an Auto sampler 1260 ALS, a pump 1260 Iso pump, a column oven 1260 TCC, an Agilent PLgel 5 μm MIXED-C column, a reflexive index detector (RID). Each measurement was done by injection of 20 μL of a sample chloroform (2% TEA) solution, at 313 K, at a flow rate of 1 mL/min. The calibration was made with polystyrene standards (PS, Easivial PM, Agilent). Number average molecular weight (M_n) weight average molecular weight (M_w) and dispersity (Đ) were determined using the Agilent Technologies SEC software version 1.0. SEC data were also acquired on an Agilent 1260 Infinity II Multi-Detector GPC/SEC system with RI and ultraviolet (UV) detectors (λ = 309nm) and PLGel 3 μm (50 $\times$ 7.5 mm) guard column and two PLGel 5 μm (300 $\times$ 7.5 mm) mixed-C columns with DMF containing 5 mM NH_4BF_4 as the eluent (flow rate 1 mL/min, 50 °C). A calibration curve based on poly(methyl methacrylate) standards (PMMA, Easivial PM, Agilent) was applied for determination of molecular weights.

ICP-MS Measurements. The exact concentration of Gd(III) was also determined by inductively coupled plasma mass spectrometry (ICP-MS) with a Thermo Optek XSeries 2 spectrometer. Sample digestion was prepared by dissolving a known amount of the solid (around 1 mg) in fuming nitric acid (1.000 mL) in a 1 mL prescored ampule. The ampules were sealed and stored in an oven at 120 °C for 24 hours The resulting solutions were allowed to cool to room temperature and were diluted 400, 500, 4000 or 50000 fold, depending on the Gd concentration, to obtain [Gd] < 50 ppb. Before any measurement, the instrumental settings were optimised to reach maximal sensitivity for Gd. For quantitative determination, the isotopes of Gd and In (used as internal standard) were measured at m/z 157 and 115, respectively. A calibration curve was obtained by dilution of a 100 ppb gadolinium standard stock solution in an aqueous solution of nitric acid (1.0% *v*/*v*). The concentrations used to build the calibration curve were 0.5 ppb, 5 ppb, 25 ppb and 50 ppb.

2 Gd Complexes Synthesis and Characterisation

2.1 Synthesis of Cyclen derivatives: DO_3A, *cis*-DO_2A and *trans*-DO_2A.

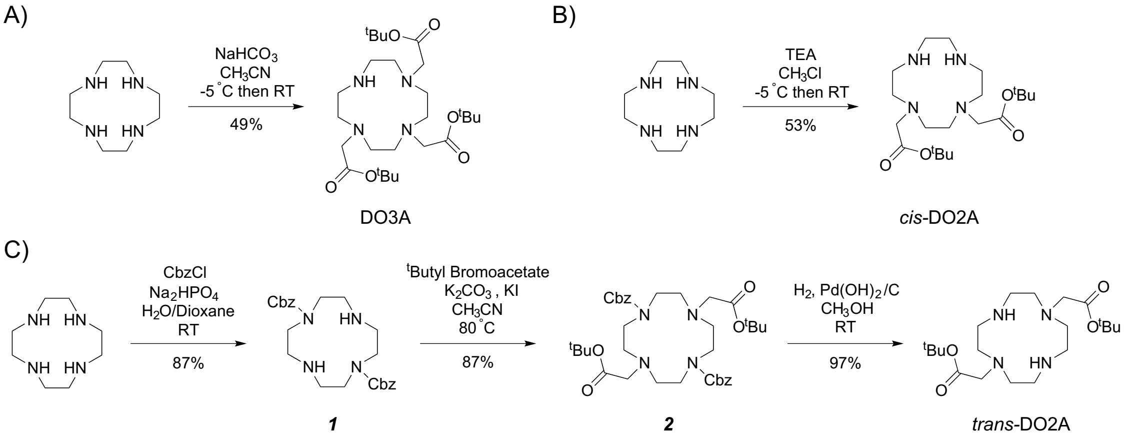

Scheme S1. Synthetic procedure for the preparation of macrocycles: A) **DO3A (1)**, B) ***cis*-DO2A (2)** and C) ***trans*-DO2A (5).**

2.1.1 Tri-*tert*-butyl-2,2',2"-(1,4,7,10-tetraazacyclododecane-1,4,7-triyl)-triacetate or DO3A (1)

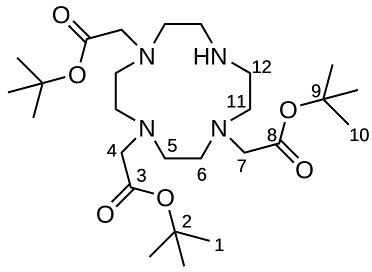

Compound **1** was synthesised following the modification of literature procedures.[1–4] Under an atmosphere of N_2, *tert*-Butylbromoacetate (5.06 mL, 34.3 mmol, 2.95 equiv.) was added dropwise over a period of 2 hours to a cold (−5 °C) stirred solution of cyclen (2.00 g, 11.6 mmol, 1.00 equiv.) and $NaHCO_3$ (2.93 g, 34.8 mmol, 3.00 equiv.) in anhydrous CH_3CN (40.0 mL). The reaction was then stirred for 72 hours at room temperature. Solids were removed by filtration and the solvent was evaporated under reduced pressure. The residue was purified by flash column chromatography (silica gel, pure CH_2Cl_2 to CH_2Cl_2/CH_3OH, 92:8 v/v, with an increment of 2%) to yield compound **1** (2.97 g, 5.78 mmol, 50%) as an amorphous solid. **IR (ν_{max}/cm^{-1}, neat):** 3650, 2980, 2736, 1728, 1716, 1575, 1450, 1364, 1253, 1143. ***R*$_f$ (CH_2Cl_2/CH_3OH, 90:10 v/v):** 0.44. **^{1}H NMR (400 MHz, $CDCl_3$) δ (ppm):** 3.32 (4H, s, H^7), 3.23 (2H, s, H^4), 3.00-2.75 (16H, m, $H^{5,6,11,12}$), 1.40(2) (18H, s, H^{10}), 1.39(6) (9H, s,

H^1). N-H signals not observed. **^{13}C NMR (101 MHz, $CDCl_3$) δ (ppm):** 170.5 (C^8), 169.6 (C^3), 81.8 (C^2), 81.7 (C^9), 58.2 (C^7), 51.3 (CH_2 cyclen), 51.2 (CH_2 cyclen), 49.1 (CH_2 cyclen), 47.5 (CH_2 cyclen), 48.7 (C^4), 28.2(3) (C^1), 28.2(0) (C^{10}). **HRMS (ESI$^+$, m/z):** calculated for $C_{26}H_{51}N_4O_6$, $[M+H]^+$:515.3803, found 515.3801.

Spectral data were in accordance with that reported in the literature.[1–3,5]

2.1.2 Di-*tert*-butyl-2,2'-(1,4,7,10-tetraazacyclododecane-1,4-diyl)diacetate or *cis*-DO2A (2)

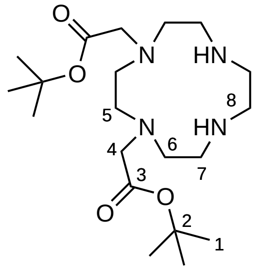

Compound **2** was synthesised following the modification of literature procedures.[6,7] 1,4,7,10-Tetraazacyclododecane (2.00 g, 11.6 mmol, 1.00 equiv.) and triethylamine (16.2 mL, 116 mmol, 10.0 equiv.) were dissolved in anhydrous $CHCl_3$ (200 mL) at room temperature. After 5 minutes, the reaction mixture was cooled down to −5 °C. A solution of *tert*-butyl bromoacetate (3.43 mL, 23.2 mmol, 2.00 equiv.) in $CHCl_3$ (25.0 mL) was added dropwise to the vigorously stirred reaction mixture, over a period of 2 hours at −5 °C. The reaction mixture was then stirred at room temperature overnight. Volatiles were evaporated and the resulting residue was dissolved in deionised water (100 mL). The pH was then adjusted to 3 by addition of aq. of HCl. Underised over-alkylated products were extracted with $CHCl_3$ (4 × 50 mL). pH was then adjusted to 6 by addition of aq. NaOH and the aqueous layer was washed again with $CHCl_3$ (4 × 50 mL) to remove remaining over-alkylated product. Finally, pH was adjusted to 7-8 and the desired product was extracted with $CHCl_3$ (4 × 50 mL). These organic layers were combined, dried over $MgSO_4$ and concentrated. The residue was purified by column chromatography (silica gel, CH_2Cl_2/CH_3OH 98:2 v/v to CH_2Cl_2/CH_3OH, 84:16 v/v, with an increment of 2%) to yield compound **2** (2.46 g, 6.13 mmol, 53%) as a slightly orange amorphous solid. **IR (ν_{max}/cm^{-1}, neat):** 3314, 2974, 2853, 1723, 1596, 1457, 1367, 1147. **^{1}H NMR (400 MHz, $CDCl_3$) δ (ppm):** 3.34 (4H, s, H^4), 3.10-2.96 (8H, m, 4 × CH_2 cyclen), 2.96-2.80 (8H, m, 4 × CH_2 cyclen), 1.43 (18H, s, H^1). N-H signals are not observed. **^{13}C NMR (101 MHz, $CDCl_3$) δ (ppm):** 170.4 (C^3), 81.9 (C^2), 53.5 (C^4), 51.2, 49.9, 46.6, 46.2 (CH_2 cyclen), 28.3 (C^1). **HRMS (ESI$^+$, m/z):** calculated for $C_{20}H_{41}N_4O_4$, $[M+H]^+$:401.3122, found 401.3121.

Spectral data were in accordance with that reported in the literature.[6,7]

2.1.3 Dibenzyl 1,4,7,10-tetraazacyclododecane-1,7-dicarboxylate (3)

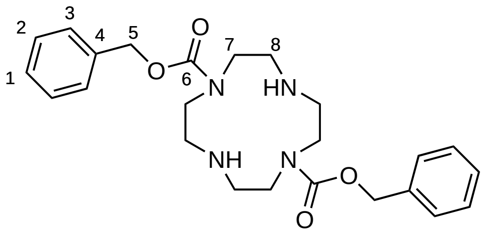

Compound **3** was synthesised following the modification of literature procedures.[6,8–10] Cyclen (5.00 g, 29.0 mmol, 1.00 equiv.) and Na_2HPO_4 (14.0 g, 98.7 mmol, 3.40 equiv.) were dissolved in a mixture H_2O/Dioxane (50:20, 70 mL). pH was then adjusted to 2.5 by careful addition of aq. HCl. The reaction mixture was stirred for 5 minutes at room temperature, and a solution of benzyl chloroformate (8.50 mL, 59.5 mmol, 2.05 equiv.) in dioxane (20.0 mL) was added dropwise over 4 hours. After the addition, the reaction mixture was stirred overnight at room temperature and then concentrated to dryness. The resulting residue was then dissolved in H_2O (200 mL) and the pH was adjusted to 7 by addition of aq. KOH. The aqueous solution was washed with Et_2O (2 × 200 mL) and extracted with CH_2Cl_2 (2 × 200 mL). The first CH_2Cl_2 fraction (the only fraction containing pure product), was dried over $MgSO_4$, filtered and concentrated under reduced pressure to yield compound **3** (11.1 g, 25.2 mmol, 87%) as a white solid. **IR (ν_{max}/cm^{-1}, neat):** 3356, 3248, 3186, 2980, 2860, 1694, 1412, 1260, 1121, 1003. **^{1}H NMR (400 MHz, CDCl$_3$)** δ **(ppm):** 7.50-7.30 (10H, m, $H^{1,2,3}$), 5.16 (4H, d, ^4J = 3.2 Hz, H^5), 3.90-3.50 (8H, m, H^7), 3.20-2.80 (8H, m, H^8). N-H signals are not observed. **^{13}C NMR (101 MHz, CDCl$_3$)** δ **(ppm):** 156.3 (C^{6a}), 156.2 (C^{6b}), 136.0 (C^4), 128.8(3) (C^{2a} or C^{3a}), 128.7(8) (C^{3a} or C^{2a}), 128.6 (C^{1a}), 128.5 (C^{1b}), 128.2 (C^{2b} or C^{3b}), 128.1 (C^{3b} or C^{2b}), 68.0 (C^{5a}), 67.9 (C^{5b}), 50.7, 50.5, 50.3, 49.9, 49.7, 49.2, 49.0 (CH_2 cyclen). **HRMS (ESI$^+$, m/z):** calculated for $C_{24}H_{33}N_4O_4$, $[M+H]^+$:441.2496, found 441,2496.

Spectral data were in accordance with that reported in the literature.[6,8–10]

2.1.4 Intermediate Dibenzyl 4,10-bis(2-(*tert*-butoxy)-2-oxoethyl)-1,4,7,10-tetra-azacyclododecane-1,7-dicarboxylate (4)

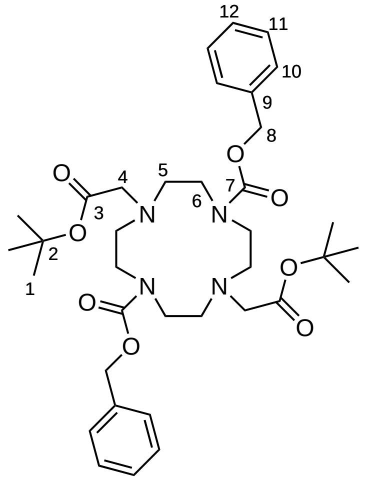

Compound **4** was synthesised following the modification of literature procedures.[9,11,12] Bis-amine **3** (11.1 g, 25.2 mmol, 1.00 equiv.) and potassium carbonate (13.9 g, 101 mmol, 4.00 equiv.) were dissolved in anhydrous CH_3CN (150 mL) at room temperature. *tert*-Butyl bromoacetate (7.63 mL, 51.6 mmol, 2.05 equiv.) was added and the reaction mixture was stirred at 60 °C overnight. Solids were removed by filtration and the filtrate was concentrated under reduced pressure to obtain a yellow oil. The crude residue was purified by flash column chromatography (silica gel, pure CH_2Cl_2 to CH_2Cl_2/CH_3OH, 98:2 v/v, with an increment of 0.5%) to yield compound **4** (14.1 g, 21.1 mmol, 84%) as a yellow liquid. **IR (**ν_{max}**/cm**$^{-1}$**, neat):** 2979, 2932, 2733, 1693, 1454, 1420, 1245, 1147. **^{1}H NMR (400 MHz, $CDCl_3$) δ (ppm):** 7.40-7.20 (10H, m, $H^{10,11,12}$), 5.08 (4H, s, H^8), 3.38 (8H, m, H^6), 3.30-3.10 (4H, m, H^4), 2.95-2.70 (8H, m, H^5), 1.39 (18H, s, H^1). **^{13}C NMR (101 MHz, $CDCl_3$) δ (ppm):** 170.4 (C^3), 156.3 (C^7), 136.8 (C^9), 128.4 (C^{11} or C^{10}), 127.9 (C^{12}), 127.8 (C^{10} or C^{11}), 80.8 (C^2), 66.9 (C^8), 56.0 (C^4), 54.3, 54.0, 46.9, 46.6 (CH_2 cyclen), 28.1 (C^1). **HRMS (ESI$^+$, m/z):** calculated for $C_{36}H_{53}N_4O_8$, $[M+H]^+$:669.3858, found 669.3868.

Spectral data were in accordance with that reported in the literature.[9,11,12]

2.1.5 Di-*tert*-butyl-2,2'-(1,4,7,10-tetraazacyclododecane-1,7-diyl)diacetate (5)

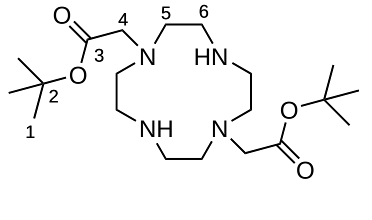

Compound **5** was synthesised following the modification of literature procedures.[6,9,13] Compound **4** (14.1 g, 21.1 mmol, 1.00 equiv.) and $Pd(OH)_2/C$ (1.48 g, 2.11 mmol, 0.10 equiv.) was suspended in CH_3OH (38.0 mL) at room temperature. The atmosphere was purged with argon and the inert gas was then replaced by hydrogen. The reaction mixture was stirred at room temperature for 3 days. The catalyst was removed by 2 successive filtrations through pads of Celite®. The filtrate was concentrated under reduced pressure and the residue was dissolved in acidic water (pH 3, HCl, 150 mL). The aqueous solution was washed with ethyl acetate (2 × 50.0 mL) and CH_2Cl_2 (2 × 50.0 mL) to remove of organic impurities (*e.g.* remaining starting material, over-alkylated product). The pH of the water phase was then adjusted to 6 and washed again with CH_2Cl_2 (3 × 50.0 mL) to remove other impurities. Finally, the pH was adjusted to 9-10 and the product was back extracted with CH_2Cl_2 (5 × 50.0 mL). The CH_2Cl_2's fractions (only) were combined, dried over $MgSO_4$ and concentrated under reduced pressure, to yield compound **5** (7.31 g, 18.2 mmol, 86%) as a viscous oil. **IR (ν_{max}/cm^{-1}, neat):** 3456, 3281, 3222, 2976, 2821, 1721, 1473, 1145. **^{1}H NMR (400 MHz, $CDCl_3$) δ (ppm):** 3.38 (4H, s, H^4), 2.92 (8H, m, H^5), 2.73 (8H, m, H^6), 1.45 (18H, s, H^1). N-H signals are not observed. **^{13}C NMR (101 MHz, $CDCl_3$) δ (ppm):** 171.2 (C^3), 81.5 (C^2), 57.1 (C^4), 52.1 (C^5), 47.2 (C^6), 28.3 (C^1). **HRMS (ESI$^+$, m/z):** calculated for $C_{20}H_{41}N_4O_4$, $[M+H]^+$:401.3122, found 401.3120.

Spectral data were in accordance with that reported in the literature.[6,9,13]

2.2 Synthesis of α-Bromoester Arms Derived from Amino Acids

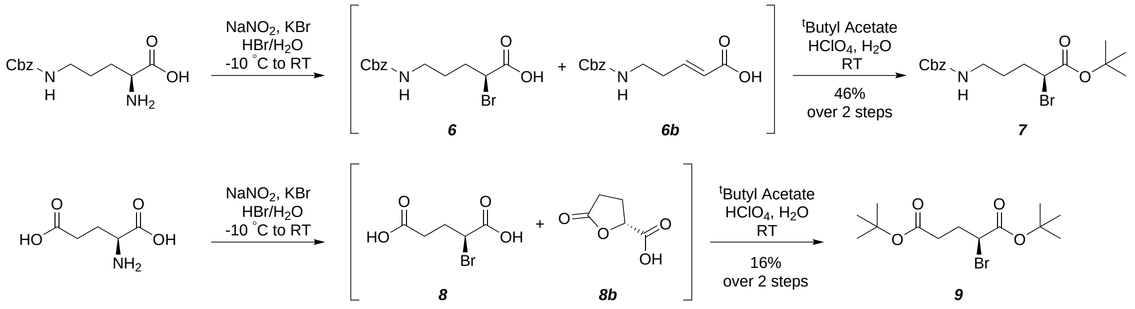

Scheme S2. Synthetic procedure of linker arms, 7 and 9.

2.2.1 (*S*)-5-(((benzyloxy)carbonyl)amino)-2-bromopentanoic (6)

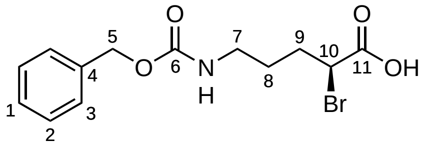

Compound **6** was synthesised following the modification of literature procedures.[14–17] A solution of sodium nitrite (2.59 g, 37.6 mmol, 2.00 equiv.) in water (5 mL) was added drop-wise over two hours to a cold (−10 °C) stirred solution of (*S*)-2-amino-5-(((benzyloxy)carbonyl)amino)pentanoic acid (5.00 g, 18.8 mmol, 1.00 equiv.), potassium bromide (8.27 g, 69.6 mmol, 3.70 equiv.), bromohydric acid (9.40 mL, 47.0 mmol, 2.50 equiv., 48% w/w in water) in water (100 mL). The resulting yellow solution was stirred at room temperature overnight. The reaction mixture was then extracted with diethyl ether (3 × 100 mL). The combined organic phases were washed with brine (100 mL), dried over $MgSO_4$, filtered and concentrated under reduced pressure to yield as a viscous slightly yellow liquid. The crude product was used directly in the next step without further purification. For analytical purpose, a portion of the crude material was purified by flash column chromatography (silica gel, CH_2Cl_2/Acetic acid, 99:1 v/v) to afford compound **6** as a colourless oil. **IR (ν_{max}/cm^{-1}, neat):** 3310, 3084, 3026, 2982, 1740, 1596, 1493, 733, 694. ***R*f (CH_2Cl_2/CH_3COOH, 95:5, v/v):** 0.35. **^{1}H NMR (400 MHz, $CDCl_3$) δ (ppm):** 7.60-7.28 (5H, m, $H^{1,2,3}$), 5.51 (2H, s, H^5), 4.21 (1H, dd, ^3J = 8.4, 6.4 Hz, H^{10}), 3.79 (2H, t, ^3J = 7.2 Hz, H^7), 2.15-1.80 (2H, m, H^9), 1.75-1.50 (2H, m, H^8). O-H and N-H signals are not observed. **^{13}C NMR (101 MHz, $CDCl_3$) δ (ppm):** 173.8 (C^{11}), 154.0 (C^6), 134.5 (C^4), 129.1 (C^1), 128.9 (C^2 or C^3), 128.8 (C^3 or C^2), 70.1 (C^5), 44.3 (C^{10}), 39.8 (C^7), 31.7 (C^9), 24.9 (C^8). **HRMS (ESI$^+$, m/z):** calculated for $C_{13}H_{16}{}^{79}BrNNaO_4$, $[M+Na]^+$:352.0155, found 352.0159.

2.2.2 *tert*-Butyl (*S*)-5-(((benzyloxy)carbonyl)amino)-2-bromopentanoate (7)

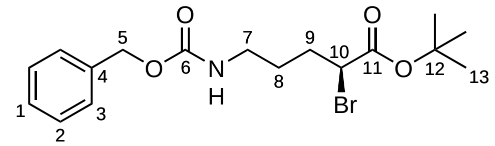

Compound **7** was synthesised following the modification of a literature procedure.[18] $HClO_4$ (70% w/w in H_2O, 70 μL, 0.81 mmol, 0.05 equiv.) was added to a solution of (*S*)-5-(((benzyloxy)carbonyl)amino)-2-bromopentanoic acid **6** (5.33 g, 15.1 mmol, 1.00 equiv.) in *tert*-butyl acetate (60.0 mL). The reaction mixture was stirred at room temperature for 18 hours. Diethyl ether (100 mL) and water (50 mL) were added to the reaction mixture. The organic phase was isolated and washed with a saturated aq. solution of Na_2CO_3 until neutral pH is obtained. The organic layer was then dried over $MgSO_4$, filtered and concentrated under reduced pressure. The residue was then purified by flash column chromatography (silica gel, Pet. ether/EtOAc, 2:1 v/v) to give compound **7** as a colourless oil (3.32 g, 8.60 mmol, 46% over 2 steps). **IR (ν_{max}/cm^{-1}, neat):** 3341, 2940, 1705, 1528, 1250, 1141, 741, 694. ***R*f (Pet. ether/EtOAc v/v 70:30):** 0.42. **^{1}H NMR (400 MHz, $CDCl_3$) δ (ppm):** 7.49-7.28 (5H, m, $H^{1,2,3}$), 5.08 (2H, s,

H^5), 4.12 (1H, t, ^3J = 7.2 Hz, H^{10}), 3.22 (2H, app. q, ^3J = 6.7 Hz, H^7), 2.15-1.80 (2H, m, H^9), 1.75-1.50 (2H, m, H^8), 1.46 (9H, s, H^{13}). N-H signals are not observed. **^{13}C NMR (101 MHz, $CDCl_3$) δ (ppm):** 168.8 (C^{11}), 156.5 (C^6), 136.6 (C^4), 128.7 (C^2), 128.3 (C^1 & C^3), 82.7 (C^{12}), 66.9 (C^5), 47.2 (C^{10}), 40.3 (C^7), 32.1 (C^9), 27.9 (C^{13} & C^8). **HRMS (ESI$^+$, m/z):** calculated for $C_{17}H_{24}{}^{79}BrNNaO_4$, [M+Na]$^+$:408.0781, found 408.0779.

2.2.3 Di-*tert*-butyl (*S*)-2-bromopentanedioate (9)

Part A - (*S*)-2-bromopentanedioic acid (**8**)

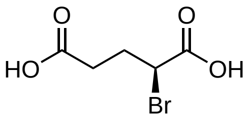

Compound 8 was synthesised following the modification of a literature procedure.[19] L-glutamic acid (9.21 g, 62.6 mmol, 1.00 equiv.) and KBr (22.4 g, 188 mmol, 3.00 equiv.) were dissolved in aq.HBr (1.00 M, 143 mL, 143 mmol, 2.30 equiv.) at room temperature. The reaction mixture was stirred for 5 minutes and cooled to −10 °C. A solution of $NaNO_2$ (10.8 g, 157 mmol, 2.50 equiv.) in water (15 mL) was added dropwise over 2 hours. After the addition, the reaction mixture was stirred at room temperature overnight. H_2SO_4 (4.00 mL) was added slowly and the resulting aqueous solution was extracted with Et_2O (3 × 100 mL). The combined organic layers were washed with brine (2 × 75 mL), dried over $MgSO_4$, filtered and concentrated under reduced pressure. The crude residue (4.00 g) was used directly in the next step without further purification.

Part B - Di-*tert*-butyl(*S*)-2-bromopentanedioate (**9**)

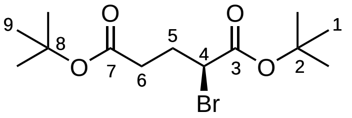

Compound 9 was synthesised following the modification of a literature procedure.[19] Aquous $HClO_4$ (70% w/w, 125 μL, 1.42 mmol, 0.07 equiv.) was added to a solution of (*S*)-2-bromopentanedioic acid **8** (4.00 g, 19.0 mmol, 1.00 equiv.) in *tert*-butyl acetate (130 mL). The reaction mixture was stirred at room temperature overnight. Diethyl ether (100 mL) and water (50 mL) were added to the reaction mixture, the layers were separated and the organic phase was washed with a saturated aq. Na_2CO_3 solution, until the pH was 7. The organic layer was then dried over $MgSO_4$, filtered and concentrated under reduced pressure. The crude material was then purified by column chromatography (silica gel, pet.ether/EtOAc, 90:10 v/v) to give compound **9** (3.32 g, 10.3 mmol, 17% over 2 steps) as a clear viscous oil. **IR (ν_{max}/cm^{-1}, neat):** 2978, 1726,

1367, 1255, 1136, 843, 755. ***R*~f~ (Pet.ether/EtOAc, v/v 90:10):** 0.34. **^{1}H NMR (400 MHz, CDCl$_3$) δ (ppm):** 4.24 (1H, dd, ^{3}J = 8.4, 5.8 Hz, H^{4}), 2.46-2.36 (2H, m, H^{6}), 2.27-2.16 (2H, m, H^{5}), 1.49 (9H, s, H^{1}), 1.46 (9H, s, H^{9}). **^{13}C NMR (101 MHz, CDCl$_3$) δ (ppm):** 171.5 (C^{3}), 168.6 (C^{7}), 82.6 (C^{8}), 80.9 (C^{2}), 47.0 (C^{4}), 32.9 (C^{5}), 30.0 (C^{6}), 28.2 (C^{1}), 27.8 (C^{9}). **HRMS (ESI^{+}, m/z):** calculated for $C_{13}H_{24}{}^{79}BrO_4$, [M+H]$^{+}$:345.0672, found 345.0671.

Spectral data were in accordance with that reported in the literature.[19]

2.3 Synthesis of Gd.L$_{1-3}$

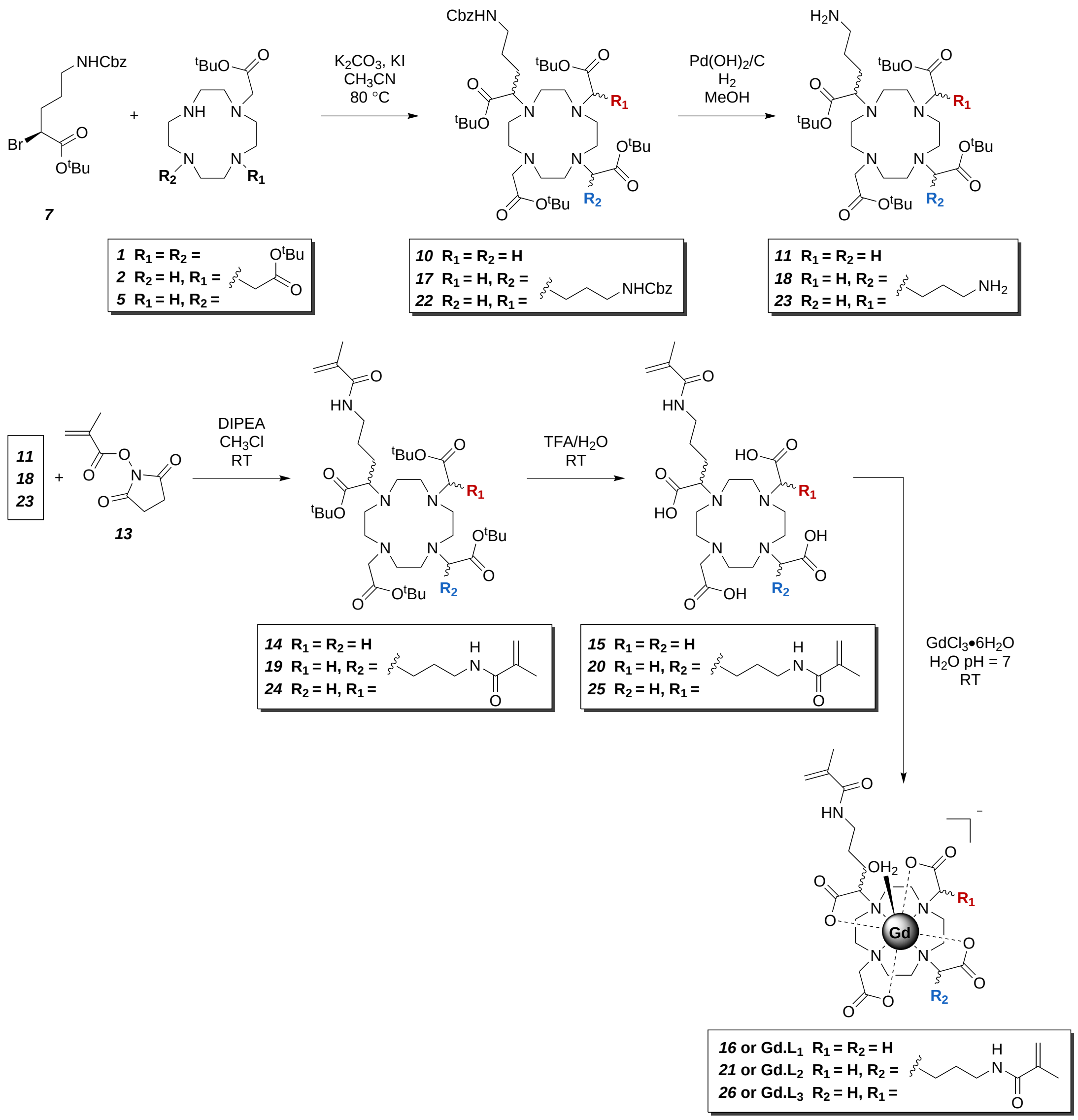

Scheme S3. Synthesis of Gd.L$_{1-3}$.

2.3.1 Tri-*tert*-butyl 2,2',2"-(10-(5-(((benzyloxy)carbonyl)amino)-1-(*tert*-butoxy-1-oxopentan-2-yl)-1,4,7,10-tetraazacyclododecane-1,4,7-triyl)triacetate (10)

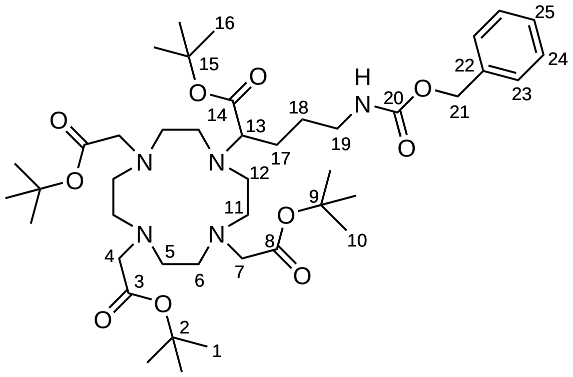

Compound **1** (700 mg, 1.36 mmol, 1.00 equiv.), K_2CO_3 (568 mg, 4.08 mmol, 3.00 equiv.), KI (226 mg, 1.36 mmol, 1.00 equiv.) and compound **7** (1.05 g, 2.72 mmol, 2.00 equiv.) were suspended in anhydrous CH_3CN (6.00 mL) and the reaction mixture was stirred at 80 °C overnight. The solids were removed by centrifugation and the surpernatant was evaporated under reduced pressure. The crude residue was purified by flash column chromatography (silica gel, pure CH_2Cl_2 to CH_2Cl_2/CH_3OH, 96:4 v/v, with an increment of 1% and then to CH_2Cl_2/CH_3OH, 92/8 v/v, with an increment of 2%) to afford compound **10** (907 mg, 1.10 mmol, 81%) as an amorphous yellow solid. **IR (**ν_{max}**/cm**$^{-1}$**, neat):** 3248, 2975, 2933, 2827, 1719, 1226, 1158. R_f **(**CH_2Cl_2/CH_3OH**, 90:10 v/v):** 0.36. ^{1}**H NMR (400 MHz, CDCl**$_3$**)** δ **(ppm):** 7.50-7.20 (5H, m, $H^{23,24,25}$), 5.04 (2H, s, H^{21}), 3.44-3.24 (4H, m, $H^{4a,7a,13}$), 3.20-3.01 (4H, m, H^{19} & CH_2 cyclen), 3.05-2.87 (2H, m, CH_2 cyclen), 2.85-2.65 (3H, m, $H^{4b,7b}$), 2.60-2.00 (12H, m, 6 × CH_2 cyclen), 1.83 (1H, m, H^{18a}), 1.68 (1H, m, H^{17a}), 1.62-1.47 (2H, m, $H^{17b,18b}$), 1.47-1.30 (36H, m, $H^{1,10,16}$). N-H signals are not observed. ^{13}**C NMR (101 MHz, CDCl**$_3$**)** δ **(ppm):** 175.1 (C^{14}), 172.9 (C^8), 172.8 (C^3), 156.7 (C^{20}), 136.9 (C^{22}), 128.5 (C^{23} or C^{24}), 128.0 (C^{24} or C^{23}), 127.9 (C^{25}), 82.1 ($C(CH_3)_3$), 82.0 ($C(CH_3)_3$), 81.9 ($C(CH_3)_3$), 66.3 (C^{21}), 61.1 (C^{13}), 55.9, 55.8, 55.6 (NCH_2COOtBu), 52.7, 52.5, 48.6, 48.2, 47.3, 43.9 (NCH_2CH_2N), 40.8 (C^{19}), 29.8 (C^{18}), 27.9(3), 27.9(1), 27.8(6) (CH_3), 22.0 (C^{17}). **HRMS (ESI**$^+$**, m/z):** calculated for $C_{43}H_{73}N_5NaO_{10}$, $[M+Na]^+$:842.5250, found 842.5256.

2.3.2 Tri-*tert*-butyl 2,2',2"-(10-(5-amino-1-(*tert*-butoxy)-1-oxopentan-2-yl)-1,4-7,10-tetraazacyclododecane-1,4,7-triyl)triacetate (11)

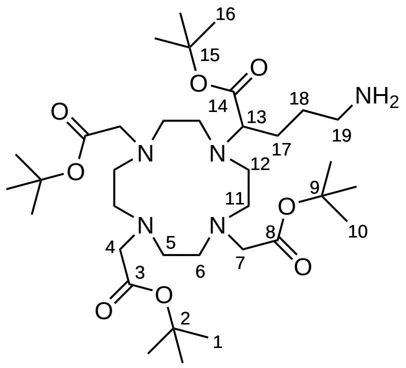

Product **10** (178 mg, 0.22 mmol, 1.00 equiv.) was dissolved in CH_3OH (2 mL) and $Pd(OH)_2$ (30.5 mg, 22.0 μmol, 0.10 equiv.) was added. The reaction mixture was purged with N_2. The inert atmosphere was replaced with H_2 and the reaction mixture was stirred for 48 hours at room temperature. The suspension was filtered through a pad of Celite® and rinsed with CH_3OH. The filtrate was then pushed through a syringe filter (cut-off 220 nm) to remove the remaining catalyst nanoparticles. The solvent was removed under reduced pressure to yield compound **11** (128 mg, 0.19 mmol, 86%) as a yellow oil. **IR (ν_{max}/cm^{-1}, neat):** 2979, 2828, 1720, 1453, 1366, 1226, 1156. **^{1}H NMR (400 MHz, $CDCl_3$) δ (ppm):** 3.45-3.31 (4H, m, $H^{4a,7a,13}$), 3.31-3.01 (4H, m, 2 × CH_2), 2.92-2.73 (3H, m, $H^{4b,7b}$), 2.68 (2H, app. dt, ^3J = 6.8, 2.5 Hz, H^{19}), 2.60-2.00 (12H, m, 6 × CH_2), 1.90-1.50 (4H, m, $H^{17,18}$), 1.50-1.35 (36H, m, $H^{1,10,16}$). N-H signals are not observed. **^{13}C NMR (101 MHz, $CDCl_3$) δ (ppm):** 175.2 (C^{14}), 172.9(8) (C^8 or C^3), 172.8(9) (C^3 or C^8), 82.1, 82.0, 81.9 ($C(CH_3)$), 61.1 (C^{13}), 55.9 (C^7 or C^4), 55.6 (C^4 or C^7), 52.8, 52.6, 48.8, 48.4, 47.4, 45.0 (CH_2 cyclen), 42.1 (C^{19}), 33.4 (C^{18}), 28.0, 27.9 (CH_3), 22.2 (C^{17}). **HRMS (ESI$^+$, m/z):** calculated for $C_{35}H_{68}N_5O_8$, $[M+H]^+$:686.5062, found 686.5076.

2.3.3 2,5-Dioxopyrrolidin-1-yl methacrylate or NMAS (13)

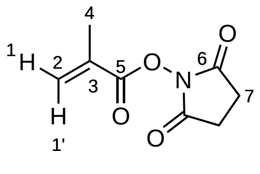

Compound **13** was synthesised following the modification of literature procedures.[20–23] A solution of EDC (11.7 g, 61.0 mmol, 1.05 equiv.) and DMAP (355 mg, 2.90 mmol, 0.05 equiv.) in CH_2Cl_2 (25 mL) was added dropwise to a cold (0 °C) stirred solution of methacrylic acid (5.00 g, 58.1 mmol, 1.00 equiv.) and *N*-hydroxysuccinimide (8.02 g, 69.7 mmol, 1.20 equiv.) in CH_2Cl_2 (100 mL) over 1 hour at 0 °C. After the reaction mixture was stirred at room temper-

ature for 24 hours. The reaction mixture was then washed with water (3 × 100 mL), dried over $MgSO_4$, filtered and concentrated. The residue was purified by flash column chromatography (silica gel, CH_2Cl_2/THF, 9:1 v/v) to give compound **13** as a white solid (9.81 g, 53.6 mmol, 92%). **IR** (**ν_{max}/cm^{-1}, neat):** 2979, 1756, 1729, 1636, 1423, 1366, 1204, 1080, 980. ***R*f (CH_2Cl_2):** 0.20. **^{1}H NMR (400 MHz, $CDCl_3$) δ (ppm):** 6.40 (1H, d, ^2J = 0.8 Hz, $H^{1'}$), 5.88 (1H, d, ^2J = 0.8 Hz, H^1), 2.84 (4H, s, H^7), 2.04 (3H, s, H^4). **^{13}C NMR (101 MHz, $CDCl_3$) δ (ppm):** 169.4 (C^6), 162.3 (C^5), 131.9 (C^3), 130.6 (C^2), 25.7 (C^7), 18.3 (C^4). **HRMS (ESI$^+$, m/z):** calculated for $C_8H_9NNaO_4$, $[M+Na]^+$:206.0424, found 206.0424.

Spectral data were in accordance with that reported in the literature.[20–23]

2.3.4 tri-*tert*-butyl 2,2',2"-(10-(1-(*tert*-butoxy)-5-methacrylamido-1-oxopentan-2-yl)-1,4,7,10-tetraazacyclododecane-1,4,7-triyl)triacetate (14)

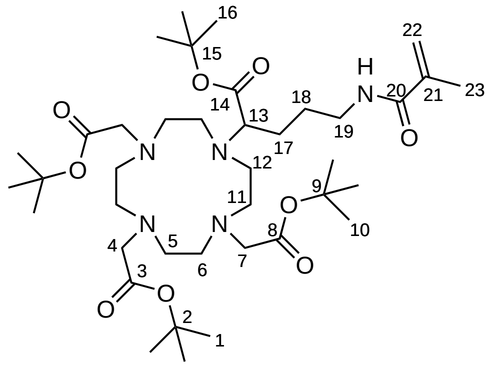

Compound **11** (334 mg, 0.49 mmol, 1.00 equiv.) and DIPEA (170 μL, 0.98 mmol, 2.00 equiv.) were dissolved in $CHCl_3$ (5.00 mL). The reaction mixture was stirred for 5 minutes at 40 °C, and a solution of activated ester **13** (93.6 mg, 0.51 mmol, 1.05 equiv.) in CH_2Cl_2 (2.00 mL) was added dropwise over 2 hours. The reaction mixture was stirred at 40 °C for 3 hours and then at room temperature overnight. The solution was diluted with CH_2Cl_2 (100 mL), water (95 mL) and a saturated solution of ammonium chloride (5 mL). The organic phase was separated, dried over $MgSO_4$, filtered and concentrated. The residue was then purified by flash column chromatography (silica gel, pure CH_2Cl_2 to CH_2Cl_2/CH_3OH, 92:8 v/v with a 2% increment) to yield compound **14** (326 mg, 0.43 mmol, 89%) as a yellow amorphous solid. **IR** (**ν_{max}/cm^{-1}, neat):** 3278, 2976, 2828, 1720, 1660, 1618, 1525, 1367, 1226. ***R*f (CH_2Cl_2/CH_3OH v/v 90:10):** 0.38. **^{1}H NMR (400 MHz, $CDCl_3$) δ (ppm):** 5.86 (1H, s, H^{22}), 5.28 (1H, s, $H^{22'}$), 3.60-3.10 (6H, m, $H^{4a,7a,13,19}$), 3.10-2.90 (2H, m, CH_2 ring), 2.90-2.60 (5H, m, $H^{4b,7b}$ & CH_2 cyclen), 2.60-2.00 (12H, m, 6 × CH_2 cyclen), 1.98 (3H, s, H^{23}), 1.95-1.5 (4H, m, $H^{17,18}$), 1.42 (36H, app. s, $H^{1,10,16}$). N-H signals are not observed. **^{13}C NMR (101 MHz, $CDCl_3$) δ (ppm):** 175.4 (C^{14}), 173.0, 172.8, 172.7 (CH_2*C*O), 169.1 (C^{20}), 139.9 (C^{21}), 120.1 (C^{22}), 82.1, 82.0, 81.9 (*C*(CH_3)), 61.4 (C^{13}), 55.9 (C^7), 55.6 (C^4), 52.8, 52.6, 48.6, 48.3, 47.4, 44.8 (CH_2 cyclen),

39.4 (C^{19}), 29.5 (C^{18}), 27.9 ($C^{1,10,16}$), 22.8 (C^{17}), 19.4 (C^{23}). **HRMS (ESI$^+$, m/z):** calculated for $C_{39}H_{72}N_5O_9$, $[M+H]^+$:754.5325, found 754.5300.

2.3.5 2,2',2"-(10-(1-Carboxy-4-methacrylamidobutyl)-1,4,7,10-tetraazacyclo-dodecane-1,4,7-triyl)triacetic acid (15)

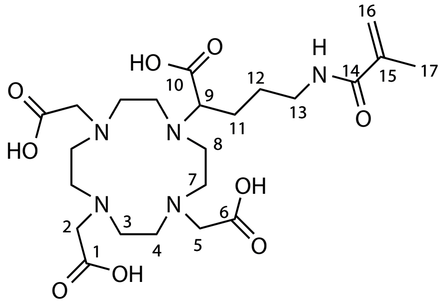

Compound **14** (125 mg, 0.16 mmol, 1.00 equiv.) was dissolved in TFA (5 mL) and H_2O (0.5 mL) and stirred for 3 days at room temperature until complete deprotection (determined by LC-MS). The reaction mixture was concentrated under reduced pressure and residual TFA was removed by successive co-evaporations with CH_2Cl_2 (5 × 20 mL). The resulting residue was suspended in deionised water, centrifuged twice, and filtrated through a syringe filter (220 nm cut-off), and purified by reverse phase HPLC [gradient system A: H_2O (0.1% v/v HCOOH)/CH_3CN (0.1% v/v HCOOH)]. The fractions containing the product were combined and lyophilised to yield compound **15** (52.4 mg, 98.9 μmol, 59%) as a white amorphous solid. **IR (ν_{max}/cm^{-1}, neat):** 3273, 3077, 1588, 1383, 1315, 1084. **^{1}H NMR (500 MHz, D_2O) δ (ppm):** 5.75 (1H, s, H^{16}), 5.49 (1H, s, $H^{16'}$), 4.02 (1H, dd, ^3J = 10.0, 3.1 Hz, H^9), 3.96-3.80 (4H, m, H^5), 3.80-3.60 (2H, m, H^2), 3.60-3.36 (8H, m, 4 × CH_2 cyclen), 3.32 (2H, t, ^3J = 6.3 Hz, H^{13}), 3.28-2.90 (8H, m, 4 × CH_2 cyclen), 1.99 (3H, s, H^{17}), 1.91-1.80 (1H, m , H^{11a}), 1.80-1.60 (3H, m, $H^{11b,12}$. O-H and N-H signals are not observed. **HRMS (ESI$^+$, m/z):** calculated for $C_{23}H_{40}N_5O_9$, $[M+H]^+$:530.2821, found 530.2831. **HPLC [gradient system B, H_2O (0.1% v/v HCOOH)/CH_3CN(0.1% v/v HCOOH)]:** t_R = 2.3 minutes.

2.3.6 Gadolinium(III) (2,2',2"-(10-(1-carboxylato-4-methacrylamido-butyl)1,4-7,10-tetraazacyclododecane-1,4,7-triyl)triacetate) (16)

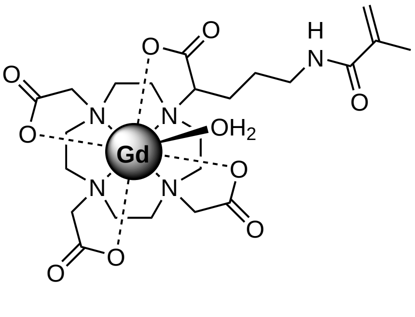

Deprotected ligand **15** (77.0 mg, 0.15 mmol, 1.00 equiv.) and gadolinium(III) chloride hexahydrate (60.0 mg, 0.16 mmol, 1.10 equiv.) were stirred in deionized water (10.0 mL) at room temperature for 24 hours. Over the course of the reaction, pH was adjusted to 7 by addition of aq. HCl or aq. NaOH. The reaction mixture was lyophilised and the resulting solid was dissolved in deionised water, centrifuged, filtered through a syringe filter (220 nm cut-off) and purified by preparative HPLC [gradient system A: H_2O [25 mmol NH_4HCO_3)/CH_3CN (see section 2.5)]. The fractions containing the product were combined to yield, after lyophilisation, compound **16** (52.6 mg, 77.0 μmol, 51.4%) as a white amorphous solid. **IR (ν_{max}/cm^{-1}, neat):** 3273, 2981, 2920, 2865, 1586, 1382, 1316, 1084. **HRMS (ESI^{-}, m/z):** calculated for $C_{23}H_{35}^{158}GdN_5O_9$, [M]$^{-}$:683.1671, found 683.1661.**HPLC [gradient system B, H_2O (25 mM NH_4HCO_3)/CH_3CN)]:** t_R = 5.70 minutes. **Relaxivity (H_20, 60 MHz, pH = 7.4, 37 °C)**: r_1 = 4.8 mM^{-1}s^{-1}.

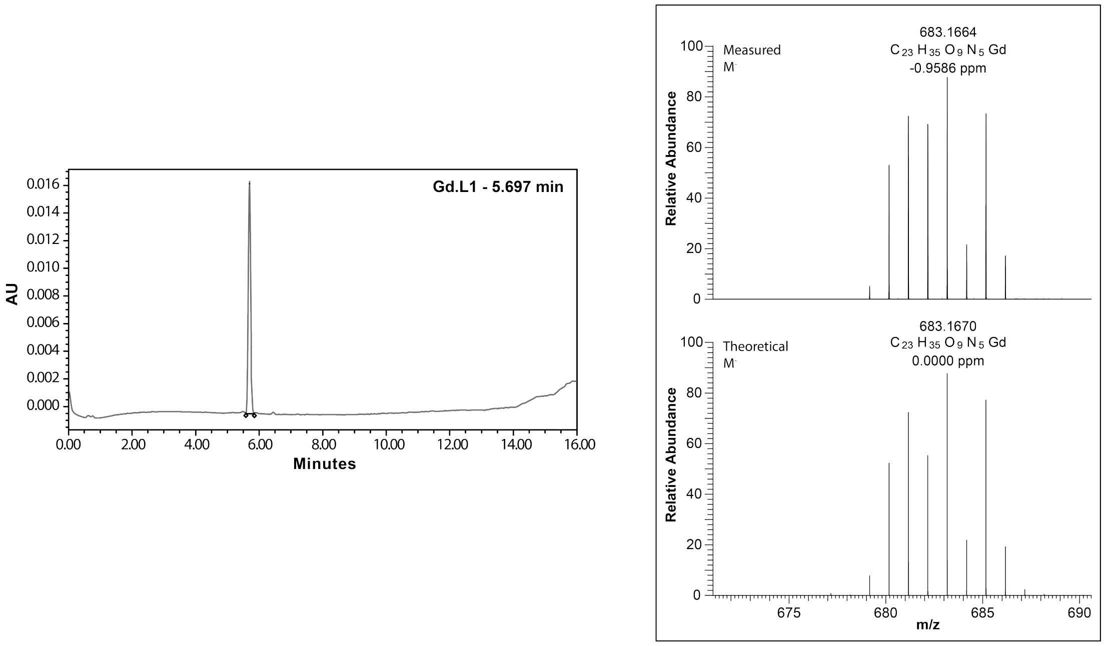

Figure S1. HPLC trace and HRMS spectra of **Gd.L**$_1$.

2.3.7 Di-*tert*-butyl-2,2'-(4,10-bis(2-(*tert*-butoxy)-2-oxoethyl)-1,4,7,10-tetraazacyclododecane-1,7-diyl)bis(5-(((benzyloxy)carbonyl)amino)pentanoate) (17)

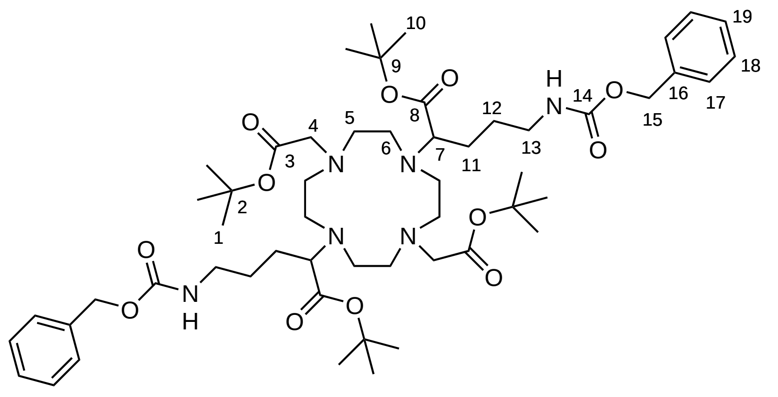

Compound **5** (800 mg, 2.00 mmol, 1.00 equiv.), K_2CO_3 (1,66 g, 12.0 mmol, 6.00 equiv.), compound **7** (2.01 g, 5.20 mmol, 2.60 equiv.), and potassium iodide (663 mg, 4.00 mmol, 2.00 equiv.) were dissolved in anhydrous CH_3CN (10.0 mL), and the reaction mixture was stirred at 80 °C overnight. Potassium salts were removed by centrifugation, and the supernatant was concentrated under reduced pressure to obtain a yellow oil. The crude material was then purified by flash column chromatography (silica gel, pure CH_2Cl_2 to CH_2Cl_2/CH_3OH, 92:8 v/v, with an increment of 2%) to yield compound **17** (1.76 mg, 1.74 mmol, 87%) as a yellow solid. **IR (ν_{max}/cm^{-1}, neat):** 3254, 2975, 2933, 2837, 1711, 1517, 1227, 1154, 1113. ***R*f (CH_2Cl_2/CH_3OH, v/v 90:10):** 0.32. **^{1}H NMR (400 MHz, $CDCl_3$) δ (ppm):** 7.40-7.26 (10H, m, $H^{17,18,19}$), 5.06 (4H, s, H^{15}), 3.42 (2H, d, ^2J = 17.1 Hz, H^{4A}), 3.32 (2H, m, H^7), 3.19 (4H, m, H^{13}), 3.07 (2H, app. t, J^{app} = 13.3 Hz, CH_2 cyclen), 2.94 (2H, app. t, J^{app} = 13.3 Hz, CH_2 cyclen), 2.76 (2H, d, ^2J = 17.1 Hz, H^{4B}), 2.65-2.25(8H, m, 4 × CH_2 cyclen), 2.20 (2H, app. d, J^{app} = 13.3 Hz, CH_2 cyclen), 2.08 (2H, app.d, J^{app} = 13.3 Hz, CH_2 cyclen), 1.86 (2H, m, H^{12a}), 1.69 (2H, m, H^{11a}), 1.53-1.65 (4H, m, $H^{11b,12b}$), 1.53-1.30 (36H, m, $H^{1,10}$). N-H signals are not observed. **^{13}C NMR (101 MHz, $CDCl_3$) δ (ppm):** 174.9 (C^8), 172.9 (C^3), 156.6 (C^{14}), 136.8 (C^{16}), 128.5, 128.0 (CH CBz benzyl ring), 82.1, 81.9 ($C(CH_3)_3$), 66.4 (C^{15}), 61.0 (C^7), 56.0 (C^4), 52.7, 48.8, 47.3, 44.6 (CH_2 cyclen), 40.8 (C^{13a}), 40.7 (C^{13b}), 29.8 (C^{12}), 28.3, 28.2, 27.9, (CH_3), 22.0 (C^{11}). **HRMS (ESI$^+$, m/z):** calculated for $C_{54}H_{86}N_6NaO_{12}$, $[M+Na]^+$:1033.6196, found 1033.6189.

2.3.8 Di-*tert*-butyl-2,2'-(4,10-bis(2-(*tert*-butoxy)-2-oxoethyl)-1,4,7,10-tetraaza-cyclododecane-1,7-diyl)bis(5-aminopentanoate) (18)

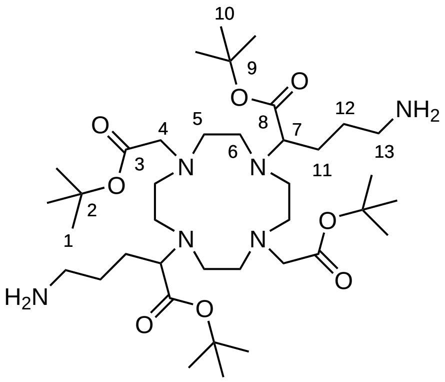

Compound **17** (50.0 mg, 0.49 mmol, 1.00 equiv.) and $Pd(OH)_2$ (10.0 mg, 74 μmol, 0.15 equiv.) were dissolved in CH_3OH (1.50 mL) at room temperature. The reaction mixture was purged with N_2 and the inert atmosphere was replaced with H_2. The reaction mixture was stirred overnight at room temperature and the catalyst was removed by filtration through a pad of Celite® and rinsed with CH_3OH. The resulting solution was filtered through a syringe filter (220 nm cut-off) and the filtrate was concentrated under reduced pressure to yield compound **18** (35.0 mg, 0.47 mmol, 95%) as a yellow oil. **IR (ν_{max}/cm^{-1}, neat):** 3368, 2977, 2932, 2844, 1718, 1577, 1455, 1367, 1227, 1155. **^{1}H NMR (400 MHz, $CDCl_3$) δ (ppm):** 3.41 (2H, d, ^2J = 17.3 Hz, H^{4a}), 3.37-3.33 (2H, m , H^7), 3.22 (2H, app. t, J = 13.4 Hz, CH_2 cyclen), 2.92 (2H, app. t, J = 13.0 Hz, CH_2 cyclen), 2.75 (2H, d, ^2J = 17.3 Hz, H^{4b}), 2.67 (4H, m, H^{13}), 2.57-2.31 (8H, m, 4 × CH_2 cyclen), 2.26 (2H, app. d, J = 13.6 Hz, CH_2 cyclen), 2.09 (2H, app. d, J = 14.0 Hz, CH_2 cyclen), 1.90-1.53 (8H, m, $H^{11,12}$), 1.50-1.35 (36H, m, $H^{1,10}$). N-H signals are not observed. **^{13}C NMR (101 MHz, $CDCl_3$) δ (ppm):** 174.8 (C^8), 174.0 ($C^{8'}$), 173.2 (C^3), 172.7 ($C^{3'}$), 83.0, 82.3, 81.8, 81.7 ($C(CH_3)_3$), 61.0 (C^7), 56.3, 55.9, 55.8 (NCH_2CO), 52.6, 48.7, 47.1, 46.9, 44.5 (CH_2 cyclen), 41.7 (C^{13}), 32.9 (C^{12}), 28.1, 27.7, 27.6 (CH_3), 22.1 (C^{11}). **HRMS (ESI$^+$, m/z):** calculated for $C_{38}H_{75}N_6O_8$, $[M+H]^+$:743.5641, found 743.5648.

2.3.9 Gadolinium(III) (2,2'-(4,10-bis(carboxylatomethyl)-1,4,7,10-tetraazacy-clododecane-1,7-diyl)bis(5-methacrylamidopentanoate)) (21)

Part A - Di-*tert*-butyl-2,2'-(4,10-bis(2-(*tert*-butoxy)-2-oxoethyl)-1,4,7,10-tetraazacyclododeca-ne-1,7-diyl)bis(5-methacrylamidopentanoate) (**19**)

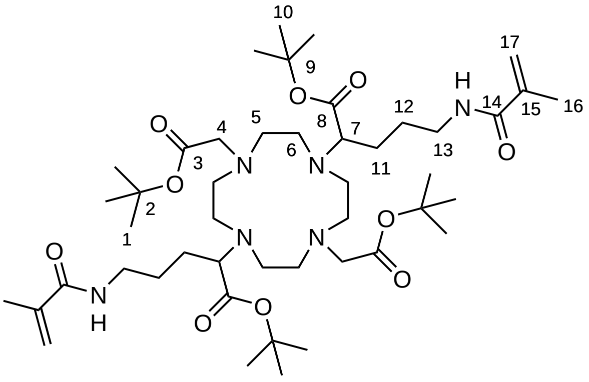

Bis-amine **18** (540 mg, 0.73 mmol, 1.00 equiv.) was dissolved in DMF (2.00 mL) at room temperature. DIPEA (506 μL, 2.91 mmol, 4.00 equiv.) and **13** (NHS-methacrylate) (280 mg, 1.53 mmol, 2.10 equiv.) were added. The reaction mixture was stirred at 30 °C overnight under an atmosphere of N_2. The solvent was evaporated and the residue was dissolved with CH_2Cl_2 (40.0 mL). The organic phase was washed with a mixture of water (20.0 mL) and a saturated solution of NH_4Cl (2.00 mL), dried over $MgSO_4$, filtered and concentrated. The desired compound was not stable on silica gel and therefore the product was used directly in the next step with no further purification. **IR (ν_{max}/cm^{-1}, neat):** 3257, 2976, 2929, 2852, 1716, 1656, 1615, 1533, 1227, 1151. **HRMS (ESI$^+$, m/z):** calculated for $C_{54}H_{86}N_6NaO_{12}$, $[M+Na]^+$:901.5985, found 901.5971.

Part B - 2,2’-(4,10-Bis(carboxymethyl)-1,4,7,10-tetraazacyclododecane-1,7-diyl)bis-(5-methacrylamidopentanoic acid) (**20**)

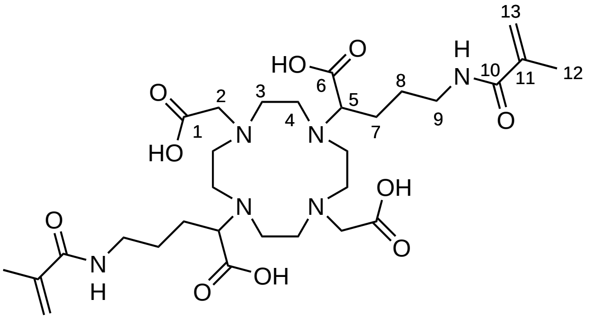

Ligand **19** (600 mg, 0.68 mmol, 1.00 equiv.) was dissolved in TFA (10.0 mL) and H_2O (1.00 mL) and was stirred for 3 days at room temperature until complete deprotection (LC-MS monitoring). The reaction mixture was concentrated under reduced pressure and residual TFA was removed by successive co-evaporations with CH_2Cl_2 (5 × 20 mL). The resulting residue was dissolved in deionised water, centrifuged twice, and the supernatant filtered through a syringe filter (cut-off of 220 nm), and the filtrate was lyophilised to afford **20** (354 mg) as a white solid . The crude product was directly used in the next step with no further purification. **HRMS (ESI$^+$, m/z):** calculated for $C_{30}H_{50}N_6NaO_{10}$, $[M+Na]^+$:655.3631, found 655.3680.

Part C - Gadolinium(III) (2,2'-(4,10-bis(carboxylatomethyl)-1,4,7,10-te-traazacyclododecane-1,7-diyl)bis(5-methacrylamidopentanoate)) (**21**)

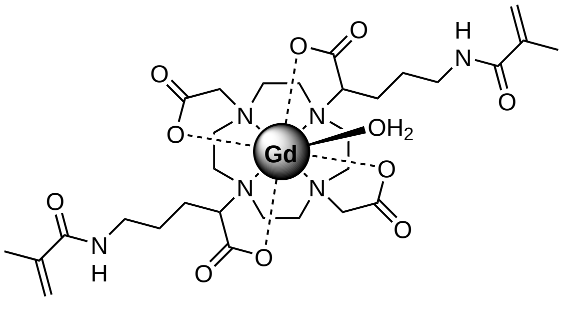

Deprotected ligand **20** (354 mg, 0.54 mmol, 1.00 equiv.) and gadolinium(III) chloride hexahydrate (221 mg, 0.60 mmol, 1.10 equiv.) were stirred in deionized water (10.0 mL) for 24 hours at room temperature. Over the course of the reaction, the pH was adjusted to 7 by addition of aq. HCl or aq. NaOH. The reaction mixture was lyophilised and the resulting solid was redissolved in deionised water, and centrifuged twice, the supernatant was filtered through a syringe filter (220 nm cut-off) and purified by preparative HPLC [gradient system B: H_2O (25 mM NH_4HCO_3)/CH_3CN (see section 2.5)]. The fractions containing the product were combined to yield, after lyophilisation, compound **21** (103 mg, 128 μmol, overall yield 24% over 3 steps) as a white powder. **IR (ν_{max}/cm^{-1}, neat):** 3286, 2980, 2866, 1592, 1389. **HRMS (ESI$^-$, m/z):** calculated for $C_{30}H_{46}{}^{158}GdN_6O_{10}$, $[M]^-$:808.2511, found 808.2543. **HPLC [gradient system B, H_2O (25 mM NH_4HCO_3)/CH_3CN)]:** t_R = 5.350 minutes. **Relaxivity (H_20, 60 MHz, pH = 7.4, 37 °C)**: r_1 = 5.8 mM^{-1}s^{-1}.

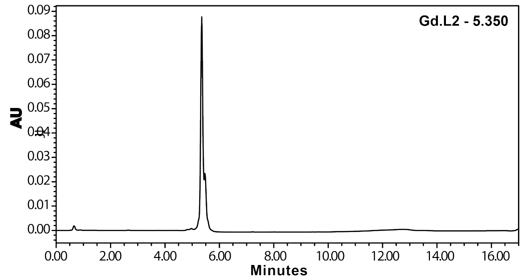

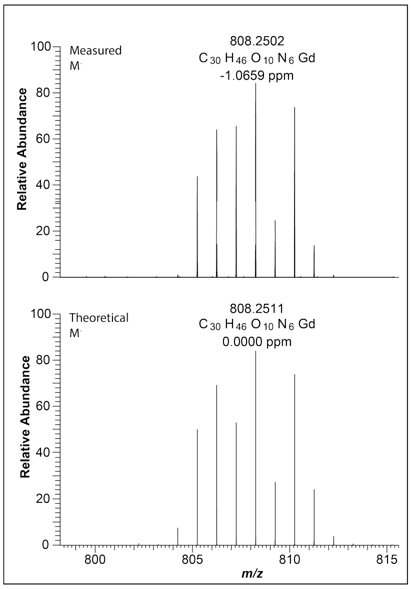

Figure S2. HPLC trace and HRMS spectra of **Gd.L**$_2$.

2.3.10 Di-*tert*-butyl-2,2'-(7,10-bis(2-(*tert*-butoxy)-2-oxoethyl)-1,4,7,10-tetraazacyclododecane-1,4-diyl)bis(5-(((benzyloxy)carbonyl)amino)pentanoate) (22)

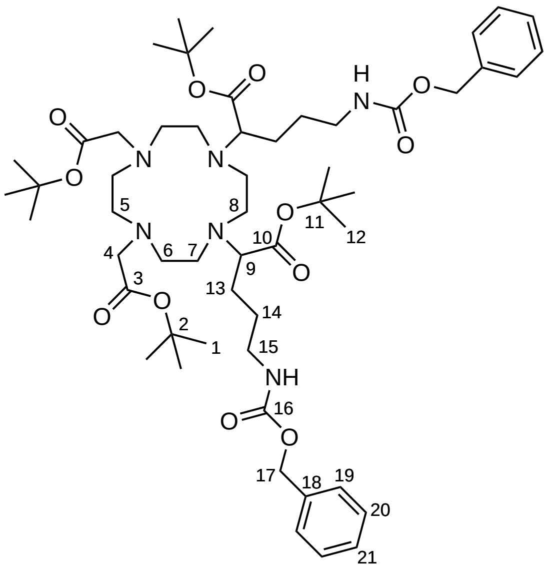

Compound **2** (600 mg, 1.50 mmol, 1.00 equiv.), K_2CO_3 (1.24 g, 9.00 mmol, 6.00 equiv.), KI (497 mg, 3.00 mmol, 2.00 equiv.) and compound **7** (1.74 g, 4.50 mmol, 3.00 equiv.) were suspended in anhydrous CH_3CN (5.00 mL) and the reaction mixture was stirred overnight at 80 °C. Solids were removed by centrifugation and the surpernatant was concentrated under reduced pressure. The crude material was then purified by column chromatography (silica gel, pure CH_2Cl_2 to CH_2Cl_2/CH_3OH, 96:4 v/v, with an increment of 1% and then to CH_2Cl_2/CH_3OH, 88:12 v/v, with an increment of 2%) to yield compound **22** (1.30 g, 1.29 mmol, 86%) as a yellowish amorphous solid. **IR (ν_{max}/cm^{-1}, neat):** 3256, 2979, 2931, 2851, 1711. 1517, 1453, 1366, 1228, 1156. **^{1}H NMR (400 MHz, CDCl$_3$) δ (ppm):** 7.50-7.20 (10H, m, $H^{19,20,21}$), 5.07 (4H, s, H^{17}), 3.45-3.27 (4H, m, $H^{4a,9}$), 3.16 (4H, m, H^{15}), 3.12-2.84 (4H, m, 2 × CH_2 cyclen), 2.76 (1H, d, ^2J = 17.1 Hz, H^{4b}), 2.73 (1H, d, ^2J = 17.1 Hz, $H^{4b'}$), 2.60-2.00 (12H, m, 6 × CH_2 cyclen), 1.97-1.78 (2H, m, H^{14a}), 1.76-1.50 (6H, m, $H^{13a,13b,14b}$), 1.40 (36H, m, $H^{1,12}$). N-H signals are not observed. **^{13}C NMR (101 MHz, CDCl$_3$) δ (ppm):** 175.0 (C^{10}), 172.8 (C^3), 156.6 (C^{16}), 136.8 (C^{18}), 136.7 ($C^{18'}$), 128.5, 128.0, 127.9 (CH Cbz benzene ring), 82.2, 82.1, 82.0, 81.9 ($C(CH_3)_3$), 66.5(C^{17}), 66.4 ($C^{17'}$), 62.2 (C^9), 61.3 ($C^{9'}$), 55.8 (C^4), 55.7 ($C^{4'}$), 52.6, 52.5, 48.6, 47.5, 47.4, 44.9, 44.8 (CH_2 cyclen), 40.8 (C^{15}), 40.6 ($C^{15'}$), 29.9 (C^{14}), 29.8 ($C^{14'}$), 28.2, 28.0, 27.9, 27.8 ($C^{1,12,1',12'}$), 21.7, 21.6 ($C^{13,13'}$). **HRMS (ESI$^+$, m/z):** calculated for $C_{54}H_{86}N_6NaO_{12}$, $[M+Na]^+$:1033.6196, found 1033.6193.

2.3.11 Di-*tert*-butyl-2,2'-(7,10-bis(2-(*tert*-butoxy)-2-oxoethyl)-1,4,7,10-tetraazacyclododecane-1,4-diyl)bis(5-aminopentanoate) (23)

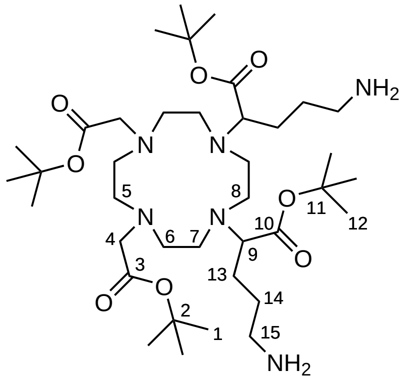

Compound **22** (1.30 g, 1.29 mmol, 1.00 equiv.) was dissolved in CH_3OH (10.0 mL) and $Pd(OH)_2$ (542 mg, 0.39 mmol, 0.30 equiv.) was added. The reaction mixture was purged with N_2 and replaced with H_2. The reaction mixture was stirred under an atmosphere of H_2 at room temperature for 72 hours. The catalyst was removed by filtration through a very large pad of Celite ® (two successive filtrations were required). The filtrate was concentrated *in vacuo* to approximately 10 mL, filtered again through a syringe filter (220 nm cut-off) and concentrated under reduced pressure to yield compound **23** (925 mg, 1.25 mmol, 97%) as a yellow oil. **IR (ν_{max}/cm^{-1}, neat):** 3413, 2979, 2931, 1716, 1366, 1226, 1151. **^{1}H NMR (400 MHz, $CDCl_3$) δ (ppm):** 3.50-3.31 (4H, m, $H^{4a,4a',9}$, 3.31-3.00 (4H, m, 2 × CH_2 cyclen), 2.81 (1H, d, ^2J = H^{4b}), 2.77 (1H, d, ^2J = $H^{4b'}$), 2.74-2.60 (4H, m, H^{15}), 2.60-2.00 (12H, m, 6 × CH_2 cyclen), 1.90-1.50 (8H, m, $H^{13,14}$), 1.30-1.50 (36H, m, $H^{1,12}$). N-H signals are not observed. **^{13}C NMR (101 MHz, $CDCl_3$) δ (ppm):** 175.3 (C^{10}), 173.0 ($C^{3'}$), 172.9 ($C^{3'}$), 82.6, 82.0, 81.9, 81.8 ($C(CH_3)_3$), 61.6 (C^9), 55.9 (C^4), 52.7, 52.5, 48.7, 47.6, 47.4, 45.1 (CH_2 cyclen), 42.2 (C^{15}), 33.7 (C^{14}), 33.6 ($C^{14'}$), 28.3, 28.0, 27.9 (CH_3), 21.9 (C^{13}). **HRMS (ESI$^+$, m/z):** calculated for $C_{38}H_{75}N_6O_8$, $[M+H]^+$:743.5641, found 743.5640.

2.3.12 Di-*tert*-butyl-2,2'-(7,10-bis(2-(*tert*-butoxy)-2-oxoethyl)-1,4,7,10-tetraazacyclododecane-1,4-diyl)bis(5-methacrylamidopentanoate) (24)

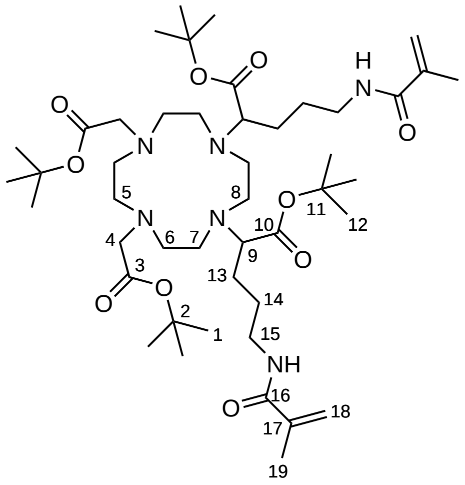

Compound **23** (800 mg, 1.08 mmol, 1.00 equiv.) and DIPEA (751 μL, 4.31 mmol, 4.00 equiv.) were dissolved in CH_3Cl (5.00 mL). The reaction mixture was stirred for 5 minutes at 40 °C, and a solution of activated ester **13** (414 mg, 2.26 mmol, 2.10 equiv.) in CH_3Cl (5.00 mL) was added dropwise over a period of 2 hours. The reaction mixture was stirred at 40 °C for 3 hours and then at room temperature overnight. Volatiles were evaporated under reduced pressure and the residue was dissolved in with CH_2Cl_2 (100 mL). The organic phase was washed with a mixture of water and saturated aq. NH_4Cl (100 mL, 95:5) solution, then dried over $MgSO_4$, filtered and concentrated. The residue was purified by flash column chromatography (silica gel, pure CH_2Cl_2 to CH_2Cl_2/CH_3OH, 92:8 v/v with a 2% increment) to yield compound **24** (373 mg, 0.42 mmol, 40%) as a yellow amorphous solid. Due to the loss of product during purification, if this reaction was to be repeated, we advise to not purify the product by column chromatography and use it directly after the aqueous work-up in the following step. **IR (ν_{max}/cm^{-1}, neat)**: 3329, 2979, 2934, 2827, 1717, 1656, 1532, 1454, 1367, 1225, 1151, 1031. **^{1}H NMR (400 MHz, $CDCl_3$) δ (ppm):** 5.90-5.70 (2H, m, H^{18}), 5.35-5.20 (2H, m, $H^{18'}$), 3.55-3.15 (8H, m, $H^{4a,4a',9,15}$), 2.00-3.05 (18H, m, $H^{4b,4b',5,6,7,8}$), 2.00-1.85 (6H, m, $H^{19,19'}$), 1.85-1.50 (8H, m, $H^{13,14}$), 1.50-1.30 (36H, m, $H^{1,12}$). N-H are not observed. **^{13}C NMR (101 MHz, $CDCl_3$) δ (ppm):** 175.5 (C^{10}), 175.2 ($C^{10'}$), 172.8 (C^3), 172.6 ($C^{3'}$), 169.2 (C^{16}), 169.0 ($C^{16'}$), 140.0 (C^{17}), 120.0 (C^{18}), 82.2, 82.1, 82.0, 81.9 ($C(CH_3)_3$), 61.5 (C^9), 61.4 ($C^{9'}$), 55.8 (C^4), 52.7, 48.5, 48.4, 47.6, 47.5, 44.9, 44.6 (CH_2 cyclen), 39.6, 39.5, 39.3, 39.1 (CH_2NH), 29.9, 29.6, 29.4 (CH_2CH_2NH), 28.4, 28.2, 28.1, 27.9 (CH_3), 22.3 (C^{13}), 22.1 ($C^{13'}$), 19.2 (C^{19}), 19.1 ($C^{19'}$). **HRMS (ESI$^+$, m/z):** calculated for $C_{46}H_{82}N_6NaO_{10}$, $[M+Na]^+$:901.5985, found 901.5981.

2.3.13 Gadolinium(III) (2,2'-(7,10-bis(carboxylatomethyl)-1,4,7,10-tetraazacyclododecane-1,4-diyl)bis(5-methacrylamidopentanoate)) (26)

Part A - 2,2'-(7,10-Bis(carboxymethyl)-1,4,7,10-tetraazacyclododecane-1,4-diyl)bis-(5-methacrylamidopentanoic acid) (**25**)

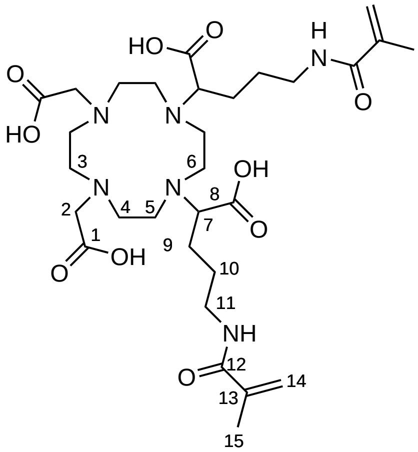

Compound **24** (350 mg, 0.40 mmol, 1.00 equiv.) was stirred in TFA (10.0 mL) and H_2O (1.00 mL) and stirred for 72 hours at room temperature for full deprotection (LC-MS analysis). The reaction mixture was concentrated under reduced pressure and residual TFA was removed by coevaporation with CH_2Cl_2 (5 × 20 mL). The resulting residue was dissolved in deionised water, centrifuged twice and the supernatant was filtered through a syringe filter (220 nm cut-off). The filtrate was lyophilised to afford **25** (280 mg) as a slightly yellow powder. The crude material was used directly in the next step without further purification. **HRMS (ESI$^+$, m/z):** calculated for $C_{30}H_{51}N_6O_{10}$, $[M+H]^+$:655.3661, found 655.3659.

Part B - Gadolinium(III) (2,2'-(7,10-bis(carboxylatomethyl)-1,4,7,10-te-traazacyclododecane-1,4-diyl)bis(5-methacrylamidopentanoate)) (**26**)

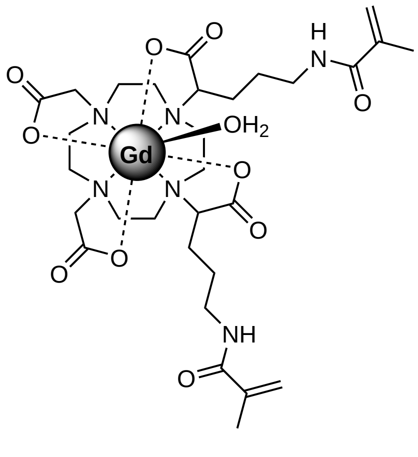

Deprotected ligand **25** (261 mg, 0.40 mmol, 1.00 equiv.) and gadolinium(III) chloride hexahydrate (156 mg, 0.42 mmol, 1.05 equiv.) were stirred in deionized water (10.0 mL) for 24 hours

at room temperature. Over the course of the reaction, the pH was adjusted to 7 by addition of aq. HCl or aq. NaOH. The reaction mixture was lyophilised and the resulting solid was redissolved in deionised water, and centrifuged twice, the supernatant was filtered through a syringe filter (220 nm cut-off) and purified by preparative HPLC [gradient system B: H_2O (25 mM NH_4HCO_3/CH_3CN (see section 2.5)]. The fractions containing the product were combined to yield, after lyophilisation, complex **26** (66.0 mg, 84.2 μmol, 21% over 3 steps) as a white amorphous powder. **IR (ν_{max}/cm^{-1}, neat):** 3273, 3066, 2924, 2868, 1589, 1387, 1316, 1083. **HRMS (ESI$^-$, m/z):** calculated for $C_{30}H_{46}^{158}GdN_6O_{10}$, $[M]^-$:808.2511, found 808.2535. **HPLC [gradient system B, H_2O (25 mM NH_4HCO_3)/CH_3CN)]:** t_R = 5.49 minutes. **Relaxivity (H_20, 60 MHz, pH = 7.4, 37 °C):** r_1 = 5.7 mM^{-1}s^{-1}.

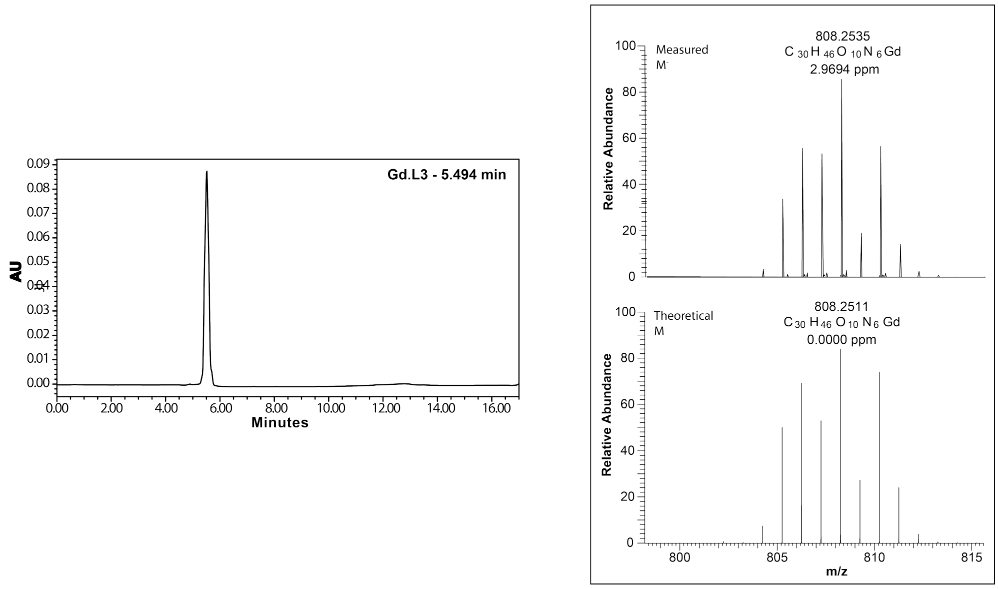

Figure S3. HPLC trace and HRMS spectra of **Gd.L**$_3$.

2.4 Synthesis of Gd.L$_4$

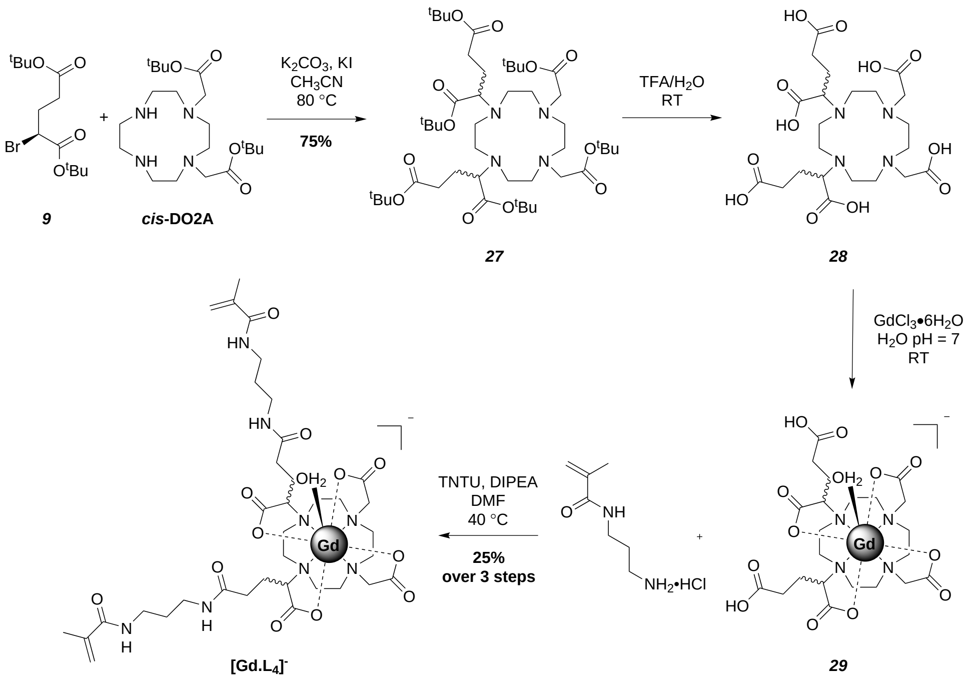

Scheme S4. Synthesis of Gd.L$_4$.

2.4.1 Tetra-*tert*-butyl 2,2'-(7,10-bis(2-(*tert*-butoxy)-2-oxoethyl)-1,4,7,10-tetra-azacyclododecane-1,4-diyl)diglutarate (*27*)

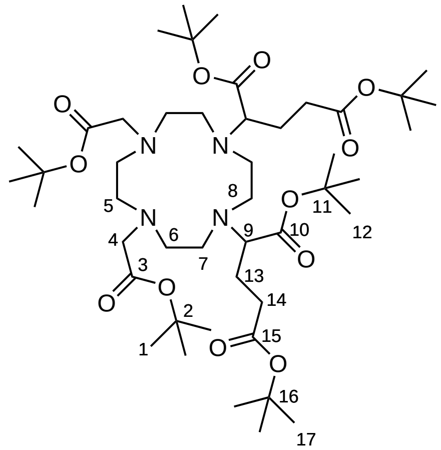

Compound **27** was synthesised following the modification of a literature procedure.[19] ***Cis*-DO2A** (500 mg, 1.25 mmol, 1.00 equiv.), K_2CO_3 (1.04 g, 7.49 mmol, 6.00 equiv.), arm **9** (1.13 g, 3.50 mmol, 2.80 equiv.), and potassium iodide (414 mg, 2.50 mmol, 2.00 equiv.) were dissolved in anhydrous CH_3CN (10 mL) and the reaction was stirred at 80 °C overnight. Potassium salts were removed by centrifugation, and the supernatant was concentrated under reduced

pressure to obtain an oil. The crude residue was purified by column chromatography (silica gel, neat CH_2Cl_2 to CH_2Cl_2/CH_3OH 90:10 v/v, with an increment of 2%) to yield compound **27** (823 mg, 0.93 mmol, 75%) as a yellow amorphous solid. **IR (ν_{max}/cm^{-1}, neat):** 3322, 2974, 2932, 2832, 1718, 1366, 1227, 1147. **^{1}H NMR (400 MHz, $CDCl_3$) δ (ppm):** 3.41 (2H, app. d, J = 10.0 Hz, H^9), 3.34 (2H, d, ^2J = 17.3 Hz, H^{4a}), 3.25-2.85 (4H, 2 × CH_2 cyclen), 2.79 (2H, d, ^2J = 17.3 Hz, H^{4b}), 2.60-2.00 (12H, 6 × CH_2 cyclen), 2.00-1.80 (4H, m, H^{14}), 1.65-1.50 (4H, m, H^{13}), 1.50-1.35 (54H, 18 × CH_3). **^{13}C NMR (101 MHz, $CDCl_3$) δ (ppm):** 174.9, 172.9, 172.8, 172.4, 172.3, 172.0 (CO_2tBu), 82.4, 82.3, 81.9, 81.8, 80.6, 80.5 ($C(CH_3)_3$), 60.2 (C^9), 60.1 ($C^{9'}$), 55.8, 55.7, 55.0 (CH_2CO_2tBu), 52.6, 48.6(2), 48.5(7), 47.4, 47.3, 44.7, 44.5 (CH_2 cyclen), 33.7 (C^{13}), 31.8 (C^{14}), 28.3, 28.2, 28.1, 28.0, 27.9(3), 27.8(6) (CH_3). **HRMS (ESI$^+$, m/z):** calculated for $C_{46}H_{85}N_4O_{12}$, $[M+H]^+$:885.6159, found 885.6158.

Spectral data were in accordance with that reported in the literature.[19]

2.4.2 Gadolinium(III) (2,2'-(7,10-bis(carboxylatomethyl)-1,4,7,10-tetraazacyclododecane-1,4-diyl)bis(5-((3-methacrylamidopropyl)amino)-5-oxopentanoate)) (29)

Part A - 2,2'-(7,10-Bis(carboxymethyl)-1,4,7,10-tetraazacyclododecane-1,4-diyl)-diglutaric acid (**28**)

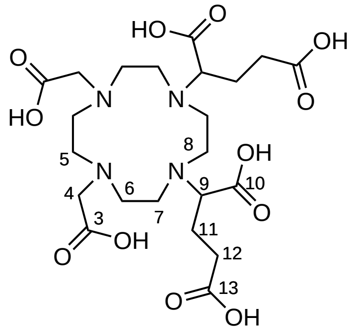

Compound **27** was synthesised following the modification of a literature procedure.[19] Ligand **27** (750 mg, 0.85 mmol, 1.00 equiv.) was stirred in TFA (10.0 mL) and H_2O (1.00 mL) at room temperature for 3 days until complete deprotection (LC-MS analysis). The mixture was concentrated under reduced pressure and residual TFA was removed by successive co-evaporation with CH_2Cl_2. The resulting residue was dissolved in deionised water, centrifuged and filtered through a syringe filter (cut-off 220 nm), and lyophilised to afford a white solid. The crude product was used directly in the next step without further purification. **IR (ν_{max}/cm^{-1}, neat)**: 3084, 2933, 2556, 1945, 1708, 1662, 1390, 1177, 1130. **HRMS (ESI$^+$, m/z):** calculated for $C_{22}H_{37}N_4O_{12}$, $[M+H]^+$:549.2402, found 549.2402.

Part B - Gadolinium(III) (2,2'-(7,10-bis(carboxylatomethyl)-1,4,7,10-tetra-azacyclododecane-1,4-diyl)bis(4-carboxybutanoate)) (**29**)

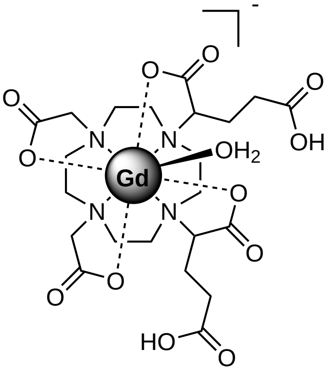

Compound **28** was synthesised following the modification of a literature procedure.[19] Deprotected ligand **28** (400 mg, 0.73 mmol, 1.00 equiv.) and gadolinium(III) chloride hexahydrate (454 mg, 0.88 mmol, 1.20 equiv.) were stirred in water (10.0 mL) at room temperature for 24 hours. Over the course of the reaction, the pH was adjusted to 7 by addition of aq. HCl or aq. NaOH. The reaction mixture was lyophilised and the resulting solid was dissolved in deionised water, centrifuged twice and filtered through a syringe filter (220 nm cut-off). The supernatant was then lyophilised to afford the Gd complex **29** as a a white amorphous solid. The crude product was directly engaged in the next reaction step without further purification. **IR (ν_{max}/cm^{-1}, neat):** 3400, 2980, 2838, 1684, 1600, 1396, 1205, 1128. **HRMS (ESI$^-$, m/z):** calculated for $C_{22}H_{32}{}^{158}GdN_4O_{12}$, [M]$^-$:702.1258, found 702.1246.

Part C - Gadolinium(III) (2,2'-(7,10-bis(carboxylatomethyl)-1,4,7,10-te-traazacyclododecane-1,4-diyl)bis(5-((3-methacrylamidopropyl)amino)-5-oxopentanoate)) (**30**)

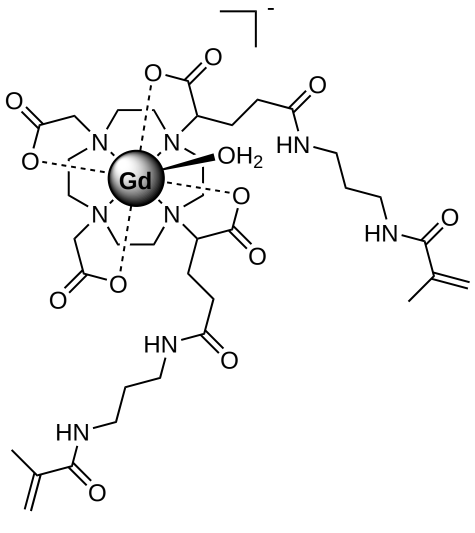

Compound **30** was synthesised following the modification of a literature procedure.[19] Gd complex **29** (200 mg, 0.29 mmol, 1.00 equiv.) and 2-(5-norborene-2,3-dicarboximido)-1,1,3,3-tetramethyluronium tetrafluoroborate (219 mg, 0.60 mmol, 2.10 equiv.) were dissolved in DMF (5.00 mL) at room temperature. The solution was stirred for 30 minutes at 40 °C and DIPEA

(199 μL, 1.14 mmol, 4.00 equiv.) was added, followed by *N*-(3-aminopropyl)methacrylamide hydrochloride (107 mg, 0.60 mmol, 2.10 equiv.). The reaction was stirred for 1 hour at 40 °C and then at room temperature overnight. The desired Gd-complex was precipitated in cold diethylether and collected by filtration. The solid was washed with cold Et_2O and dried under vacuum. The crude residue was dissolved in water and purified by reverse phase preparative HPLC [gradient system B, H_2O (25 mM NH_4HCO_3)/CH_3CN, see section 2.5) to afford, after lyophilisation, complex **30** (68.2 mg, 71.8 μmol, 25% over 3 steps) as a white amorphous solid. **IR (ν_{max}/cm^{-1}, neat):** 3280, 3076, 2928, 2870, 1596, 1538, 1436, 1381, 1085. **HRMS (ESI$^-$, m/z):** calculated for $C_{36}H_{56}{}^{158}GdN_8O_12$, $[M]^-$:950.3259, found 950.3257.**HPLC [gradient system B, H_2O (25 mM NH_4HCO_3)/CH_3CN)]:** t_R = 5.533 minutes. **Relaxivity (H_20, 60 MHz, pH = 7.4, 37 °C):** r_1 = 6.8 mM^{-1}s^{-1}.

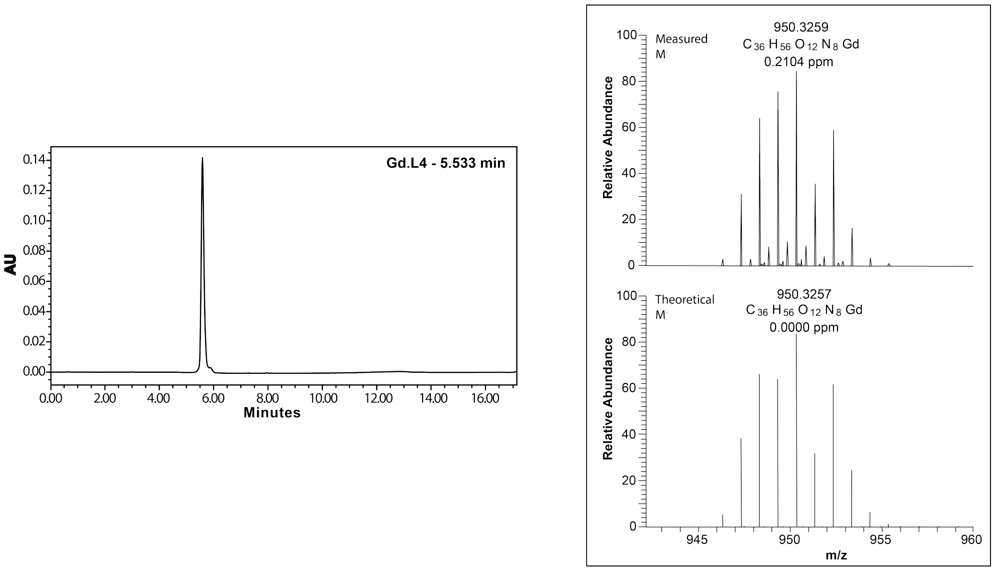

Figure S4. HPLC trace and HRMS spectra of **Gd.L**$_4$.

2.5 High Performace Liquid Chromatography

Table S1. Description of HPLC gradients.

	Gradient A		Gradient B	
Time (min)	Solvent A (%)	Solvent B (%)	Solvent A (%)	Solvent B (%)
0	100	0	100	0
2	100	0	100	0
12	50	50	0	100
13	0	100	0	100
15	0	100	0	100
17	100	0	100	0

3 Characterisation of Linear and Hyperbranched polymeric CAs

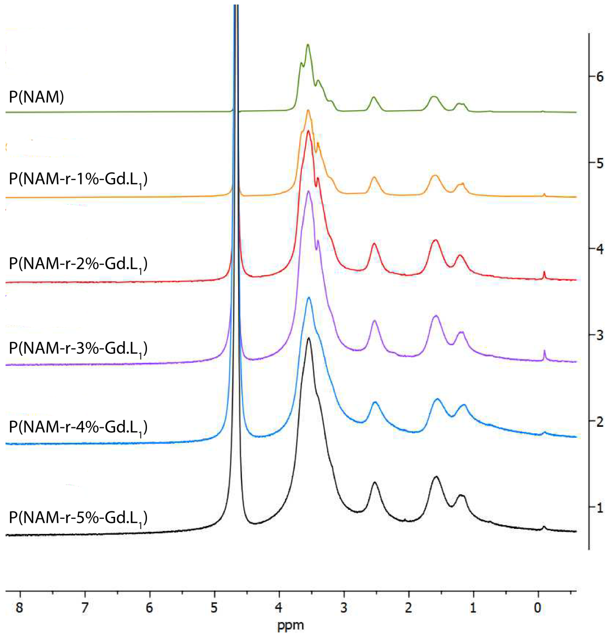

Figure S5. NMR spectra of purified **P(NAM)** and **P(NAM-r-Gd.L$_1$).** polymers showing increased broadening with increasing Gd amount in the polymer formulation. The signal intensity between spectra was normalised to the D_2O solvent peak intensity.

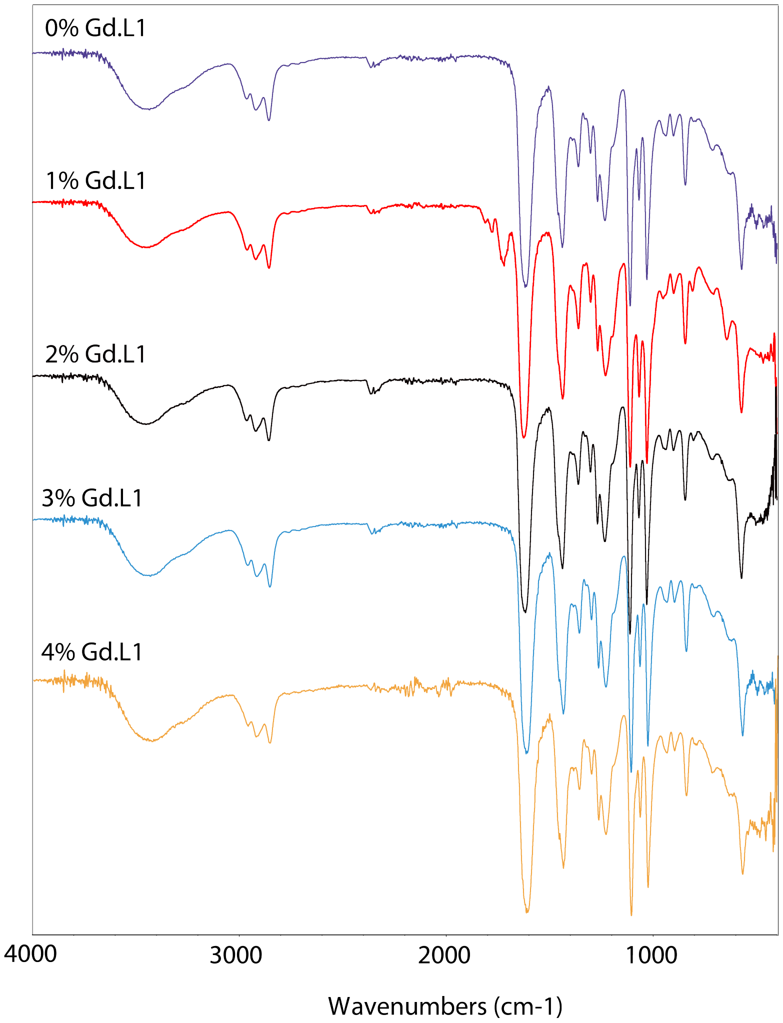

Figure S6. IR spectra of synthesised P(NAM-r-Gd.L_1) linear copolymers.

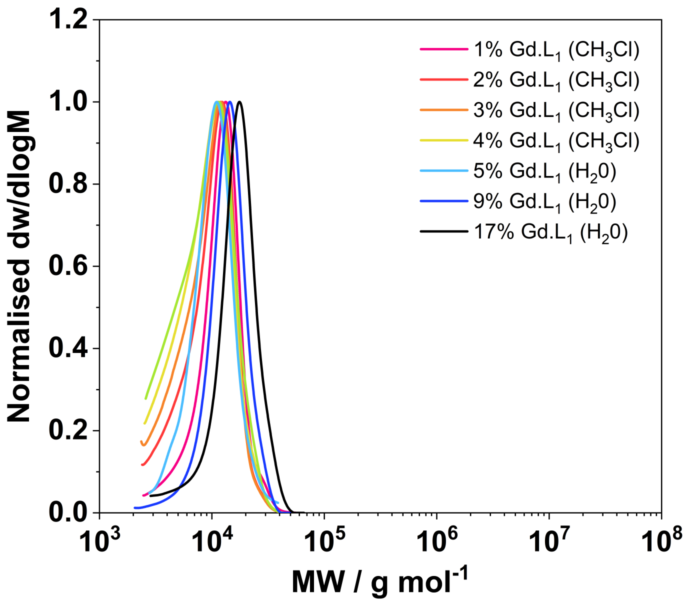

Figure S7. Molecular weight distribution curves of linear copolymers of Gd.L$_1$ obtained by size exclusion chromatography in either chloroform or water.

Table S2. ICP-MS data of linear **P(NAM-r-Gd.L$_1$).** polymers and determination of the Number of Gd atoms per macromolecule.

**ICP-MS analysis of linear P(NAM-r-Gd.L1) copolymers**								
**Entry**	$N^{Gd}_{Theoretical}$ [h]	**[Gd]**[a] **ppb**	**Dilution Factor**[b]	**[Gd]** $_{Sample}$[c] **μg L^{-1}**	**M**$_{Sample}$[d] **mg**	M_n[e]	N^{Gd}[f]	$N^{Gd}_{Corrected}$[g]
1	**Gd.L$_1$ (1)**	35.53 ± 0.44	4,000	142,120	0.98	682.81	0.643	**1**
2	**0.94**	18.27 ± 0.21	400	7,308	1.41	10,260	0.240	**0.374**
3	**1.89**	36.70 ± 0.03	400	14,680	1.15	10,850	0.766	**1.195**
4	**2.84**	41.70 ± 0.68	400	16,680	1.11	10,210	0.879	**1.371**
5	**3.78**	45.65 ± 0.15	400	18,260	0.93	7,580	1.018	**1.588**
6	**4.48**	48.90 ± 0.34	400	19,560	1.83	9,150	0.339	**0.529**
7	**8.58**	14.46 ± 0.14	4,000	57,840	1.53	12,150	1.908	**2.977**
8	**16.67**	15.71 ± 0.03	4,000	62,840	1.05	14,176	5.138	**8.016**

[a] Measured by ICP-MS based on MS signal ^{157}Gd isotope . [b] Required dilution factor to obtain analysable dilute solution from initial sample solution. [c] Gd weight concentration of the initial sample solution: $[Gd]_{Sample}$=[Gd]×Dilution factor. [d] Mass of sample used to prepared the initial solution. [e] Determined by GPC or HRMS. [f] N^{Gd} = ($[Gd]_M/M_w^{Gd}$)/($[Polymer]_M/M_n$ Polymer), where $[Gd]_M$ is the mass fraction of Gd in the non-diluted sample, M_w^{Gd} is the atomic weight of Gd, $[Polymer]_M$ is the mass concentration of polymer in the sample and M_n is the polymer MW. [g] $N^{Gd}_{Corrected}$= N^{Gd} × ($N^{Gd}_{Corrected}$(Gd.L$_1$)/N^{Gd}(Gd.L$_1$)). [h] recalculated from used molar amounts of NAM and **Gd.L$_1$**.

Table S3. ICP-MS data of some hyperbranched polymers and estimation of the number of Gd atoms per macromolecule.

ICP-MS analysis of hyperbranched P(NAM-r-Gd.L_2) copolymers								
**Entry**	$N^{Gd}_{Theoretical}$ [h]	**[Gd]**[a] **ppb**	**Dilution Factor**[b]	**[Gd]** $_{Sample}$ [c] μ**g L**$^{-1}$	**M**$_{Sample}$ [d] **mg**	M_n [e]	N^{Gd} [f]	$N^{Gd}_{Corrected}$ [g]
Gd.L_2	**1**.00	34.99 ± 0.42	4,000	139,960	1.28	807.98	0.44	**1.00**
P(NAM-r-0.9%-Gd.L_2) $[M]_0$=1.00 M	**7.69**	17.82 ± 0.07	400	7,128	1.43	138,600	3.07	**7.00**
P(NAM-r-0.9%-Gd.L_2) $[M]_0$=1.25 M	**22.75**	12.3 ± 0.11	400	4,920	1.11	378,600	9.61	**21.90**
P(NAM-r-0.9%-Gd.L_2) $[M]_0$=1.50 M	**36.17**	15.69 ± 0.11	400	6,276	1.33	592,700	13.37	**30.46**
P(NAM-r-0.9%-Gd.L_2) $[M]_0$=2.00 M	**45.71**	15.02 ± 0.19	400	6,008	1.39	744,700	14.73	**33.55**

[a] Measured by ICP-MS based on ^{157}Gd isotope MS signal. [b] Dilution factor required to obtain analysable dilute solution from mother HNO_3 sample solution. [c] Gd weight concentration of the mother sample solution: $[Gd]_{Sample}$=[Gd]*Dilution factor. [d] Mass of sample used to prepared the nitric mother solution. [e] Determine by GPC or HRMS. [f] N_{Gd} = $([Gd]_W/M^{Gd}_w)/([Polymer]_W/M_w$ Polymer), where $[Gd]_W$ is the mass fraction of Gd in the non-diluted sample, M^{Gd}_w is Gd atomic mass, $[Polymer]_W$ is the mass concentration of polymer in the sample and M_w is hyperbranched system MW average number of the polymer. [g] $N^{Corrected}_{Gd} = N_{Gd} \times (N^{Corrected}_{Gd}(Gd.L_1)/N_{Gd}(Gd.L_1))$. [h] $N^{Theoretical}_{Gd}=((M^{HBP}_w/M^{LP}_w)-1)\times$ [(mol % #/0.9) for (mol % Gd.L_X%) < 0.9], M^{HBP}_W being the GPC M_w of the hyperbranched polymer and M^{LP}_w is the expected MW of a linear polymer analogue.

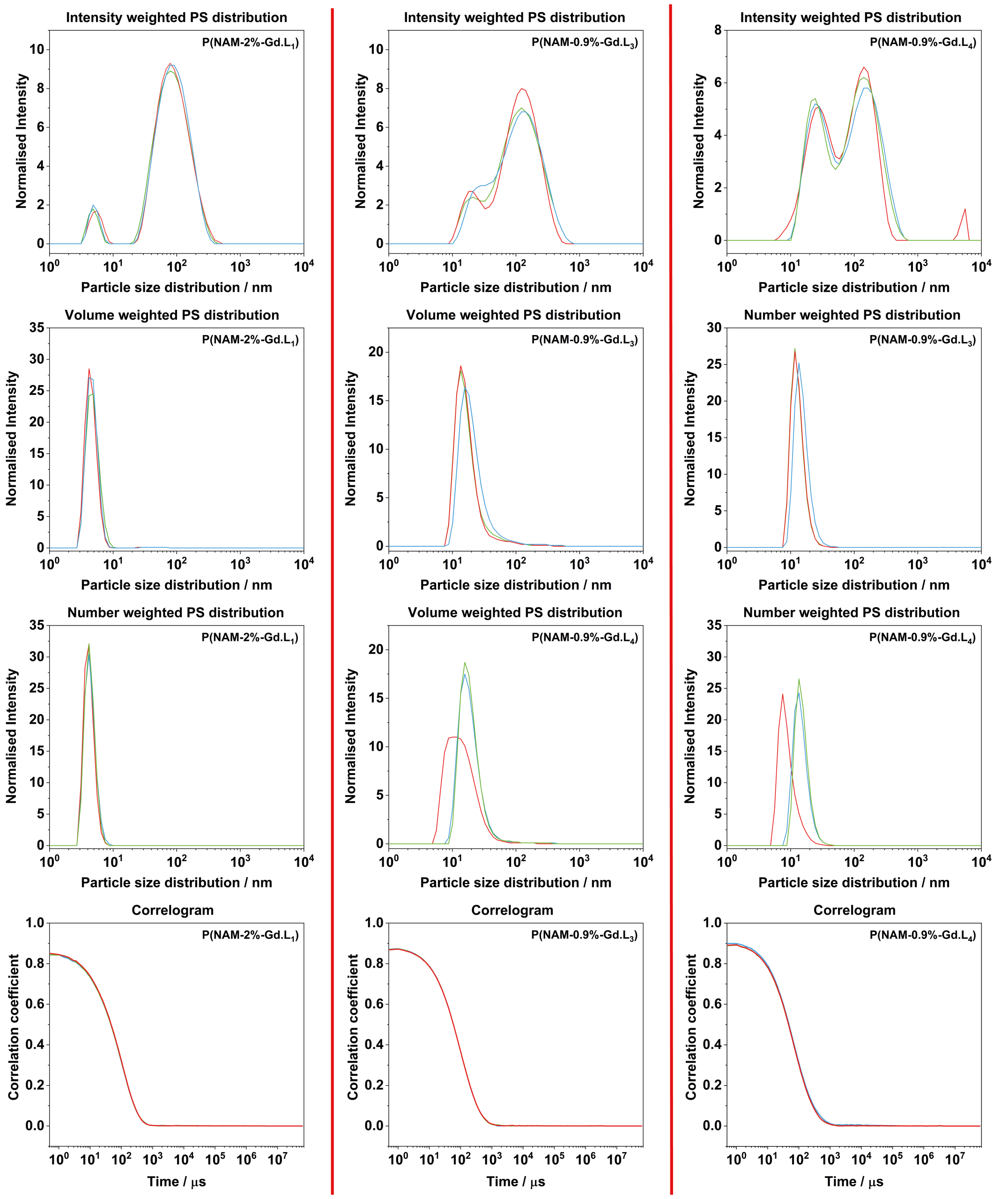

Figure S8. Representative examples of DLS particle size distributions (intensity/volume and number weighted) with associated correlograms for linear and hyperbranched polymeric CAs, obtained in deionized water at 25 °C after filtration with a syringe filter (220 nm cut-off). Each polymer sample was analysed three times and each measurement is associated with a distinctive colour, either blue, red or green.

Table S4. Hydrodynamic diameter and polydispersities values for representative examples of our polymeric CAs determined by DLS measurements.

			DLS results - Determination of hydrodynamic diameter D_h by :		
		PDI	Intensity	Volume	Number
P(NAM-2%-Gd.L_1)	A	0.34	61.8 nm	4.6 nm	4.2 nm
	B	0.35	61.7 nm	4.9 nm	4.4 nm
	C	0.35	62.3 nm	4.7 nm	4.4 nm
P(NAM-Gd.L_3)	A	0.51	67.0 nm	21.5 nm	13.1 nm
	B	0.51	67.2 nm	20.3 nm	13.2 nm
	C*	0.51	67.0 nm	26.2 nm	15.3 nm
P(NAM-Gd.L_4)	A*	0.50	52.1 nm	15.9 nm	9.1 nm
	B	0.48	51.5 nm	21.6 nm	14.7 nm
	C	0.48	51.8 nm	21.6 nm	15.4 nm

*theses measurments were disgarded for the determination of the average D_h.

4 Relaxometry Study Data

4.1 Acquisition of NMRD Profiles

To obtain NMRD profiles with the relaxivity on the y axis, it is necessary to calculate the relaxivity r_1 from the observed measured relaxation rates according to Equation 1. With [Gd], the concentration of Gd determined by measurement of the bulk magnetic susceptibility shifts of ^{t}BuOH signals in the samples (Evans method).[24] $R_{1,d}$ the diamagnetic contribution of the solvent, determined empirically at 0.28 s^{-1} for a set temperature of 37 °C.

$$r_1 = \frac{R_{1,obs} - R_{1,d}}{[Gd]} \tag{1}$$

$$[Gd] = \frac{\Delta\delta_{ppm} 3T}{4\pi \times 10^3} \left(\frac{2.84}{\mu_{eff}(Gd)}\right)^2 \tag{2}$$

[Gd] of the different dilutions was determined by NMR BMS measurements following equation 2, In practice, the Gd concentrations (expressed in mM) were determined by measuring the BMS peak shift of a reference molecule added to an aqueous solution of a paramagnetic compound, 1% *tert*-butanol in this case. As water gives large solvent peaks, the use of a reference proton signal leads to more precise measurements. The BMS shift Δδppm was measured by ^{1}H NMR with a coaxial insert containing a reference diamagnetic solution of tert-butanol in H_2O (1% v/v) where T is the temperature of the sample in the NMR spectrometer in Kelvin and µeff (Gd) is the effective magnetic moment of a gadolinium atom.

4.2 Acquisition of Variable Temperature relaxometry profiles

VT ^{1}H NMR Measurements. Variable temperature ^{1}H NMR profile represents the variation of the water proton relaxation rate of a paramagnetic compound in non-deuterated aqueous solutions, depending on the temperature at a fixed magnetic field intensity (20 or 30 MHz) using an inversion recovery method with a 90° pulse. The profiles were obtained by measuring the relaxation rate at different tem-perature from 5 to 75 °C (12 to 16 acquisition points). The relaxometer operates under computer control with an absolute uncertainty in 1/T_1 values obtained with an error not exceeding ± 1%. The temperature was set and controlled with a Stelar VTC-91 airflow heater equipped with a copper-constantan thermocouple (error ± 0.1 °C) and an air/liquid nitrogen cooling system for temperatures lower than room temperature. Data points were obtained by measurements at high magnetic fields (20 or 30 MHz) on a Stelar relaxometer with a Spinmaster console connected to a Bruker WP-80 magnet at 80 MHz (2 T) adapted to variable-field measurements. The temperature dependence of τ_V and τ_R has been consid-

ered through their activation energies E_V, set to 1.0 kJ mol^{-1}, and E_R, fixed to 18.0 kJ mol^{-1}. ICP-MS Measurements. The exact concentration

4.3 NMRD Profiles Discrete Complexes

Table S5. NMRD raw data Gd.L$_1$.

**NMRD Data - Gd.L$_1$ - [Gd^{3+}] = 21.81 mM**					
**25 °C**			**37 °C**		
**Magnetic Field MHz**	**R$_1$^{a} s^{-1}**	**r_1^{b} mM^{-1} s^{-1}**	**Magnetic Field MHz**	**R$_1$^{a} s^{-1}**	**r_1^{b} mM^{-1} s^{-1}**
0.00997	292.58	13.40367	0.00997	240.59	11.01881
0.01436	281.35	13.42853	0.01436	240.02	10.99266
0.0207	299.32	13.35322	0.0207	237.7	10.88624
0.02973	291.07	13.3344	0.02973	238.9	10.94128
0.04287	296.69	13.3922	0.04287	238.05	10.90229
0.06162	292.52	13.40092	0.06162	237.58	10.88073
0.08854	286.05	13.30413	0.08854	240.02	10.99266
0.12749	290.03	13.2867	0.12749	237.1	10.85872
0.18317	290.3	13.29908	0.18317	233.09	10.67477
0.26376	295.82	13.25229	0.26376	237.13	10.86009
0.37932	303.17	13.38945	0.37932	229.74	10.8211
0.54549	301.3	13.30367	0.54549	227.74	10.72936
0.7845	276.41	12.96193	0.7845	236.39	10.82615
1.1292	263.9	12.48807	1.1292	228.64	10.47064
1.6235	271.44	11.93394	1.6235	214.77	9.8344
2.3363	240.18	11.02	2.3363	204.58	9.36697
3.3598	202.91	10.29037	3.3598	185.91	8.51055
3.3598	229.96	10.23119	3.3598	184.84	8.46147
4.8321	193.83	8.87385	4.8321	157.83	7.22248
6.9526	165.96	7.59541	6.9526	141.13	6.45642
9.9994	156.41	7.15734	9.9994	131.28	6.00459
21	141.62	6.4789	21	111.86	5.11376
31	135.98	6.22018	31	108.61	4.96468
41	132.96	6.08165	41	105.56	4.82477
49	127.75	5.84266	49	102.65	4.69128
61	123.4	5.64312	61	98.75	4.51239
72	114.61	5.63991	72	95.12	4.34587

^{a} Measured longitudinal relaxation rate. ^{b} r_1 calculated from the following equation: r_1 = (R$_1$ - $^{H_2O}r_1$) / [Gd^{3+}], $^{H_2O}r_1$ being the diamagnetic longitudinal relaxation rate of water (0.38 s^{-1} at 25°C, 0.28 s^{-1} at 37°C).

Table S6. NMRD raw data Gd.L_2.

NMRD Data - Gd.L_2 - $[Gd^{3+}]$ = 5.41 mM					
25 °C			37 °C		
Magnetic Field MHz	R_1[a] s^{-1}	r_1[b] $mM^{-1}\ s^{-1}$	Magnetic Field MHz	R_1[a] s^{-1}	r_1[b] $mM^{-1}\ s^{-1}$
0.00997	78.581	14.48167	0.00997	68.123	12.545
0.01436	79.607	14.67167	0.01436	67.235	12.38056
0.0207	79.068	14.57185	0.0207	68.013	12.52463
0.02973	79.465	14.64537	0.02973	68.22	12.56296
0.04287	80.281	14.79648	0.04287	66.669	12.27574
0.06162	80.037	14.7513	0.06162	67.434	12.41741
0.08854	80.233	14.78759	0.08854	67.685	12.46389
0.12749	79.87	14.72037	0.12749	67.948	12.51259
0.18317	81.388	14.80148	0.18317	67.084	12.35259
0.26376	79.722	14.69296	0.26376	67.459	12.42204
0.37932	81.011	14.73167	0.37932	66.186	12.1863
0.54549	78.743	14.51167	0.54549	67.044	12.34519
0.7845	76.814	14.15444	0.7845	65.045	11.975
1.1292	74.488	13.7237	1.1292	62.198	11.44778
1.6235	74	13.13333	1.6235	62.412	11.18741
2.3363	65.745	12.10463	2.3363	57.488	10.57556
3.3598	60.68	11.16667	3.3598	52.502	9.65222
3.3598	59.194	10.89148	3.3598	52.47	9.6463
4.8321	53.312	9.80222	4.8321	45.462	8.34852
6.9526	50.319	9.24796	6.9526	41.667	7.64574
9.9994	47.386	8.70481	9.9994	39.184	7.18593
21	45.55	8.36481	21	36.07	6.60926
31	44.8	8.22593	31	36.27	6.6463
41	43.74	8.02963	41	35.61	6.52407
49	42.24	7.75185	49	34.56	6.32963
61	41.36	7.58889	61	33.34	6.1037
72	37.91	7.45	72	32.69	5.98333

[a] Measured longitudinal relaxation rate. [b] Relaxivity calculated from the following equation: $r_1 = (R_1 - {}^{H_2O}r_1) / [Gd^{3+}]$, ${}^{H_2O}r_1$ being the diamagnetic longitudinal relaxation rate of water (0.38 s^{-1} at 25°C, 0.28 s^{-1} at 37°C).

Table S7. NMRD raw data Gd.L_3.

**NMRD Data - Gd.L_3 - [Gd^{3+}] = 7.76 mM**					
**25 °C**			**37 °C**		
**Magnetic Field MHz**	**R_1[a] s^{-1}**	r_1[b] **mM^{-1} s^{-1}**	**Magnetic Field MHz**	**R_1[a] s^{-1}**	r_1[b] **mM^{-1} s^{-1}**
0.00997	127	16.31701	0.00997	103	13.23711
0.0144	128	16.44588	0.0144	104	13.36598
0.0207	129	16.57474	0.0207	103	13.23711
0.0297	126	16.18814	0.0297	104	13.36598
0.0429	126	16.18814	0.0429	103	13.23711
0.0616	128	16.44588	0.0616	103	13.23711
0.0885	126	16.18814	0.0885	102	13.10825
0.127	130	16.70361	0.127	104	13.36598
0.183	127	16.31701	0.183	102	13.10825
0.264	128	16.44588	0.264	101	12.97938
0.379	126	16.18814	0.379	103	13.23711
0.545	125	16.05928	0.545	100	12.85052
0.785	124	15.93041	0.785	98	12.59278
1.13	119	15.28608	1.13	97.2	12.48969
1.62	113	14.51289	1.62	93	11.94845
2.34	102	13.09536	2.34	84.5	10.81443
3.36	89.9	11.53608	3.36	73.9	9.48711
4.83	79.1	10.14433	4.83	65.3	8.37887
6.95	68.4	8.76546	6.95	56.9	7.29639
10	62.8	8.04381	10	49.6	6.35567
21	59.4	7.60567	21	45.7	5.85309
31	58	7.42526	31	44	5.63402
41	56.9	7.28351	41	43.4	5.5567
49	56.4	7.21907	49	42.9	5.49227
61	55.3	7.07732	61	42.6	5.45361
72	55.1	7.05155	72	41.7	5.33763

[a] Measured longitudinal relaxation rate. [b] Relaxivity calculated from the following equation: r_1 = (R_1 - ${}^{H_2O}r_1$) / [Gd^{3+}], ${}^{H_2O}r_1$ being the diamagnetic longitudinal relaxation rate of water (0.38 s^{-1} at 25°C, 0.28 s^{-1} at 37°C).

Table S8. NMRD raw data Gd.L_4.

**NMRD Data - Gd.L_4 - [Gd^{3+}] = 7.51 mM**					
**25 °C**			**37 °C**		
**Magnetic Field MHz**	**$R_1{}^a$ s^{-1}**	**$r_1{}^b$ mM^{-1} s^{-1}**	**Magnetic Field MHz**	**$R_1{}^a$ s^{-1}**	**$r_1{}^b$ mM^{-1} s^{-1}**
0.00997	139	18.45806	0.00997	109	14.4767
0.0144	140	18.59121	0.0144	108	14.34354
0.0207	139	18.45806	0.0207	108	14.34354
0.0297	137	18.19174	0.0297	109	14.4767
0.0429	140	18.59121	0.0429	109	14.4767
0.0616	142	18.85752	0.0616	109	14.4767
0.0885	141	18.72437	0.0885	109	14.4767
0.127	138	18.3249	0.127	110	14.60985
0.183	139	18.45806	0.183	108	14.34354
0.264	139	18.45806	0.264	109	14.4767
0.379	139	18.45806	0.379	109	14.4767
0.545	135	17.92543	0.545	105	13.94407
0.785	133	17.65912	0.785	104	13.81092
1.13	126	16.72703	1.13	102	13.54461
1.62	120	15.9281	1.62	96.8	12.8522
2.34	110	14.59654	2.34	89.7	11.90679
3.36	96	12.73236	3.36	76.1	10.09587
4.83	84.8	11.24101	4.83	65.2	8.64447
6.95	75.5	10.00266	6.95	60.4	8.00533
10	71.3	9.44341	10	54	7.15313
21	69	9.13715	21	53.5	7.08655
31	69	9.13715	31	52.5	6.9534
41	67.8	8.97736	41	51.8	6.86019
49	66.5	8.80426	49	50.9	6.74035
61	65	8.60453	61	49.7	6.58056
72	64.1	8.48469	72	49.5	6.55393

[a] Measured longitudinal relaxation rate. [b] Relaxivity calculated from the following equation: r_1 = (R_1 - ${}^{H_2O}r_1$) / [Gd^{3+}], ${}^{H_2O}r_1$ being the diamagnetic longitudinal relaxation rate of water (0.38 s^{-1} at 25°C, 0.28 s^{-1} at 37°C).

4.4 Fitted NMRD Profiles of Gd.L$_{1-4}$

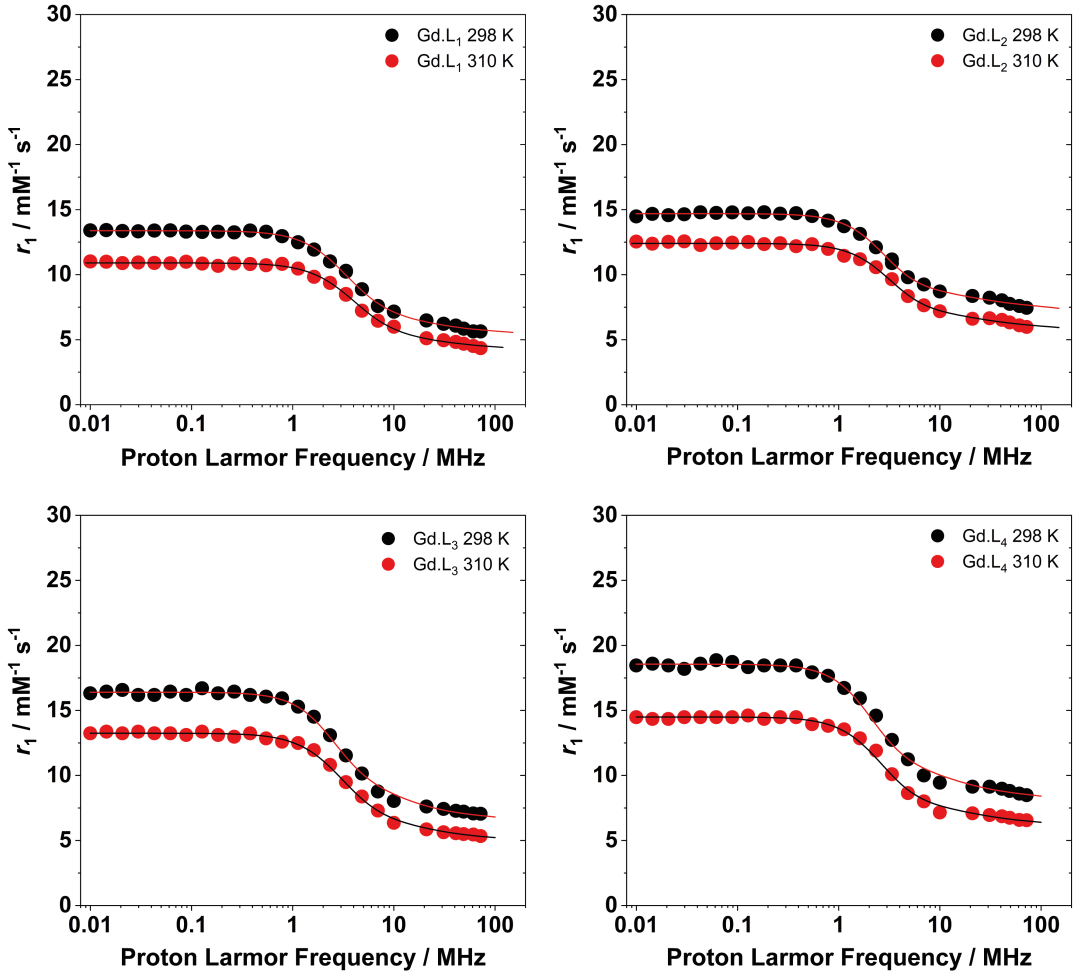

Figure S9. Fitted NMRD profiles of synthesised **Gd.L**$_{1-4}$ complexes at 25 and 37 °C. Solid red and black lines correspond to the fitting of data sets.

4.5 ^{17}O NMR Raw Data Gd Complexes

Table S9. Data ^{17}O NMR Gd.L$_1$.

T K	10^3/T K^{-1}	^{t}BuOH dia ppm	^{t}BuOH para ppm	χ^a Hz	^{17}O dia ppm	^{17}O para ppm	Shift^b Hz	Real Shift Hz	$\Delta\omega_r{}^d$ rad/s	dw1/2 dia	dw1/2 para	R_2p^e s^{-1}	Conc mM	ln(1/T_{2r})
276.6	3.615	0.8707	3.13	153.16	3.89	6.07	147.7	-5.4	-1.01E+5	100.18	180.62	252.58	19.06	13.53205
278.8	3.587	0.8988	3.14	152.01	3.82	6.19	160.9	8.9	1.66E+5	91.72	178.96	273.93	19.06	13.61320
284.4	3.516	0.9741	3.16	148.45	3.60	5.55	132.4	-16.1	-3.02E+5	74.14	200.11	395.55	19.00	13.98058
289.7	3.452	1.0646	3.19	144.27	3.33	5.38	139.2	-5.1	-9.55E+4	58.38	223.49	518.45	18.80	14.25115
295.5	3.384	1.1219	3.21	141.57	3.14	5.04	128.5	-13.1	-2.46E+5	50.42	241.507	600.01	18.82	14.39727
300.0	3.333	1.1978	3.24	138.12	2.90	4.60	115.4	-22.7	-4.26E+5	42.13	251.77	658.27	18.64	14.48993
303.2	3.298	1.2630	3.25	135.02	2.66	4.13	99.4	-35.7	-6.68E+5	36.32	254.34	684.58	18.42	14.52913
308.5	3.242	1.3265	3.28	132.09	2.44	3.64	81.3	-50.7	-9.51E+5	32.01	232.70	630.17	18.33	14.4463
313.9	3.186	1.3888	3.29	129.19	2.21	3.59	93.6	-35.6	-6.67E+5	28.11	201.85	545.54	18.24	14.30210
319.3	3.132	1.4482	3.32	126.57	1.99	3.08	73.6	-53.0	-9.92E+5	25.04	163.95	436.18	18.18	14.07837
324.7	3.079	1.5066	3.34	124.26	1.76	2.98	82.4	-41.9	-7.85E+5	22.31	133.85	350.24	18.15	13.85892
330.1	3.029	1.5639	3.36	121.52	1.54	2.66	76.1	-45.4	-8.51E+5	20.15	106.73	271.86	18.04	13.60561
335.4	2.982	1.6198	3.38	119.21	1.32	2.41	73.9	-45.3	-8.49E+5	18.91	85.42	208.84	17.98	13.34190
340.4	2.938	1.6748	3.40	117.04	1.09	2.17	73.3	-43.7	-8.19E+5	17.00	70.49	167.96	17.92	13.12403
345.8	2.892	1.7282	3.43	115.02	0.86	1.94	72.7	-42.3	-7.93E+5	15.42	56.39	128.65	17.89	12.85738
351.5	2.845	1.7798	3.45	113.23	0.64	1.70	72.0	-41.2	-7.72E+5	14.18	46.36	101.05	17.90	12.61589
													**PM^f**	**3.35E-4**
													q	**1.0**

^{a} χ = (^{t}BuOH$_{par}$-^{t}BuOH$_{dia}$) × 67.8. ^{b} Shift = ($^{17}O_{para}$-$^{17}O_{d}ia$) × 67.8. ^{c} Real-Shift = (($^{17}O_{para}$-$^{17}O_{d}ia$)-($^{17}O_{para}$-$^{17}O_{d}ia$)) × 67.8. ^{d} $\Delta\omega_r$ = (Real-Shift × 2π)/PM. ^{e} R_2p = (dw1/2$_{par}$-dw1/2$_{dia}$) × π. ^{f} PM = (q × 0.001 × $^{310}[Gd]$/(55.56+(q × 0.001 × $^{310}[Gd]$))).

Table S10. Data ^{17}O NMR Gd.L_2.

T set K	T K	10^3/T K^{-1}	^{t}BuOH dia ppm	^{t}BuOH para ppm	χ^a Hz	^{17}O dia ppm	^{17}O para ppm	Shift^b Hz	Real Shift Hz	$\Delta\omega_r{}^d$ rad/s	dw1/2 dia	dw1/2 para	R_2p^e s^{-1}	Conc mM	ln(1/T_{2r})
278	276.6	3.615329	0.8719	2.7989	130.6506	3.9269	5.8404	129.7	-0.9	-1.97E+4	99.93	179.04	248.41	16.26	13.654
280	278.8	3.586801	0.8989	2.8150	129.9116	3.8170	5.6022	121.0	-8.9	-1.91E+5	91.97	180.29	277.32	16.29	13.764
285	284.4	3.516174	0.9738	2.8535	127.4437	3.5702	5.3746	122.3	-5.1	-1.10E+5	73.56	197.20	388.23	16.30	14.101
290	289.7	3.451847	1.0468	2.8854	124.6571	3.3759	4.9383	105.9	-18.7	-4.03E+5	60.70	228.22	526.01	16.24	14.404
295	295.5	3.384294	1.1197	2.9203	122.0807	3.1374	4.6435	102.1	-20.0	-4.30E+5	50.67	239.83	593.96	16.23	14.526
305	303.2	3.297901	1.2649	2.9677	115.4498	2.6770	4.1770	101.7	-13.7	-2.96E+5	36.40	250.48	672.20	15.75	14.650
310	308.5	3.241491	1.3274	2.9911	112.7989	2.4540	3.8330	93.5	-19.3	-4.15E+5	31.81	226.48	611.26	15.65	14.554
315	313.9	3.186235	1.3887	3.0157	110.3106	2.2220	3.5090	87.3	-23.1	-4.96E+5	28.03	196.14	527.86	15.57	14.408
320	319.3	3.131851	1.4480	3.0457	108.3241	2.0030	3.1580	78.3	-30.0	-6.46E+5	24.99	162.47	431.67	15.56	14.207
325	324.7	3.07941	1.5060	3.0666	105.8087	1.7750	2.9480	79.5	-26.3	-5.66E+5	22.35	132.95	347.26	15.46	13.989
330	330.1	3.029385	1.5636	3.0922	103.6391	1.5520	2.6760	76.2	-27.4	-5.90E+5	20.20	107.41	273.86	15.39	13.752
335	335.4	2.981636	1.6199	3.1167	101.483	1.3280	2.4240	74.3	-27.2	-5.85E+5	18.33	85.82	211.91	15.31	13.495
														**PM^f**	**2.92E-4**
														q	**1.0**

^{a} χ = (^{t}BuOH$_{par}$-^{t}BuOH$_{dia}$) × 67.8. ^{b} Shift = ($^{17}O_{para}$-$^{17}O_{d}ia$) × 67.8. ^{c} Real-Shift = (($^{17}O_{para}$-$^{17}O_{d}ia$)-($^{17}O_{para}$-$^{17}O_{d}ia$)) × 67.8. ^{d} $\Delta\omega_r$ = (Real-Shift × 2π)/PM. ^{e} R_2p = (dw1/2$_{par}$-dw1/2$_{dia}$) × π. ^{f} PM = (q × 0.001 × $^{310}[Gd]$/(55.56+(q × 0.001 × $^{310}[Gd]$))).

Table S11. Data ^{17}O NMR Gd.L_3.

T set K	T K	10^3/T K^{-1}	^{t}BuOH dia ppm	^{t}BuOH para ppm	χ^a Hz	^{17}O dia ppm	^{17}O para ppm	Shift^b Hz	Real Shift Hz	$\Delta\omega_r{}^d$ rad/s	dw1/2 dia	dw1/2 para	R_2p^e s^{-1}	Conc mM	ln(1/T_{2r})
278	276.6	3.615329	0.8735	2.7346	126.1826	3.8658	5.7538	128.0	1.8	4.0877E+4	98.77	190.24	287.22	15.70	13.8397392
280	278.8	3.586801	0.8998	2.7493	125.3961	3.8191	5.7946	133.9	8.5	1.9147E+5	91.22	197.29	333.06	15.73	13.9878274
285	284.4	3.516174	0.9755	2.7831	122.5553	3.5941	5.4263	124.2	1.7	3.7382E+4	73.14	213.71	441.39	15.68	14.2694337
290	289.7	3.451847	1.0486	2.8142	119.7077	3.3687	5.0131	111.5	-8.2	-1.8417E+5	60.62	228.88	528.34	15.60	14.4492385
295	295.5	3.384294	1.1210	2.8445	116.8533	3.1446	4.5745	96.9	-19.9	-4.4615E+5	50.25	245.05	611.67	15.53	14.5957015
300	300.0	3.333333	1.1665	2.8694	115.4566	2.9763	4.3310	91.8	-23.6	-5.2912E+5	44.95	241.40	616.85	15.58	14.6041361
305	303.2	3.297901	1.2512	2.9039	112.0531	2.6942	3.8449	78.0	-34.0	-7.6283E+5	37.15	224.82	589.28	15.28	14.5584132
310	308.5	3.241491	1.3212	2.9349	109.4089	2.4513	3.5668	75.6	-33.8	-7.5705E+5	32.01	192.81	504.91	15.18	14.4038895
315	313.9	3.186235	1.3854	2.9622	106.907	2.2201	3.1795	65.0	-41.9	-9.3819E+5	28.44	157.48	405.19	15.09	14.1838506
320	319.3	3.131851	1.4465	2.9889	104.5747	1.9931	2.9487	64.8	-39.8	-8.9169E+5	25.21	130.78	331.49	15.02	13.9831024
325	324.7	3.07941	1.5056	3.0154	102.3644	1.7642	2.6883	62.7	-39.7	-8.9002E+5	22.39	103.16	253.62	14.95	13.7153338
330	330.1	3.029385	1.5630	3.0423	100.2965	1.5440	2.4684	62.7	-37.6	-8.4321E+5	20.15	84.50	202.06	14.89	13.4880651
335	335.4	2.981636	1.6186	3.0689	98.33034	1.3190	2.2256	61.5	-36.9	-8.2620E+5	18.16	68.33	157.53	14.83	13.2391454
340	340.4	2.937751	1.6731	3.0983	96.62856	1.0931	2.0004	61.5	-35.1	-7.8699E+5	17.17	56.14	122.37	14.80	12.9865203
345	345.8	2.891781	1.7266	3.1268	94.93356	0.8681	1.7399	59.1	-35.8	-8.0295E+5	15.59	46.44	96.87	14.77	12.7528649
350	351.5	2.84486	1.7784	3.1567	93.44874	0.6440	1.5153	59.1	-34.4	-7.7043E+5	14.35	39.39	78.63	14.78	12.5442027
														**PM^f**	**2.80E-4**
														q	**1.0**

^{a} χ = (^{t}BuOH$_{par}$-^{t}BuOH$_{dia}$) × 67.8. ^{b} Shift = ($^{17}O_{para}$-$^{17}O_{d}ia$) × 67.8. ^{c} Real-Shift = (($^{17}O_{para}$-$^{17}O_{d}ia$)-($^{17}O_{para}$-$^{17}O_{d}ia$)) × 67.8. ^{d} $\Delta\omega_r$ = (Real-Shift × 2π)/PM. ^{e} R_2p = (dw1/2$_{par}$-dw1/2$_{dia}$) × π. ^{f} PM = (q × 0.001 × $^{310}[Gd]$/(55.56+(q × 0.001 × $^{310}[Gd]$))).

Table S12. Data ^{17}O NMR Gd.L_4.

**T set K**	**T K**	**10^3/T K^{-1}**	**^{t}BuOH dia ppm**	**^{t}BuOH para ppm**	**χ^a Hz**	**^{17}O dia ppm**	**^{17}O para ppm**	**Shift^b Hz**	**Real Shift Hz**	**$\Delta\omega_r{}^d$ rad/s**	**dw1/2 dia**	**dw1/2 para**	**R_2p^e s^{-1}**	**Conc mM**	**ln(1/T_{2r})**
278	276.6	3.615329	0.8725	2.6792	122.4943	3.9031	5.6204	116.4	-6.1	-1.4188E+5	99.51	182.86	261.72	15.24	13.7902108
280	278.8	3.586801	0.8994	2.6932	121.6196	3.8571	5.4527	108.2	-13.4	-3.1455E+5	92.05	176.80	266.12	15.25	13.8068679
285	284.4	3.516174	0.9752	2.7304	119.0026	3.5728	5.2121	111.1	-7.9	-1.8394E+5	73.64	176.72	323.67	15.22	14.0026676
290	289.7	3.451847	1.0478	2.7631	116.2973	3.3654	4.9835	109.7	-6.6	-1.5426E+5	60.70	186.09	393.72	15.15	14.1985911
295	295.5	3.384294	1.1211	2.7955	113.5243	3.1364	4.4284	87.6	-25.9	-6.0688E+5	50.59	186.42	426.51	15.09	14.2785663
300	300.0	3.333333	1.1983	2.8288	110.5479	2.8839	4.2764	94.4	-16.1	-3.7771E+5	41.88	189.66	464.03	14.92	14.3628869
305	303.2	3.297901	1.2637	2.8587	108.141	2.6580	3.8557	81.2	-26.9	-6.3053E+5	36.41	173.65	430.93	14.75	14.2888934
310	308.5	3.241491	1.3264	2.8896	105.985	2.4362	3.5435	75.1	-30.9	-7.2353E+5	31.84	149.27	368.73	14.71	14.1330046
315	313.9	3.186235	1.3877	2.9182	103.7679	2.2172	3.2723	71.5	-32.2	-7.5448E+5	28.28	131.86	325.24	14.65	14.0075064
320	319.3	3.131851	1.4474	2.9402	101.2118	1.9925	2.9575	65.4	-35.8	-8.3764E+5	25.13	107.39	258.30	14.54	13.7770472
325	324.7	3.07941	1.5059	2.9726	99.44226	1.7681	2.7158	64.3	-35.2	-8.2367E+5	22.47	88.48	207.27	14.53	13.5569684
330	330.1	3.029385	1.5633	2.9982	97.28622	1.5448	2.4731	62.9	-34.3	-8.0399E+5	20.23	72.56	164.32	14.45	13.324732
335	335.4	2.981636	1.6360	3.0674	97.04892	1.2565	2.1969	63.8	-33.3	-7.7923E+5	18.08	57.97	125.25	14.64	13.0532879
340	340.4	2.937751	1.6801	3.0884	95.48274	1.0692	1.9749	61.4	-34.1	-7.9764E+5	17.08	49.26	101.05	14.62	12.8385073
345	345.8	2.891781	1.7303	3.1118	93.6657	0.8545	1.7537	61.0	-32.7	-7.6543E+5	15.26	41.80	83.34	14.57	12.6458152
350	351.5	2.84486	1.7808	3.1396	92.12664	0.6365	1.5216	60.0	-32.1	-7.5178E+5	14.01	35.16	66.41	14.57	12.4188021
														**PM^f**	**2.68E-4**
														q	**1.0**

^{a} χ = (^{t}BuOH$_{par}$-^{t}BuOH$_{dia}$) × 67.8. ^{b} Shift = ($^{17}O_{para}$-$^{17}O_{d}ia$) × 67.8. ^{c} Real-Shift = (($^{17}O_{para}$-$^{17}O_{d}ia$)-($^{17}O_{para}$-$^{17}O_{d}ia$)) × 67.8. ^{d} $\Delta\omega_r$ = (Real-Shift × 2π)/PM. ^{e} R_2p = (dw1/2_{par}-dw1/2_{dia}) × π. ^{f} PM = (q × 0.001 × $^{310}[Gd]$/(55.56+(q × 0.001 × $^{310}[Gd]$))).

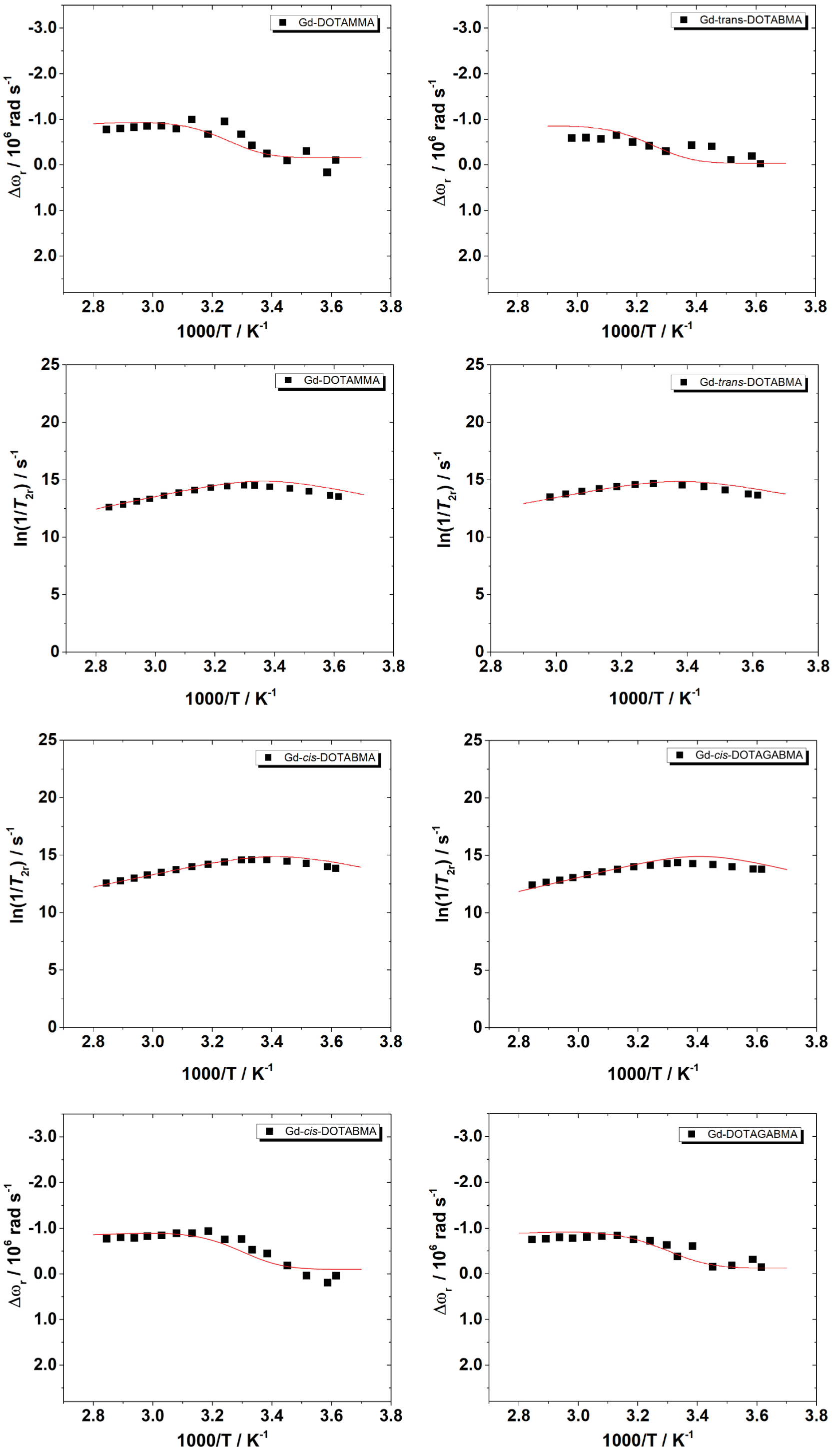

Figure S10. Graphs of the temperature dependence of the natural logarithm of the reduced transverse relaxation rate R_2 ($1/T_{2r}$) and of $\Delta\omega$.

4.6 NMRD Raw Data and Fitted NMRD Profiles of Linear P(NAM-r-Gd.L$_1$) polymers

Table S13. NMRD raw data P(NAM-r-1%-Gd.L$_1$).

NMRD Data - P(NAM-r-1%-Gd.L$_1$) - [Gd^{3+}] = 1.21 mM					
25 °C			37 °C		
Magnetic Field MHz	R$_1$[a] s^{-1}	r_1[b] mM^{-1} s^{-1}	Magnetic Field MHz	R$_1$[a] s^{-1}	r_1[b] mM^{-1} s^{-1}
0.00997	28.661	23.5675	0.00997	25.49	20.925
0.01436	28.062	23.26833	0.01436	25.427	20.8725
0.0207	28.894	23.36167	0.0207	25.924	21.28667
0.02973	28.082	23.085	0.02973	25.825	21.20417
0.04287	27.745	22.80417	0.04287	25.64	21.05
0.06162	28.263	23.23583	0.06162	25.764	21.15333
0.08854	28.232	23.21	0.08854	25.612	21.02667
0.12749	27.702	22.76833	0.12749	25.468	20.90667
0.18317	27.633	22.71083	0.18317	25.435	20.87917
0.26376	27.335	22.4625	0.26376	25.869	21.24083
0.37932	27.63	22.70833	0.37932	24.816	20.36333
0.54549	27.114	22.27833	0.54549	24.451	20.05917
0.7845	25.609	21.02417	0.7845	23.209	19.02417
1.1292	24.74	20.3	1.1292	22.526	18.455
1.6235	23.012	18.86	1.6235	21.042	17.21833
2.3363	21.412	17.52667	2.3363	19.001	15.5175
3.3598	19.06	15.66667	3.3598	17.007	13.85583
3.3598	19.212	15.69333	3.3598	17.368	14.05667
4.8321	18.434	15.345	4.8321	17.085	13.52083
6.9526	20.435	16.7125	6.9526	16.536	13.46333
9.9994	20.867	17.0725	9.9994	17.543	14.3025
21	22.77	18.65833	21	18.82	15.36667
31	22.48	18.41667	31	19.32	15.78333
41	21.59	17.675	41	18.64	15.21667
49	20.42	16.7	49	17.74	14.46667
61	19.34	15.8	61	16.54	13.46667
72	17.35	14.14167	72	15.13	12.29167

[a] Measured longitudinal relaxation rate. [b] Relaxivity calculated from the following equation: r_1 = (R$_1$ - $^{H_2O}r_1$) / [Gd^{3+}], $^{H_2O}r_1$ being the diamagnetic longitudinal relaxation rate of water (0.38 s^{-1} at 25°C, 0.28 s^{-1} at 37°C).

Table S14. NMRD raw data P(NAM-r-5%-Gd.L_1).

**NMRD Data - P(NAM-r-5%-Gd.L_1) - [Gd^{3+}] = 2.75 mM**					
**25 °C**			**37 °C**		
**Magnetic Field MHz**	**R_1[a] s^{-1}**	**r_1[b] mM^{-1} s^{-1}**	**Magnetic Field MHz**	**R_1[a] s^{-1}**	**r_1[b] mM^{-1} s^{-1}**
0.00997	59.6	21.55414	0.00997	52.5	19.00637
0.0144	57.8	20.899	0.0144	53	19.18835
0.0207	59	21.33576	0.0207	52.7	19.07916
0.0297	59.7	21.59054	0.0297	52.7	19.07916
0.0429	60.2	21.77252	0.0429	52.1	18.86078
0.0616	58.6	21.19017	0.0616	52.6	19.04277
0.0885	59.6	21.55414	0.0885	52.2	18.89718
0.127	59.4	21.48135	0.127	52	18.82439
0.183	59.7	21.59054	0.183	52.2	18.89718
0.264	58.8	21.26297	0.264	52	18.82439
0.379	58.5	21.15378	0.379	51.5	18.6424
0.545	56.8	20.53503	0.545	50.1	18.13285
0.785	54.9	19.84349	0.785	48.1	17.40491
1.13	51.5	18.60601	1.13	45.9	16.60419
1.62	48.4	17.47771	1.62	42.6	15.40309
2.34	44.5	16.05823	2.34	38.6	13.94722
3.36	40.4	14.56597	3.36	35.4	12.78253
4.83	39.8	14.34759	4.83	33	11.90901
6.95	40.1	14.45678	6.95	33.2	11.9818
10	42.4	15.2939	10	34.6	12.49136
21	47.1	17.00455	21	39.5	14.2748
31	47	16.96815	31	39.2	14.16561
41	45.3	16.34941	41	38	13.72884
49	43.6	15.73066	49	36.8	13.29208
61	40.9	14.74795	61	35	12.63694
72	38.2	13.76524	72	32.4	11.69063

[a] Measured longitudinal relaxation rate. [b] Relaxivity calculated from the following equation: r_1 = (R_1 - ${}^{H_2O}r_1$) / [Gd^{3+}], ${}^{H_2O}r_1$ being the diamagnetic longitudinal relaxation rate of water (0.38 s^{-1} at 25°C, 0.28 s^{-1} at 37°C).

Table S15. NMRD raw data P(NAM-r-9%-Gd.L_1).

**NMRD Data - P(NAM-r-9%-Gd.L_1) - [Gd^{3+}] = 3.00 mM**					
**25 °C**			**37 °C**		
**Magnetic Field** MHz	**R_1**[a] s^{-1}	r_1[b] mM^{-1} s^{-1}	**Magnetic Field** MHz	**R_1**[a] s^{-1}	r_1[b] mM^{-1} s^{-1}
0.00997	64.5	21.35198	0.00997	56.7	18.78788
0.0144	64.8	21.45188	0.0144	57.2	18.95438
0.0207	64.7	21.41858	0.0207	56.8	18.82118
0.0297	64.3	21.28538	0.0297	56.4	18.68798
0.0429	64.4	21.31868	0.0429	57.3	18.98768
0.0616	65.7	21.75158	0.0616	56.8	18.82118
0.0885	64.7	21.41858	0.0885	57.5	19.05428
0.127	64.6	21.38528	0.127	56.7	18.78788
0.183	64.9	21.48518	0.183	56.5	18.72128
0.264	63.7	21.08558	0.264	55.5	18.38828
0.379	62.5	20.68598	0.379	55.2	18.28838
0.545	61.4	20.31968	0.545	54.1	17.92208
0.785	59.4	19.65368	0.785	52.1	17.25608
1.13	56.7	18.75458	1.13	49.3	16.32368
1.62	53.1	17.55578	1.62	47.1	15.59108
2.34	49.2	16.25708	2.34	42.5	14.05927
3.36	43.8	14.45887	3.36	37.4	12.36097
4.83	43.2	14.25907	4.83	35.3	11.66167
6.95	44	14.52547	6.95	36.4	12.02797
10	46.8	15.45788	10	37.8	12.49417
21	51.6	17.05628	21	43.6	14.42557
31	51.6	17.05628	31	43.3	14.32567
41	50	16.52348	41	42	13.89277
49	47.9	15.82418	49	40.5	13.39327
61	44.8	14.79187	61	39	12.89377
72	41.8	13.79287	72	36	11.89477

[a] Measured longitudinal relaxation rate. [b] Relaxivity calculated from the following equation: r_1 = (R_1 - ${}^{H_2O}r_1$) / [Gd^{3+}], ${}^{H_2O}r_1$ being the diamagnetic longitudinal relaxation rate of water (0.38 s^{-1} at 25°C, 0.28 s^{-1} at 37°C).

Table S16. NMRD raw data P(NAM-r-17%-Gd.L_1).

NMRD Data - P(NAM-r-17%-Gd.L_1) - [Gd^{3+}] = 2.67 mM					
**25 °C**			**37 °C**		
**Magnetic Field MHz**	**R_1**[a] **s^{-1}**	r_1[b] **mM^{-1} s^{-1}**	**Magnetic Field MHz**	**R_1**[a] **s^{-1}**	r_1[b] **mM^{-1} s^{-1}**
0.00997	57.8	21.54597	0.00997	50.4	18.80675
0.0144	58.2	21.69606	0.0144	51.2	19.10694
0.0207	57.7	21.50844	0.0207	51.6	19.25704
0.0297	58.4	21.77111	0.0297	51.8	19.33208
0.0429	57.1	21.2833	0.0429	51.9	19.36961
0.0616	58.6	21.84615	0.0616	51.2	19.10694
0.0885	57.4	21.39587	0.0885	51.4	19.18199
0.127	58.2	21.69606	0.127	50.8	18.95685
0.183	57.4	21.39587	0.183	50.5	18.84428
0.264	57.5	21.4334	0.264	50.4	18.80675
0.379	56.7	21.13321	0.379	49.4	18.43152
0.545	55.2	20.57036	0.545	49.4	18.43152
0.785	53.7	20.0075	0.785	47	17.53096
1.13	51.9	19.33208	1.13	45.1	16.81801
1.62	48.5	18.05629	1.62	42.6	15.87992
2.34	45.2	16.81801	2.34	38.5	14.34146
3.36	40.8	15.16698	3.36	34.4	12.803
4.83	39.4	14.64165	4.83	32.5	12.09006
6.95	41.3	15.3546	6.95	33.2	12.35272
10	43.4	16.14259	10	35.3	13.14071
21	47.3	17.606	21	40.2	14.97936
31	47.3	17.606	31	40.3	15.01689
41	45.8	17.04315	41	39.1	14.5666
49	43.9	16.33021	49	37.6	14.00375
61	40.8	15.16698	61	35.4	13.17824
72	38.1	14.15385	72	33.3	12.39024

[a] Measured longitudinal relaxation rate. [b] Relaxivity calculated from the following equation: $r_1 = (R_1 - {}^{H_2O}r_1) / [Gd^{3+}]$, ${}^{H_2O}r_1$ being the diamagnetic longitudinal relaxation rate of water (0.38 s^{-1} at 25°C, 0.28 s^{-1} at 37°C).

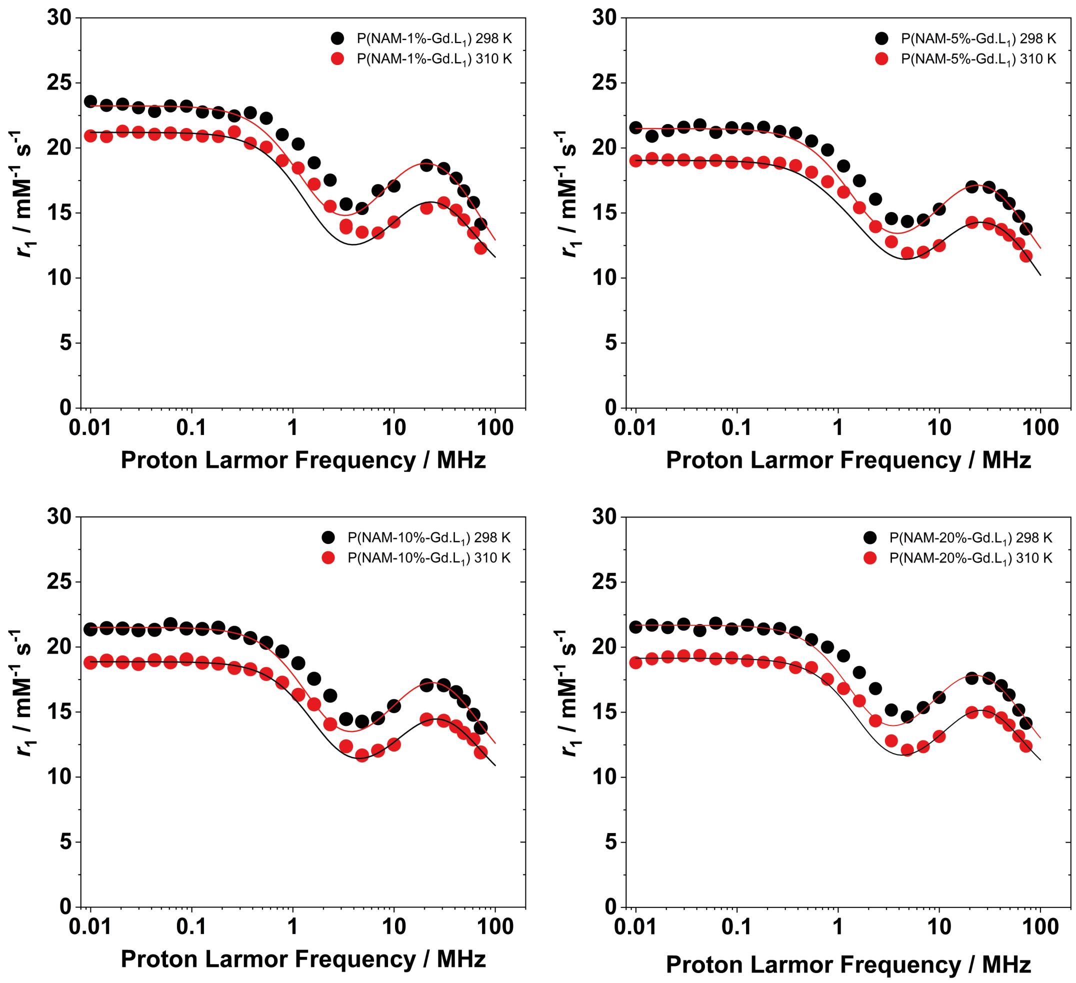

Figure S11. Fitted NMRD profiles of synthesised **Gd.L**$_{1-4}$ complexes at 25 and 37 °C. Solid red and black lines correspond to the fitting of data sets, respectively.

4.7 NMRD Raw Data and Fitted NMRD Profiles of Hyperbranched P(NAM-r-Gd.$L_{2–4}$) Polymers

Table S17. NMRD DATA P(NAM-r-0.9%-Gd.L_2).

NMRD Data - P(NAM-r-0.9%-Gd.L_2) - [Gd^{3+}] = 0.70 mM					
25 °C			37 °C		
Magnetic Field MHz	R_1 [a] s^{-1}	r_1 [b] mM^{-1} s^{-1}	Magnetic Field MHz	R_1 [a] s^{-1}	r_1 [b] mM^{-1} s^{-1}
0.00997	20.19	28.3	0.00997	19.742	27.66
0.01436	19.985	28.00714	0.01436	19.764	27.69143
0.0207	20.038	28.08286	0.0207	19.795	27.73571
0.02973	19.787	27.72429	0.02973	19.892	27.87429
0.04287	19.755	27.67857	0.04287	19.915	27.90714
0.06162	19.775	27.70714	0.06162	19.739	27.65571
0.08854	19.733	27.64714	0.08854	19.656	27.53714
0.12749	19.827	27.78143	0.12749	19.637	27.51
0.18317	19.738	27.65429	0.18317	19.414	27.19143
0.26376	19.103	26.74714	0.26376	19.309	27.04143
0.37932	18.998	26.59714	0.37932	18.941	26.51571
0.54549	18.201	25.45857	0.54549	18.34	25.65714
0.7845	17.844	24.94857	0.7845	17.516	24.48
1.1292	16.65	23.24286	1.1292	16.354	22.82
1.6235	15.825	22.06429	1.6235	15.429	21.49857
2.3363	14.789	20.58429	2.3363	14.369	19.98429
3.3598	13.815	19.19286	3.3598	13.315	18.47857
3.3598	13.161	18.25857	3.3598	13.212	18.33143
4.8321	13.622	18.91714	4.8321	13.085	18.15
6.9526	15.717	21.91	6.9526	14.754	20.53429
9.9994	17.767	24.83857	9.9994	17.206	24.03714
21	21.5	30.17143	21	21.48	30.14286
31	21.04	29.51429	31	21.26	29.82857
41	19.19	26.87143	41	19.26	26.97143
49	17.42	24.34286	49	17.41	24.32857
61	14.77	20.55714	61	14.88	20.71429
72	13.11	18.18571	72	13.2	18.31429

[a] Measured longitudinal relaxation rate. [b] Relaxivity calculated from the following equation: r_1 = (R_1 - ${}^{H_2O}r_1$) / [Gd^{3+}], ${}^{H_2O}r_1$ being the diamagnetic longitudinal relaxation rate of water (0.38 s^{-1} at 25°C, 0.28 s^{-1} at 37°C).

Table S18. NMRD DATA P(NAM-r-0.9%-Gd.L_3).

**NMRD Data - P(NAM-r-0.9%-Gd.L_3) - [Gd^{3+}] = 0.43 mM**					
**25 °C**			**37 °C**		
**Magnetic Field MHz**	**R_1[a] s^{-1}**	**r_1[b] mM^{-1} s^{-1}**	**Magnetic Field MHz**	**R_1[a] s^{-1}**	**r_1[b] mM^{-1} s^{-1}**
0.00997	15	34	0.00997	15.6	35.62791
0.0144	15	34	0.0144	15.6	35.62791
0.0207	14.9	33.76744	0.0207	15.8	36.09302
0.0297	14.9	33.76744	0.0297	15.6	35.62791
0.0429	14.8	33.53488	0.0429	15.7	35.86047
0.0616	14.8	33.53488	0.0616	15.7	35.86047
0.0885	15	34	0.0885	15.6	35.62791
0.127	14.7	33.30233	0.127	15.6	35.62791
0.183	14.7	33.30233	0.183	15.4	35.16279
0.264	14.6	33.06977	0.264	15.1	34.46512
0.379	14.1	31.90698	0.379	14.8	33.76744
0.545	13.8	31.2093	0.545	14.3	32.60465
0.785	13.3	30.04651	0.785	13.5	30.74419
1.13	12.7	28.65116	1.13	12.9	29.34884
1.62	12.2	27.48837	1.62	12.1	27.48837
2.34	11.2	25.16279	2.34	11	24.93023
3.36	10.2	22.83721	3.36	9.82	22.18605
4.83	9.9	22.13953	4.83	9.17	20.67442
6.95	10.7	24	6.95	10.3	23.30233
10	12.2	27.48837	10	11.9	27.02326
21	14.45	32.72093	21	14.7	33.53488
31	14.08	31.86047	31	14.4	32.83721
41	12.82	28.93023	41	13	29.5814
49	11.52	25.90698	49	11.8	26.7907
61	9.86	22.04651	61	10.1	22.83721
72	8.56	19.02326	72	8.7	19.5814

[a] Measured longitudinal relaxation rate. [b] Relaxivity calculated from the following equation: r_1 = (R_1 - ${}^{H_2O}r_1$) / [Gd^{3+}], ${}^{H_2O}r_1$ being the diamagnetic longitudinal relaxation rate of water (0.38 s^{-1} at 25°C, 0.28 s^{-1} at 37°C).

Table S19. NMRD DATA P(NAM-r-0.9%-Gd.L_4).

**NMRD Data - P(NAM-r-0.9%-Gd.L_4) - [Gd^{3+}] = 0.48 mM**					
**25 °C**			**37 °C**		
**Magnetic Field MHz**	**R_1[a] s^{-1}**	**r_1[b] mM^{-1} s^{-1}**	**Magnetic Field MHz**	**R_1[a] s^{-1}**	**r_1[b] mM^{-1} s^{-1}**
0.00997	15.5	31.5	0.00997	14.5	29.625
0.0144	15.5	31.5	0.0144	14.5	29.625
0.0207	15.5	31.5	0.0207	14.4	29.41667
0.0297	15.6	31.70833	0.0297	14.5	29.625
0.0429	15.5	31.5	0.0429	14.5	29.625
0.0616	15.4	31.29167	0.0616	14.4	29.41667
0.0885	15.4	31.29167	0.0885	14.2	29
0.127	15.2	30.875	0.127	14.3	29.20833
0.183	14.9	30.25	0.183	14.3	29.20833
0.264	14.8	30.04167	0.264	14	28.58333
0.379	14.5	29.41667	0.379	13.7	27.95833
0.545	13.8	27.95833	0.545	13.4	27.33333
0.785	13	26.29167	0.785	12	24.41667
1.13	12.3	24.83333	1.13	11	22.33333
1.62	11.2	22.54167	1.62	10.1	20.45833
2.34	10.3	20.66667	2.34	9.05	18.27083
3.36	9.7	19.41667	3.36	8.5	17.125
4.83	9.42	18.83333	4.83	8.27	16.64583
6.95	10.3	20.66667	6.95	8.78	17.70833
10	11.2	22.54167	10	9.73	19.6875
21	12.87	26.02083	21	11.3	22.95833
31	12.75	25.77083	31	11.2	22.75
41	12.21	24.64583	41	10.6	21.5
49	11.5	23.16667	49	10.1	20.45833
61	10.41	20.89583	61	9.2	18.58333
72	9.5	19	72	8.6	17.33333
72	13.11	18.18571	72	13.2	18.31429

[a] Measured longitudinal relaxation rate. [b] Relaxivity calculated from the following equation: r_1 = (R_1 - ${}^{H_2O}r_1$) / [Gd^{3+}], ${}^{H_2O}r_1$ being the diamagnetic longitudinal relaxation rate of water (0.38 s^{-1} at 25°C, 0.28 s^{-1} at 37°C).

4.8 Fitted NMRD profiles and NMRD Parameters obtained for the Partial Fitting (1-100 MHz) of Hyperbranched Systems NMRD Data Sets

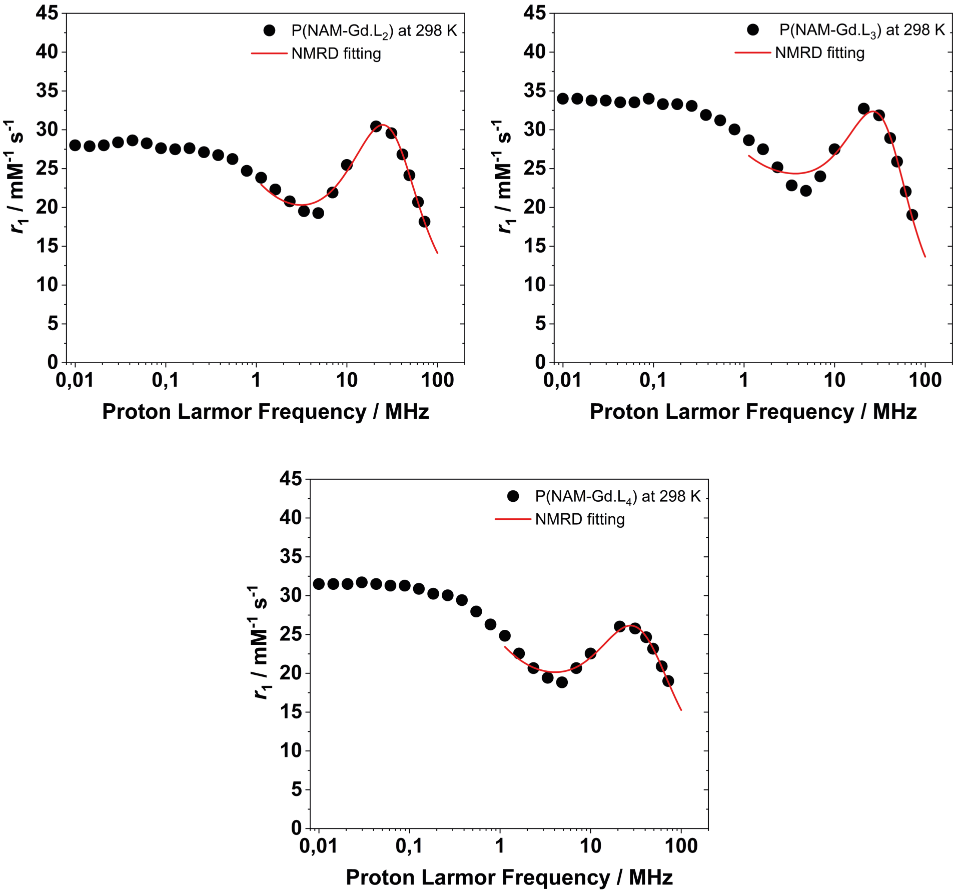

Figure S12. NMRD profiles of partially fitted NMRD profiles of hyperbranched systems, *i.e.* between 1 to 100 MHz.

Table S20. NMRD parameters of partially fitted NMRD profiles of hyperbranched systems, *i.e*. between 1 to 100 MHz.

NMRD parameters obtained from the partial fitting of hyperbranched systems NMRD data set, for high magnetic field (1 to 100 MHz)			
**NMRD Best fit parameters**	**P(NAM-Gd.L$_2$)**	**P(NAM-Gd.L$_3$)**	**P(NAM-Gd.L$_4$)**
Δ^2 **/ 10^{18} s^{-2}**	6.2 ± 0.2	6.3 ± 0.4	5.7 ± 0.3
$^{298}\tau_V$ **/ ps**	16.1 ± 0.9	10.4 ± 0.7	12.6 ± 0.8
$^{298}\tau_{RL}$ **/ ps**	501 ± 45	418 ± 99	447 ± 34
$^{298}\tau_{RG}$ **/ ps**	6366 ± 486	6668 ± 812	3810 ± 256
S^2	0.500	0.601	0.403
$^{298}\tau_M{}^a$ **/ ns**	326 ± 8	308 ± 12	315 ± 9

To fit the ^{1}H NMRD data at 298 K, the following parameters were fixed: $q = 1$, $r_{Gd-H} = 3.0$ Å, $a_{Gd-H} = 4$ Å, $^{298}D_{Gd-H} = 2.25 \times 10^{-5}$ cm^2 s^{-1}. ^{a} Estimated from VT NMR relaxivity profiles (Figure 5) at fixed magnetic field (20 MHz).

4.9 Fitted NMRD profiles and NMRD parameters obtained for the fitting of Hyperbranched Systems over the all magnetic field range 0.01-100 MHz NMRD Data Sets

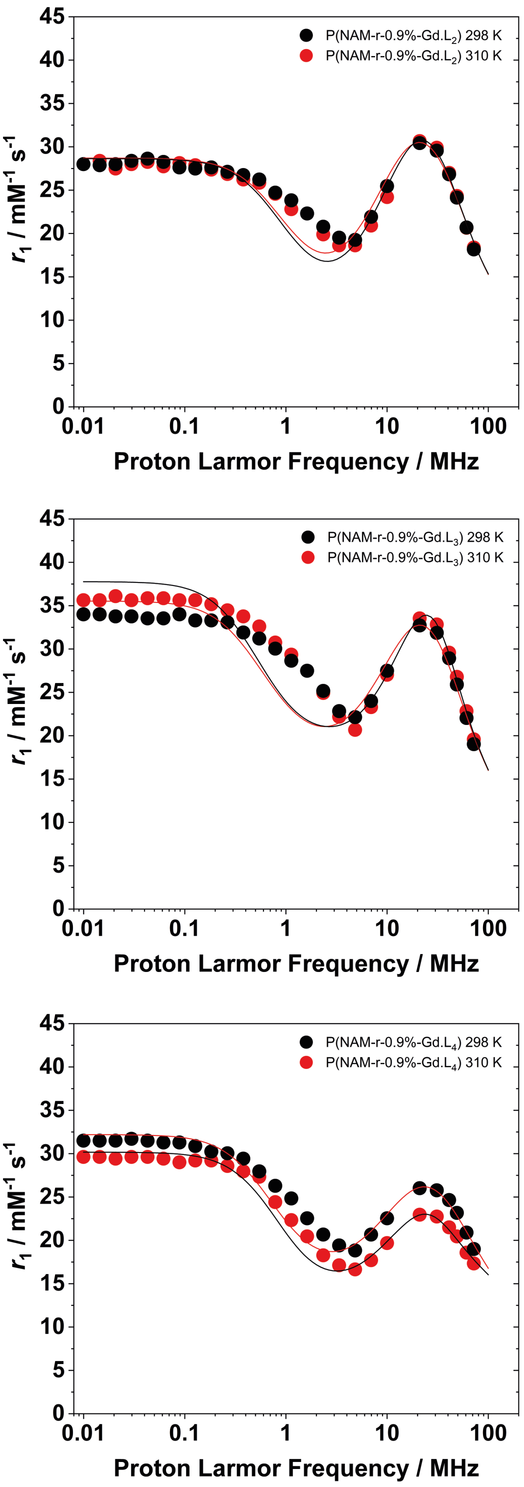

Figure S13. Fitted NMRD profiles of synthesised hyperbranched **P(NAM-r-Gd.L$_{2-4}$)** systems at 25 and 37 °C. Solid red and black lines correspond to the fitting of data sets.

Table S21. NMRD parameters obtained from the fitting of hyperbranched systems NMRD profiles between 0.01-100 MHz.

**NMRD parameters obtained from the partial fitting of hyperbranched systems NMRD data set, for high magnetic field (1 to 100 MHz)**			
**NMRD Best fit parameters**	**P(NAM-Gd.L$_2$)**	**P(NAM-Gd.L$_3$)**	**P(NAM-Gd.L$_4$)**
Δ^2 / 10^{18} s^{-2}	6.4 ± 0.2	5.6 ± 0.1	5.22 ± 0.06
$^{298}\tau_V$ / ps	29 ± 1	23.1 ± 0.5	23.5 ± 0.3
$^{298}\tau_{RL}$ / ps	595 ± 67	731 ± 45	690 ± 14
$^{298}\tau_{RG}$ / ps	5600 ± 800	6200 ± 500	4000 ± 200
S^2	0.458	0.601	0.403
$^{298}\tau_M{}^a$ / ns	326 ± 8	308 ± 12	318 ± 3

To fit the ^{1}H NMRD data at 298 K, the following parameters were fixed: q = 1, r_{Gd-H} = 3.0 Å, a_{Gd-H} = 4 Å, $^{298}D_{Gd-H}$ = 2.25 ×10^{-5} cm^2 s^{-1}. [a] Estimated from VT NMR relaxivity profiles (Figure 5) at fixed magnetic field (20 MHz).

We have fitted the NMRD profiles in Figure 5 (B3) to the high field region (1-100 MHz), and the data for all hyperbranced polymers is presented in the ESI (Figure S12) together with the parameters used to perform the fitting (in the main text in Tables 4 and 5, and in Table S20 of the ESI). We agree that the poor fit of the data in the region 0.5-5 MHz is a consequence of the limitations in relaxation theory, specifically limitations in the SBM theory and of the Lipari-Szabo approach to account for the electron spin relaxation of slowly rotating molecules at these Larmor frequencies.[25] We note that parameters obtained by fitting only the high field data (Figure S12 & Table S20) very similar to those obtained when fitting over the entire Larmor frequency range (Figure S13 and Table S21). Only small differences in the electronic parameters and in the local correlation time were observed, as expected since the fitting of the low field region is necessary to establish reliable electronic parameters. However, the conclusions we draw from the fitting are the same.

4.10 VT Profiles of Linear Polymeric Systems

Table S22. Variable temperature profiles of polymeric systems P(NAM-r-1%-Gd.L_1) and P(NAM-r-17%-Gd.L_1).

VT Profile at 20 MHz - P(NAM-r-1%-Gd.L_1) - [Gd^{3+}]= 1.21 mM				
**Temperature °C**	**Temperature K**	$^{H_2O}r_1{}^a$ **$mM^{-1}s^{-1}$**	**$R_1{}^b$ s^{-1}**	$r_1{}^c$ **$mM^{-1}s^{-1}$**
4.2	277.35	0.62966	20.99	16.96695
11.7	284.85	0.52317	22.81	18.57235
18.7	291.85	0.4401	23.7	19.38325
23.4	296.55	0.39186	23.3	19.09012
30.3	303.45	0.33045	21.81	17.89963
37.1	310.25	0.27935	19.78	16.25054
43.3	316.45	0.23968	17.4	14.30027
50.3	323.45	0.20162	15.11	12.42365
55.9	329.05	0.17557	13.21	10.86202
62.1	335.25	0.15064	11.64	9.57447
66	339.15	0.13681	10.73	8.82766
70.8	343.95	0.12151	9.63	7.92374

VT Profile at 20 MHz - P(NAM-r-17%-Gd.L_1) - [Gd^{3+}]= 2.665 mM				
**Temperature °C**	**Temperature K**	$^{H_2O}r_1{}^a$ **$mM^{-1}s^{-1}$**	**$R_1{}^b$ s^{-1}**	$r_1{}^c$ **$mM^{-1}s^{-1}$**
4.1	277.25	0.61734	42.78567	15.82301
10.4	283.55	0.54561	46.523	17.2523
15.1	288.25	0.48222	48.43525	17.99363
20.3	293.45	0.42619	48.677	18.10537
24.8	297.95	0.37667	47.42067	17.65253
30.1	303.25	0.3329	44.76733	16.67333
34.9	308.05	0.29422	41.59067	15.49585
40.1	313.25	0.26004	37.838	14.10055
45.2	318.35	0.22982	34.182	12.74003
50.2	323.35	0.20312	30.711	11.44761
55.1	328.25	0.17952	27.55425	10.27194
60.1	333.25	0.15866	24.79925	9.246
65.4	338.55	0.14023	21.983	8.19616
70.2	343.35	0.12393	19.8485	7.40134

^{a} $^{H_2O}r_1$ is the diamagnetic longitudinal relaxation rate of water obtain with empirical equation: $^{H_2O}r_1 = (0.9756^T) \times 0.6985$ with T in °C. ^{b} Measured longitudinal relaxation rate. ^{c} Relaxivity calculated from the following equation: $r_1 = (R_1 - {}^{H_2O}r_1) / [Gd^{3+}]$.

4.11 VT Profiles of Hyperbranched Polymeric Systems

Table S23. Variable temperature profiles of hyperbranched polymeric systems P(NAM-r-Gd.L_{2-3}).

**VT Profile 20 MHz - P(NAM-r-0.9%-Gd.L_2) - [Gd^{3+}]= 0.70 mM**				
**Temperature °C**	**Temperature K**	$^{H_2O}r_1{}^a$ **$mM^{-1}s^{-1}$**	**$R_1{}^b$ s^{-1}**	$r_1{}^c$ **$mM^{-1}s^{-1}$**
4.2	0.62966	277.35	16.04	22.01477
11.7	0.52317	284.85	18.36	25.48118
18.7	0.4401	291.85	20.73	28.98557
23.4	0.39186	296.55	22.13	31.05449
30.3	0.33045	303.45	22.77	32.0565
37.1	0.27935	310.25	22.26	31.40093
43.3	0.23968	316.45	21.14	29.8576
50.3	0.20162	323.45	19.39	27.41197
55.9	0.17557	329.05	17.7	25.03489
62.1	0.15064	335.25	15.88	22.47051
66	0.13681	339.15	14.73	20.84742
70.8	0.12151	343.95	13.61	19.26927
65.4	0.13885	338.55	4.631	6.32697
69.7	0.12486	342.85	4.22	5.76781
**VT Profile 20 MHz - P(NAM-r-0.9%-Gd.L_3) - [Gd^{3+}]= 0.43 mM**				
**Temperature °C**	**Temperature K**	$^{H_2O}r_1{}^a$ **$mM^{-1}s^{-1}$**	**$R_1{}^b$ s^{-1}**	$r_1{}^c$ **$mM^{-1}s^{-1}$**
3.8	0.63592	276.95	9.96475	21.69496
10.2	0.54292	283.35	11.37325	25.18681
15.1	0.48103	288.25	12.70225	28.42145
20.4	0.422	293.55	13.87267	31.28062
24.9	0.3776	298.05	14.53225	32.91779
29.9	0.33373	303.05	14.977	34.05412
34.9	0.29495	308.05	14.94833	34.07763
39.9	0.26068	313.05	14.54475	33.21876
45.1	0.22926	318.25	13.891	31.77149
50.2	0.20212	323.35	13.07675	29.941
55	0.17952	328.15	12.25533	28.08328
60.4	0.1571	333.55	11.17	25.61139
65.6	0.13816	338.75	10.224	23.45543
70.4	0.12272	343.55	9.35333	21.46655

[a] $^{H_2O}r_1$ is the diamagnetic longitudinal relaxation rate of water obtain with empirical equation: $^{H_2O}r_1 = (0.9756^T) \times 0.6985$ with T in °C. [b] Measured longitudinal relaxation rate. [c] Relaxivity calculated from the following equation: $r_1 = (R_1 - {}^{H_2O}r_1) / [Gd^{3+}]$.

Table S24. Variable temperature profiles of hyperbranched polymeric systems P(NAM-r-Gd.L_4).

**VT Profile 20 MHz - P(NAM-r-0.9%-Gd.L_4) - [Gd^{3+}]= 0.48 mM**				
**Temperature**	**Temperature**	$^{H_2O}r_1{}^a$	$\mathbf{R_1}^b$	$r_1{}^c$
**°C**	**K**	$\mathbf{mM^{-1}s^{-1}}$	$\mathbf{s^{-1}}$	$\mathbf{mM^{-1}s^{-1}}$
3.8	0.63592	276.95	13.20475	26.18507
10.2	0.54292	283.35	13.36567	26.71405
14.9	0.48341	288.05	13.47067	27.05678
20.4	0.422	293.55	13.36375	26.96198
24.8	0.37854	297.95	12.954	26.19888
29.9	0.33373	303.05	12.36733	25.07001
34.9	0.29495	308.05	11.63567	23.62649
39.9	0.26068	313.05	10.78	21.91525
45.2	0.22869	318.35	9.87033	20.08675
50.1	0.20262	323.25	9.04067	18.4126
54.9	0.17996	328.05	8.22533	16.76118
60.4	0.1571	333.55	7.34167	14.96784
65.8	0.13748	338.95	6.62075	13.50681
70.3	0.12302	343.45	6.0035	12.251

^{a} $^{H_2O}r_1$ is the diamagnetic longitudinal relaxation rate of water obtain with empirical equation: $^{H_2O}r_1$ = $(0.9756^T) \times 0.6985$ with T in °C. ^{b} Measured longitudinal relaxation rate. ^{c} Relaxivity calculated from the following equation: r_1 = (R_1 - $^{H_2O}r_1$) / [Gd^{3+}].

4.12 Discussion over VT Profiles

The analysis presented in Figure 5 (C1-C3), is a combination of ^{17}O data (C1) and variable temperature data (C2 and C3). Unfortunately, we were unable to carry out ^{17}O analysis of the polymers as the concentration of Gd(III) complex required for such experiments is greater than 5 mM, significantly higher than the Gd(III) concentrations used to prepare the polymers. In order to provide reliable estimates of water residence lifetimes we fitted the VT ^1H NMR profiles for the linear and hyperbranched polymers.

It is not possible to compare the position of the peaks observed in the ^{17}O plot in C1 with the VT ^1H NMR profiles in C2 and C3. However, we are able to compare the VT profiles obtained for the linear and crosslinked polymers. In doing so, it is clear that the temperature dependence in the ^1H NMR spectra of the linear polymers containing Gd.L_1 closely resembles that of the crosslinked polymer containing Gd.L_4, with profile peaks observed around 290 K. In contrast, for the crosslinked polymers containing Gd.L_2 or Gd.L_3, the VT profile peaks at higher temperatures of around 310 K. Considering that a similar rate of the water exchange was estimated for all of the linear and crosslinked polymers (308 – 330 ns, see Table 5), the shift

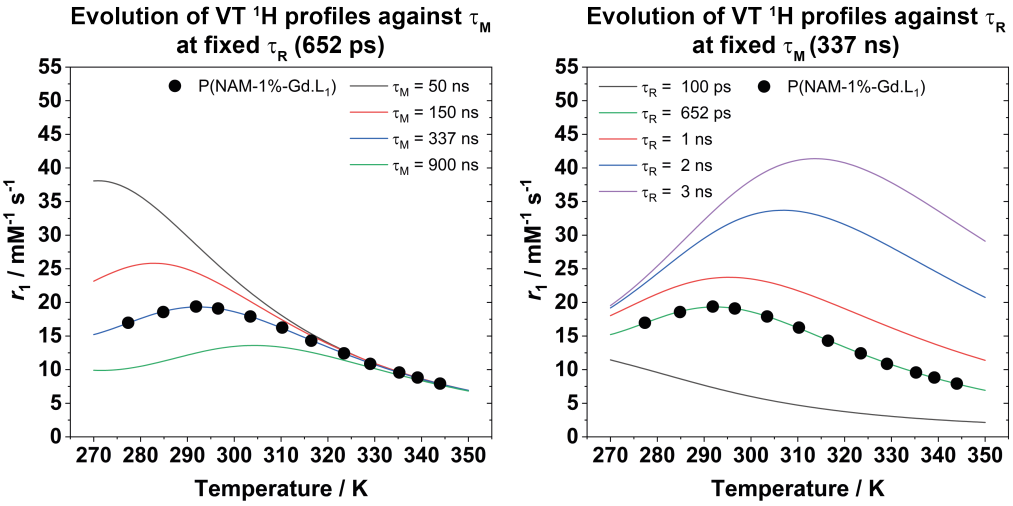

Figure S14. Using the experimental VT profile of P(NAM-1%-Gd.L_1) (•), simulation of the evolution of the VT profiles shape depending on: (left) a change of water residence time τ_M, or (right) rotational correlation time τ_R, while keeping all other fitting parameters constant. S = 3.5, F = 21, $\Delta^2 = 4.38\times10^{18}$ s^{-2}, τ_V = 42.1 ps, E_V = 1 KJ mol^{-1}, τ_M = 337 ns, ΔH_M = 36 KJ mol^{-1}, τ_R = 652 ps, E_R = 20 KJ mol^{-1}, q = 1, r_{Gd-H} = 3.0 Å, a_{Gd-H} = 4.0 Å, $D_0 = 2.24\times10^{-5}$ cm^2 s^{-1}, $H_D = -3.09\times10^4$.

in the VT profile peaks to higher temperature for the crosslinked polymers of Gd.L_2 or Gd.L_3 may be reasonably attributed to the slower local and global rotational correlation times of these systems. This is consistent with the higher order parameter ($S^2 \approx 0.55$) determined for the crosslinked polymers of Gd.L_2 or Gd.L_3, which we discuss on page 9 of the manuscript. We considered whether it was possible to correlate a shift in the temperature at which the VT profile peaks with either an increase or decrease of τ_M or τ_R, but concluded that it is not possible, since both parameters influence the relaxivity maximum of the VT profiles. To demonstrate this, in the figure below (Figure S14) we have simulated some VT profiles from our experimental data (a linear polymer of P(NAM-r-Gd.L1)), which represent the independent influence of tumbling and water exchange rate on the temperature at which the VT profile peaks. A decrease in τ_M does lead to a shift of the VT profile maximum towards lower temperature. However, increasing τ_R generates the opposite effect. Thus, the shift of VT profile maximum will depend on which parameter dominates the relaxivity at the magnetic field studied. For our slowly tumbling macromolecules, it is reasonable to assume that τ_R is the primary factor influencing the maximum of the VT profile peak. Through these simulations, we are able to validate our estimation of the water exchange rates.

5 Theoretical Models to Explain Paramagnetic Relaxation Enhancement

5.1 Discrete Complex NMRD Profiles - the Solomon-Bloembergen-Morgan Theory

Here, are summarised the theory and the equations necessary for the analysis of a 1H NMRD profile. First, the observed longitudinal proton relaxation rate, in a solution of paramagnetic molecules, is the sum of a diamagnetic contribution R_1^D (proton relaxation rate of pure solvent) plus the paramagnetic contribution to the relaxation rate induced by the paramagnetic molecule R_1^P. This paramagnetic contribution is expressed as the longitudinal relaxivity times r_1 the concentration of paramagnetic centres , here [Gd^{3+}] (**Equation 3**).

$$R_1^{obs} = R_1^D + R_1^P = R_1^D + r_1[Gd^{3+}] \tag{3}$$

The longitudinal proton relaxivity measured by relaxometry is also the results of two distinct contributions: the inner-sphere IS and outer-sphere OS relaxivity. Thus relaxivity can be describe by **Equation 4**.

$$r_1 = r_1^{IS} + r_1^{OS} \tag{4}$$

The IS contribution is defined by **Equation 5** when normalised to 1 mM Gd concentration, with q the number of water molecules simultaneity bound to the paramagnetic centre, τ_m the residence time of an inner-sphere water molecule and $T_{1\mathrm{m}}^H$ the relaxation time of inner-sphere protons.

$$r_1^{\mathrm{IS}} = \frac{1}{1000} \times \frac{q}{55.55} \times \frac{1}{T_{1\mathrm{m}}^H + \tau_{\mathrm{m}}} \tag{5}$$

Then, $1/T_{1\mathrm{m}}^H$, the longitudinal relaxation rate of the inner sphere protons can be expressed following **Equation 6**, where r_{GdH} is the distance between Gd and inner-sphere water protons, ω_I is the proton resonance frequency, ω_S is the Larmor frequency of the Gd electron spin ($\omega_S = 658\omega_I$), S is the electron spin, γ_S is the electron gyromagnetic ratio and γ_I is the nuclear gyromagnetic ratio, μ_0 is the permeability constant, $\hbar$ reduced Planck constant and the correlation times τ_{di} can be expressed by **Equation 7**.

$$\frac{1}{T_{1m}^{H}} = \frac{2}{15}\left(\frac{\mu_0}{4\pi}\right)^2 \frac{\hbar^2\gamma_1^2\gamma_S^2}{r_{GdH}^6} S(S+1) \times \left[\frac{3\tau_{d1}}{1+\omega_I^2\tau_{d1}^2} + \frac{7\tau_{d2}}{1+\omega_S^2\tau_{d2}^2}\right] \tag{6}$$

$$\frac{1}{\tau_{di}} = \frac{1}{\tau_m} + \frac{1}{\tau_R} + \frac{1}{T_{ie}} \quad i = 1,2 \tag{7}$$

The longitudinal and transverse electronic relaxation rates, $1/T_{1e}$ and $1/T_{2e}$ are estimated by **Equation 8** and **Equation 9** respectively. In these equations, Δ^2 is the mean square zero-field-splitting energy and τ_V is the electronic correlation time for the modulation of the ZFS interaction.

$$\frac{1}{T_{1e}} = \frac{1}{25}\Delta^2\tau_v[4S(S+1)-3] \times \left(\frac{1}{1+\omega_S^2\tau_v^2} + \frac{4}{1+4\omega_S^2\tau_v^2}\right) \tag{8}$$

$$\frac{1}{T_{2e}} = \frac{1}{50}\Delta^2\tau_v[4S(S+1)-3] \times \left(\frac{5}{1+\omega_S^2\tau_v^2} + \frac{2}{1+4\omega_S^2\tau_v^2} + 3\right) \tag{9}$$

The correlation times τ_V and τ_R used in previous equations are temperature dependent and can be described following Arrhenius laws (**Equations 10**), where E_V and E_R are the associated activation energies.

$$\tau_V = \tau_V^{298} \exp\left[\frac{E_V}{R}\left(\frac{1}{T} - \frac{1}{298.15}\right)\right] \qquad \tau_R = \tau_R^{298} \exp\left[\frac{E_R}{R}\left(\frac{1}{T} - \frac{1}{298.15}\right)\right] \tag{10}$$

The second term of the longitudinal relaxivity is the outer-sphere relaxivity than can be described with **Equation 11**.

$$r_1^{OS} = \frac{32N_A\pi}{405}\left(\frac{\mu_0}{4\pi}\right)^2 \frac{\hbar^2\gamma_S^2\gamma_I^2}{a_{GdH}D_{GdH}} S(S+1)\left[3J_{os}(\omega_I; T_{1e}) + 7J_{os}(\omega_S; T_{2e})\right] \tag{11}$$

In this equation, N_A is the Avogadro constant, a_{GdH} is the minimum distance between the water molecule and the complex, D_{GdH} is the sum of the diffusion coefficients of water away from a Gd complex, τ_{GdH} is a diffusion correlation time and is equal to $\tau_{GdH} = a_{GdH}^2/D_{GdH}$ and J_{OS} is the realpart of spectral density function described in **Equation 12** with $j = 1, 2$.

$$J_{os}\left(\omega, T_{je}\right) = Re\left[\frac{1 + \frac{1}{4}\left(i\omega\tau_{GdH} + \frac{\tau_{GdH}}{T_e}\right)^{\frac{1}{2}}}{1 + \left(i\omega\tau_{GdH} + \frac{\tau_{GdH}}{T_e}\right)^{\frac{1}{2}} + \frac{4}{9}\left(i\omega\tau_{GdH} + \frac{\tau_{GdH}}{T_e}\right) + \frac{1}{9}\left(i\omega\tau_{GdH} + \frac{\tau_{GdH}}{T_e}\right)^{\frac{3}{2}}}\right] \tag{12}$$

Like τ_V and τ_R, D_{GdH} is temperature dependant and is following an Arrhenius law (**Equation 13** characterised by the activation energy E_{DGdH}.

$$D_{\mathrm{GdH}} = D_{GdH}^{298} \exp\left\{\frac{E_{DGdH}}{R}\left(\frac{1}{298.15} - \frac{1}{T}\right)\right\} \tag{13}$$

5.2 Macromolecules NMRD Profiles - the Lipari-Szabo Model

As the SBM-theory neglects the effects induced by macromolecular or large molecular weight paramagnetic systems, it limited the analysis of the NMRD profiles of such systems. To overcome, this limitation, a variation of the SBM-theory was developed by Giovanni Lipari and Attila Szabo. The approach distinguish two different motions, the fast local motion of a paramagnetic moiety represented by a local correlation time τ_{RL}, attached to a slow motion molecule characterised by a global correlation time τ_{RG}. The model is also defined by an order parameter S^2, which describes the degree of restriction of the local motion by the rest of the molecule. If $S^2 = 0$, both motions are independent and if $S^2 = 1$ both motion are totally correlated. With the Lipari-Szabo, the longitudinal relaxation rate of the inner-sphere protons is now expressed by **Equations 14**.

$$\begin{aligned}\frac{1}{T_{1\mathrm{m}}^{H}} =& \frac{2}{15}\left(\frac{\mu_0}{4\pi}\right)^2 \frac{\gamma_I^2 g^2 \mu_B^2}{r_{\mathrm{GdH}}^6} S(S+1)\left[\frac{3S^2\tau_{d1g}}{1+\omega_I^2\tau_{d1g}^2}\right. \\ &\left. + \frac{3\left(1-S^2\right)\tau_{d1}}{1+\omega_I^2\tau_{d1}^2} + \frac{7S^2\tau_{d2g}}{1+\omega_I^2\tau_{d2g}^2} + \frac{7\left(1-S^2\right)\tau_{d2}}{1+\omega_I^2\tau_{d2}^2}\right]\end{aligned} \tag{14}$$

With τ_{di}, τ_{di} and τ defined respectively in **Equations 15**, 16, 17

$$\frac{1}{\tau_{\mathrm{di}}} = \frac{1}{\tau_{\mathrm{m}}} + \frac{1}{\tau} + \frac{1}{T_{\mathrm{ie}}} \quad i = 1,2 \tag{15}$$

$$\frac{1}{\tau_{\mathrm{dig}}} = \frac{1}{\tau_{\mathrm{m}}} + \frac{1}{\tau_{\mathrm{RG}}} + \frac{1}{T_{\mathrm{ie}}} \quad i = 1,2 \tag{16}$$

$$\frac{1}{\tau} = \frac{1}{\tau_{\mathrm{RG}}} + \frac{1}{\tau_{\mathrm{RL}}} \tag{17}$$

References

[1] Townsend, T. R.; Moyle-Heyrman, G.; Sukerkar, P. A.; MacRenaris, K. W.; Burdette, J. E.; Meade, T. J. *Bioconjugate Chem.* **2014**, *25*, 1428–1437.

[2] Srivastava, P.; Tiwari, A. K.; Chadha, N.; Chuttani, K.; Mishra, A. K. *Eur. J. Med. Chem* **2013**, *65*, 12–20.

[3] Rigaux, G.; Roullin, V. G.; Cadiou, C.; Portefaix, C.; Van Gulick, L.; Bœuf, G.; Andry, M. C.; Hoeffel, C.; Vander Elst, L.; Laurent, S.; Muller, R.; Molinari, M.; Chuburu, F. *Nanotechnology* **2014**, *25*, 445103.

[4] Wang, K.; Chen, S.; Zhang, W. *Macromolecules* **2017**, *50*, 4686–4698.

[5] Wang, L.; Lin, H.; Ma, L.; Sun, C.; Huang, J.; Li, A.; Zhao, T.; Chen, Z.; Gao, J. *J. Mater. Chem. B* **2017**, *5*, 8004–8012.

[6] Harris, M.; Vander Elst, L.; Laurent, S.; Parac-Vogt, T. N. *Dalton Trans.* **2016**, *45*, 4791–4801.

[7] Li, C.; Wong, W.-T. *J. Org. Chem.* **2003**, *68*, 2956–2959.

[8] Kovacs, Z.; Sherry, A. D. *ChemComm* **1995**, 185.

[9] Kovacs, Z.; Sherry, A. D. *Synthesis* **1997**, *1997*, 759–763.

[10] Adhikari, A.; Kumari, N.; Adhikari, M.; Kumar, N.; Tiwari, A. K.; Shukla, A.; Mishra, A. K.; Datta, A. *Bioorg. Med. Chem.* **2017**, *25*, 3483–3490.

[11] Hopper, L. E.; Allen, M. J. *Tetrahedron Lett.* **2014**, *55*, 5560–5561.

[12] Hirayama, T.; Taki, M.; Kodan, A.; Kato, H.; Yamamoto, Y. *ChemComm* **2009**, 3196.

[13] Butler, S. J. *ChemComm* **2015**, *51*, 10879–10882.

[14] Samuels, E. R.; Sevrioukova, I. F. *Tetrahedron Lett.* **2018**, *59*, 1140–1142.

[15] Moumne, R.; Lavielle, S.; Karoyan, P. *J. Org. Chem.* **2006**, *71*, 3332–3334.

[16] Tka, N.; Kraïem, J.; Hassine, B. B. *Synth. Commun.* **2013**, *43*, 735–743.

[17] Souers, A. J.; Schürer, S.; Kwack, H.; Virgilio, A. A.; Ellman, J. A. *Synthesis* **1999**, *4*, 583–585.

[18] Sim, N.; Pal, R.; Parker, D.; Engelmann, J.; Mishra, A.; Gottschalk, S. *Org. Biomol. Chem.* **2014**, *12*, 9389–9404.

[19] Kielar, F.; Tei, L.; Terreno, E.; Botta, M. *J. Am. Chem. Soc.* **2010**, *132*, 7836–7837.

[20] Alb, A. M.; Enohnyaket, P.; Drenski, M. F.; Shunmugam, R.; Tew, G. N.; Reed, W. F. *Macromolecules* **2006**, *39*, 8283–8292.

[21] Pei, X.; Zan, T.; Li, H.; Chen, Y.; Shi, L.; Zhang, Z. *ACS Macro Lett.* **2015**, *4*, 1215–1219.

[22] Lu, M.; Wang, Y.-K.; Zhao, J.; Lu, H.; Stenzel, M. H.; Xiao, P. *Macromol. Rapid Commun.* **2016**, *37*, 2023–2029.

[23] Dey, P.; Rajdev, P.; Pramanik, P.; Ghosh, S. *Macromolecules* **2018**, *51*, 5182–5190.

[24] Corsi, D. M.; Platas-Iglesias, C.; Bekkum, H. v.; Peters, J. A. *Magn. Reson. Chem.* **2001**, *39*, 723–726.

[25] Jaszberenyi, Z.; Moriggi, L.; Schmidt, P.; Weidensteiner, C.; Kneuer, R.; Merbach, A. E.; Helm, L.; Toth, E. *J. Biol. Inorg. Chem.* **2007**, *12*, 406–420.
